# Plasmacytoid dendritic cells control homeostasis of megakaryopoiesis

**DOI:** 10.1038/s41586-024-07671-y

**Published:** 2024-07-10

**Authors:** Florian Gaertner, Hellen Ishikawa-Ankerhold, Susanne Stutte, Wenwen Fu, Jutta Weitz, Anne Dueck, Bhavishya Nelakuditi, Valeria Fumagalli, Dominic van den Heuvel, Larissa Belz, Gulnoza Sobirova, Zhe Zhang, Anna Titova, Alejandro Martinez Navarro, Kami Pekayvaz, Michael Lorenz, Louisa von Baumgarten, Jan Kranich, Tobias Straub, Bastian Popper, Vanessa Zheden, Walter Anton Kaufmann, Chenglong Guo, Guido Piontek, Saskia von Stillfried, Peter Boor, Marco Colonna, Sebastian Clauß, Christian Schulz, Thomas Brocker, Barbara Walzog, Christoph Scheiermann, William C. Aird, Claus Nerlov, Konstantin Stark, Tobias Petzold, Stefan Engelhardt, Michael Sixt, Robert Hauschild, Martina Rudelius, Robert A. J. Oostendorp, Matteo Iannacone, Matthias Heinig, Steffen Massberg

**Affiliations:** 1 Department of Medicine I, University Hospital, LMU Munich, Munich, Germany; 2 Institute of Science and Technology Austria (ISTA), Klosterneuburg, Austria; 3 DZHK (German Centre for Cardiovascular Research), Partner site Munich Heart Alliance, Munich, Germany; 4 Institute of Cardiovascular Physiology and Pathophysiology, Biomedical Center, LMU Munich, Planegg-Martinsried, Germany; 5 Walter Brendel Center of Experimental Medicine, University Hospital, LMU Munich, Munich, Germany; 6 Institute for Immunology, Faculty of Medicine, LMU Munich, Munich, Germany; 7 Institute of Pharmacology and Toxicology, Technical University of Munich (TUM), Munich, Germany; 8 Institute of Computational Biology, Deutsches Forschungszentrum für Gesundheit und Umwelt, Helmholtz Zentrum München, Neuherberg, Germany; 9 Department of Computer Science, TUM School of Computation, Information and Technology, Technical University of Munich, Garching, Germany; 10 Division of Immunology, Transplantation and Infectious Diseases, IRCCS San Raffaele Scientific Institute, Milan, Italy; 11 Department of Dynamics of Immune Responses, Vita-Salute San Raffaele University, Milan, Italy; 12 Department of Neurology, Ludwig-Maximilians-University School of Medicine, Munich, Germany; 13 Biomedical Center, Bioinformatic Core facility, LMU Munich, Planegg-Martinsried, Germany; 14 Biomedical Center, Core Facility Animal Models, LMU Munich, Planegg-Martinsried, Germany; 15 Institute of Pathology, Ludwig-Maximilians-University Munich, Munich, Germany; 16 Institute of Pathology, RWTH Aachen University Hospital, Aachen, Germany; 17 Washington University, School of Medicine, St Louis, MO USA; 18 Department of Pathology and Immunology, Faculty of Medicine, University of Geneva, Geneva, Switzerland; 19 Department of Medicine, Center for Vascular Biology Research, Beth Israel Deaconess Medical Center, Boston, MA USA; 20 MRC Molecular Haematology Unit, MRC Weatherall Institute of Molecular Medicine, University of Oxford, John Radcliffe Hospital, Oxford, UK; 21 Department of Cardiology, Angiology and Intensive Care Medicine, Campus Benjamin Franklin, Deutsches Herzzentrum der Charité (DHZC) University Hospital Berlin, Berlin, Germany; 22 DZHK (German Centre for Cardiovascular Research), Partner site Berlin, Berlin, Germany; 23 Friede Springer - Centre of Cardiovascular Prevention @ Charité, Charité - University Medicine Berlin, Berlin, Germany; 24 Laboratory of Stem Cell Physiology, Department of Internal Medicine III—Hematology and Oncology, Klinikum rechts der Isar, School of Medicine, Technical University of Munich, Munich, Germany

**Keywords:** Haematopoiesis, Cardiovascular biology

## Abstract

Platelet homeostasis is essential for vascular integrity and immune defence^1,2^. Although the process of platelet formation by fragmenting megakaryocytes (MKs; thrombopoiesis) has been extensively studied, the cellular and molecular mechanisms required to constantly replenish the pool of MKs by their progenitor cells (megakaryopoiesis) remains unclear^3,4^. Here we use intravital imaging to track the cellular dynamics of megakaryopoiesis over days. We identify plasmacytoid dendritic cells (pDCs) as homeostatic sensors that monitor the bone marrow for apoptotic MKs and deliver IFNα to the MK niche triggering local on-demand proliferation and maturation of MK progenitors. This pDC-dependent feedback loop is crucial for MK and platelet homeostasis at steady state and under stress. pDCs are best known for their ability to function as vigilant detectors of viral infection^5^. We show that virus-induced activation of pDCs interferes with their function as homeostatic sensors of megakaryopoiesis. Consequently, activation of pDCs by SARS-CoV-2 leads to excessive megakaryopoiesis. Together, we identify a pDC-dependent homeostatic circuit that involves innate immune sensing and demand-adapted release of inflammatory mediators to maintain homeostasis of the megakaryocytic lineage.

## Abstract (web summary)

Plasmacytoid dendritic cells monitor the bone marrow for apoptotic megakaryocytes (MKs) and deliver IFNα to the MK niche, triggering local on-demand proliferation and maturation of MK progenitors.

## Main

Platelets are anucleate cells circulating in the blood to maintain vascular barrier function in health and disease^1,2^. They are produced in the bone marrow (BM) by their precursors, MKs, in a process called thrombopoiesis^3^. During thrombopoiesis, MKs show signs of apoptosis^6^ and release platelets in a process in which the MK cell body is entirely consumed^7^. Consequently, replenishment of fragmented MKs from progenitors (megakaryopoiesis) is continuously required to ensure MK homeostasis and sustained platelet production.

Here we identify a homeostatic circuit^8^ that balances thrombopoiesis and megakaryopoiesis in BM tissue. Patrolling pDCs—a unique subset of innate immune sentinel cells^5^—sense MK turnover by detecting cell-free DNA released from apoptotic MKs. Innate immune signalling through the MYD88–IRF7 pathway activates the release of IFNα by pDCs, which in turn triggers megakaryopoiesis to replenish MKs and to maintain platelet homeostasis during steady state and stress. Thus, our data establish innate immune sensing by pDCs as a key mechanism controlling cellular homeostasis in the BM and blood. Perturbed pDC function, such as strong activation during viral infection with SARS-CoV-2, increases megakaryopoiesis, leading to marked hyperplasia of the megakaryocytic lineage. Our data may therefore provide a mechanistic explanation for alterations in platelet counts frequently observed during inflammation and infection and opens routes for therapeutic intervention.

## Cellular dynamics of megakaryopoiesis

**Fig. 1:**
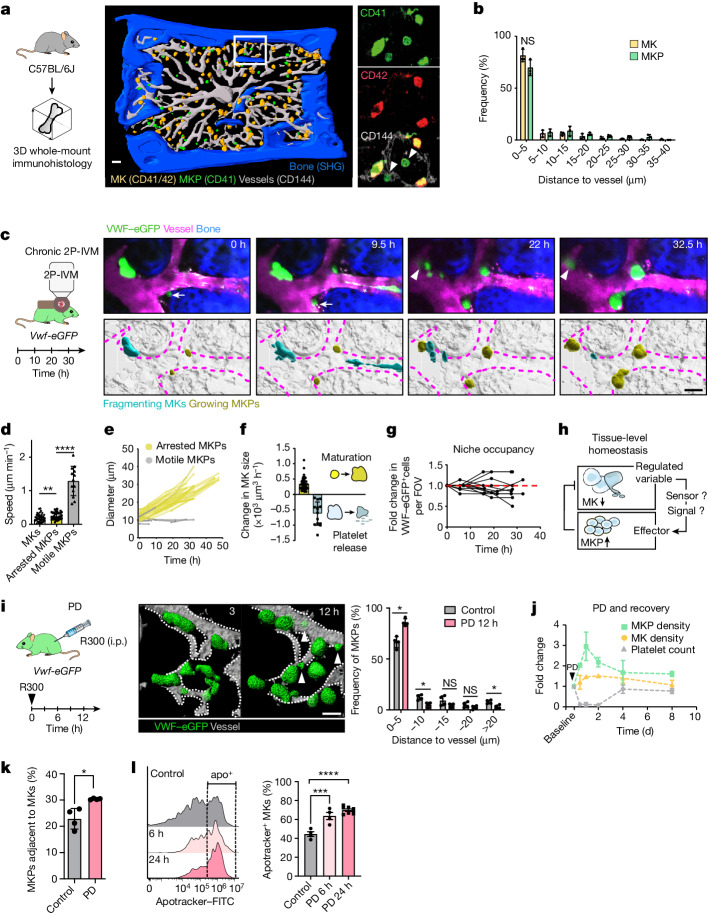
Spatiotemporal coordination of thrombopoiesis and megakaryopoiesis. **a**, 3D-rendered *z* stack of mouse BM (sternum). *n* = 3. MKPs (green): CD41^+^CD42^−^; MKs (yellow): CD41^+^CD42^+^; sinusoids (grey): CD144^+^; bone (blue): second harmonic generation. **b**, The distribution of MKs and MKPs relative to their distance to sinusoids. *n* = 3. Data are mean ± s.d. Statistical analysis was performed using multiple unpaired *t*-tests; NS, not significant. **c**, Chronic 2P-IVM analysis of the calvaria. *n* = 7 mice. Top, images of *Vwf*^*eGFP/+*^ cells (green); TRITC–dextran (sinusoids; magenta). The arrow indicates an MKP migrating at the perivascular niche before growth. The arrowhead indicates MKP growth in the proximity of thrombopoiesis (which is indicated by an asterisk). Bottom, 3D-rendering. **d**, The speed of MKs (*n* = 52), arrested MKPs (*n* = 33) and motile MKPs (*n* = 31). Cells were pooled from 7 mice. Statistical analysis was performed using one-way ANOVA with Tukey’s test; NS, *P* = 0.2029; ****P = 0.0000000005. Data are mean ± s.d. **e**, The diameters of arrested and motile MKPs tracked over time (2P-IVM). *n* = 28 cells from 5 mice (arrested MKPs) and *n* = 6 cells from 3 mice (motile MKPs). **f**, The change in cell volume per hour during MKP maturation (growth; yellow) (*n* = 14 cells pooled from 4 mice) and platelet release (reduction; cyan) (*n* = 11 cells pooled from 4 mice). Data are mean ± s.d. **g**, *Vwf*^*eGFP/+*^ cells per field of view (FOV) tracked over time. *n* = 12 FOVs from 5 mice. **h**, The homeostatic circuit of MKs in BM. **i**, Chronic 2P-IVM after PD. The histogram shows an increased frequency of MKPs at the perivascular niche. *n* = 4 mice per group. Statistical analysis was performed using multiple Mann–Whitney *U*-tests; *P = 0.0286. Data are mean ± s.d. Arrowheads, new MK progenitors. **j**, The fold change in platelet counts (haemocytometer). *n* = 9 (baseline), *n* = 6 (0.5 days), *n* = 8 (1 day), *n* = 9 (2 days), *n* = 9 (4 days), *n* = 4 (8 days) mice. MK/MKP density (counts per mm^3^) (BM whole-mount immunostainings) (*n* = 3 mice) were measured at the indicated timepoints after PD. **k**, The percentage of MKPs attached to MKs (BM whole-mount immunostainings). *n* = 4 mice. Statistical analysis was performed using an unpaired *t*-test; **P = 0.009. Data are mean ± s.d. **l**, The frequency of apoptotic MKs (live/dead-stain-405^−^CD41-PE^+^CD42-APC^+^CD11b^−^CD8a^−^Apotracker green^+^) increases after PD (light pink, 6 h; pink, 24 h), as determined using FACS. Left, the fluorescence intensity (Apotracker). The frequency of apotracker^+^ MKs. *n* = 4 (control and PD (6 h)) and *n* = 6 (PD (12 h)) mice. Statistical analysis was performed using one-way ANOVA with Tukey’s test; ***P = 0.00041; ****P = 0.0000059. Data are mean ± s.d. For **a**, **c** and **i**, scale bars, 50 μm. Source data

**Extended Data Fig. 1:**
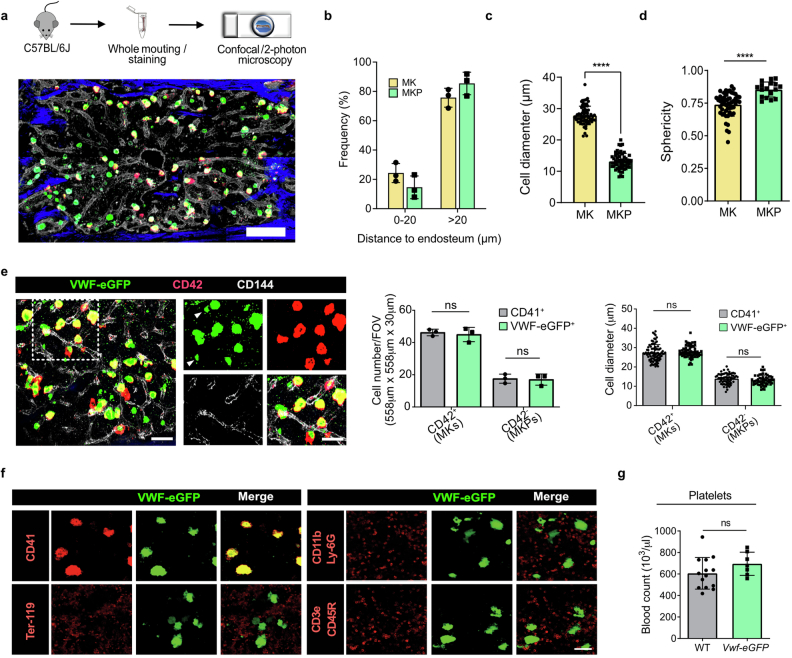
MK and MKP distribution in the bone marrow niche. **a**, Representative whole-mount immunostaining of megakaryocyte progenitors and mature megakaryocytes in murine sternum bone (n = 3 mice). MKPs (green): CD41^+^/CD42^−^; MKs (yellow): CD41^+^/CD42^+^; blood vessels (grey): CD144^+^; bone (blue): second harmonic generation. Scale bars = 200 μm (upper). Also see Fig. 1a. **b**, Histogram showing BM distribution of MKs and MKPs relative to their distance to endosteum. n = 3 mice; Mean ± SD. **c**, **d**, Cell diameter and sphericitity; for cell diameter MK n = 68 cells and MKP n = 55 cells; for sphericity MK n = 53 cells and MKP n = 17 cells; pooled from 7 mice; ****(Sphericity): p = 0.0000002, ****(Cell diameter): p = 0.000000000000001; unpaired t-test/Welch’s correction; Mean ± SD. **e**, Representative whole-mount immunostaining of MKs/MKPs in *Vwf*^*eGFP/+*^ mice. MKs/MKPs of *Vwf*^*eGFP/+*^ mice show no significant difference in cell number (p = 0.69/p = 0.84) and size (p = 0.38/p = 0.10) compared to C57Bl/6 J mice (stained with anti-CD41). n = 3 mice; unpaired t-test/Welch’s correction; Mean ± SD. Scale bar = 30 μm. **f**, VWF-eGFP does not co-localize with erythrocytes (Ter-119), granulocytes (CD11b, Ly-6G) and lymphocytes (CD3e, CD45R) in BM (n = 1). Scale bar = 100 µm. **g**, Platelet counts of *Vwf*^*eGFP/+*^ compared to C57Bl/6 J; WT group n = 14 mice, *Vwf*^*eGFP/+*^ group n = 7 mice. unpaired t-test/Welch’s correction; Mean ± SD; ns: p = 0.14. Source data

The primary site of megakaryopoiesis in mammals is the BM^3^. To analyse the spatial distribution of MKs (CD41^+^CD42^+^) and their progenitors (MKPs) (CD41^+^CD42^−^) we performed three-dimensional immunofluorescence imaging of mouse calvarial BM^9^ (Fig. 1a, Extended Data Fig. 1a and Supplementary Video 1). The vast majority of mature MKs (around 82%) resides within a distance of ≤5 µm to sinusoids (Fig. 1b) without preferential association to the endosteum or other sites (Extended Data Fig. 1b). MKPs were significantly smaller than MKs (Extended Data Fig. 1c), were largely spherical (Extended Data Fig. 1d) and showed a similar distribution to mature MKs (around 70% of MKPs) (Fig. 1a,b).

**Extended Data Fig. 2:**
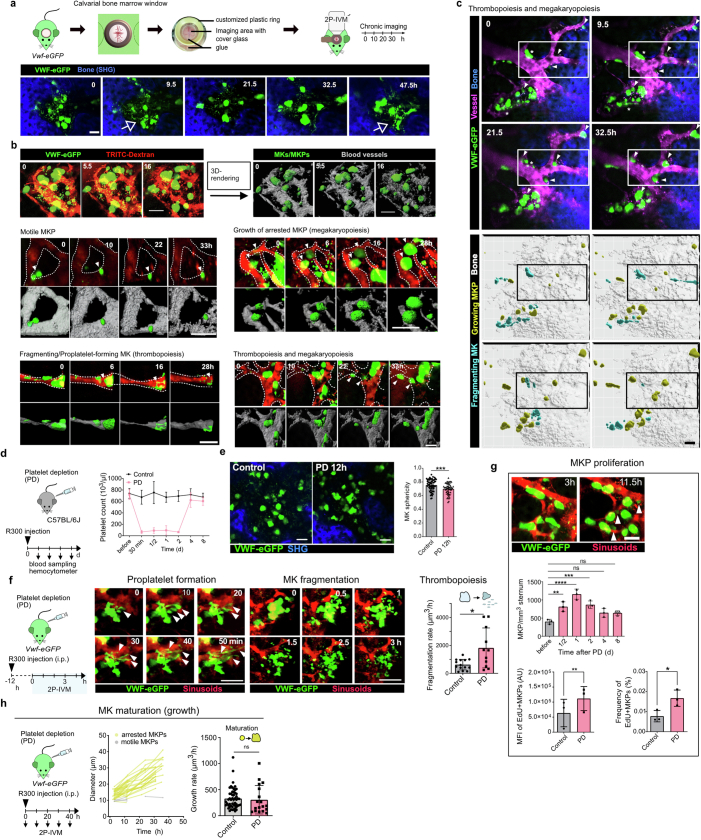
Megakaryocytic lineage tracing by chronic time-lapse 2P-IVM. **a**, Schematic showing experimental setup of chronic 2-photon intravital microscopy (2P-IVM) of mouse calvarian bone marrow. Lower: Time series of BM in *Vwf*^*eGFP/+*^ mice. MK lineage (VWF-eGFP; green); Bone (second harmonic generation). Arrow: Fragmentation of MK results in the release of platelet-like particles. Note that after fragmentation, platelet-like particles exit the BM and new MKs grow in size before undergoing fragmentation. **b**, *Vwf*^*eGFP/+*^ mice were i.v. injected with TRITC-dextran to track the megakaryocytic lineage (green) and blood vessels (red) respectively (left time series). Max. intensity projections of raw data and 3D rendering of z-stacks from the same 2P-IVM time series are shown. Scale bars = 50 μm. **c**, Representative raw data and 3D-rendering corresponding to 3D-rendered images shown in Fig. 1c (n = 7). Scale bars = 50 µm. **d-h:** Spatiotemporal dynamics of the megakaryocytic lineage in response to immune-mediated thrombocytopenia. **d**, Schematic showing experimental setup of PD. Peripheral blood platelet counts monitored at indicated time points after a single injection (i.p.) of R300 or isotype control; R300: before (n = 9), 30 min (n = 3), 0.5d (n = 6), 1d (n = 8), 2d (n = 9), 4d (n = 9), 8d (n = 4) mice; isotype: before (n = 7), 30 min (n = 3), 0.5d (n = 4), 1d (n = 5), 2d (n = 7), 4d (n = 4), 8d (n = 3) Mean ± SD. **e**, Morphometric analysis of MKs shows decreased sphericity in response to PD corresponding to an increase of cellular protrusions; Control n = 63 cells and PD 12 h n = 51 cells, pooled from 4 mice; unpaired t-test/Welch’ correction, ***: p = 0.0002; Mean ± SD. Scale bars = 50 μm. **f**, Representative 2P-IVM time series of proplatelet formation and MK fragmentation. Single cell tracking of MK volumes over time reveals a significant faster decrease of volume following PD. Control n = 14 and PD n = 11 cells, pooled from 3 mice; unpaired t-test; ns: p = 0.0206; Mean ± SD. **g**, Upper: Small ( < 15 μm) VWF-eGFP+ cells (arrows) appear 12 h after PD (2P-IVM). Also see Fig. 1i. Scale bar = 50 µm. Middle: PD triggers an instantaneous proliferation of MKPs peaking 1d following platelet depletion; MKPs (CD41^+^/CD42^–^) were counted in whole-mount BMs. n = 3 mice; one-way ANOVA/Dunnett, **: p = 0.0023, ****: p = 0.000009, ***: p = 0.0008, ns: p = 0.063 and p = 0.072; Mean ± SD. Lower: Proliferation of MKPs (VWF-eGFP^+^/CD41^+^/CD42^–^) was measured after in vivo labelling of BM cells with 5-ethynyl-2′-deoxyuridine (EdU) using FACS. Mean fluorescent intensity of EdU and Frequency of EdU-pve cells significantly increases after PD (12 h); n = 3 mice; paired t-test; **: p = 0.0046; *: p = 0.0115; error bar=SD; Mean ± SD. **h**, Left: MKPs lodged within the perivascular niche of the BM grow in volume. Volume increase of single cells was tracked over time. Arrested MKP n = 27 cells and mobile MKP n = 6 cells. Notably, the speed of cell growth after PD did not significantly differ from steady state control; Control n = 54 cells and PD = 18 cells, pooled from 3 mice, unpaired t-test Welch’s correction ns: p = 0.7503; Mean ± SD. Source data

To study the spatiotemporal patterns of megakaryopoiesis in vivo, we performed two-photon intravital microscopy (2P-IVM) analysis of *Vwf-eGFP* reporter mice, specifically labelling the entire megakaryocytic lineage including MKPs and mature MKs^10^ (Extended Data Fig. 1e–g). We visualized the same field of view for up to 3 days with an imaging window implanted onto the calvaria to track individual MKPs and MKs (Extended Data Fig. 2a). We identified small, motile *Vwf*^*eGFP/+*^ cells within the BM parenchyma that arrest along BM sinusoids (Fig. 1c and Extended Data Fig. 2b,c) and increase their volume by around tenfold (Fig. 1c–f and Supplementary Video 2), representing MKPs undergoing cytoplasmic maturation into large, sessile MKs (Fig. 1d). This provides real-time evidence that perivascular positioning of immotile MKs is determined by their motile progenitors, as previously proposed by others^11^.

Mature MKs lodged within the perivascular niche release proplatelets to produce platelets^3^ (Fig. 1c and Extended Data Fig. 2b). Once entering thrombopoiesis MKs rapidly reduce their volume and disappear completely within hours (Fig. 1c,f and Extended Data Fig. 2b,c). Consumption of platelet-producing MKs is irreversible as we did not observe recovery once MKs completed thrombopoiesis. Instead, new *Vwf*^*eGFP/+*^ MKPs appear in proximity to vanished MKs giving rise to mature MKs (Fig. 1c, Extended Data Fig. 2b,c and Supplementary Video 2). Consequently, the total number of *Vwf*^*eGFP/+*^ cells within one field of view remains highly stable over several hours to days (Fig. 1g). At the BM-tissue level megakaryopoiesis and thrombopoiesis are therefore well synchronized processes that ensure immediate replenishment of platelet-producing MKs from their progenitors to maintain MK homeostasis (Fig. 1h).

We tested whether megakaryopoiesis and thrombopoiesis also remain synchronized in situations of high platelet demand. We removed the entire circulating platelet pool by antibody-mediated platelet depletion (PD) (Extended Data Fig. 2d). During PD, MKs lose sphericity, indicating activation (Extended Data Fig. 2e), and engage in emergency platelet production through intrasinusoidal proplatelet extensions or MK fragmentation resulting in a rapid reduction in MK size (Extended Data Fig. 2f). Multi-day four-dimensional imaging revealed that the fast release of platelets from MKs is accompanied by an increased MKP proliferation that peaks at 12–24 h after treatment and was most prominent at the perivascular niche, while MK growth dynamics was not affected (Fig. 1i and Extended Data Fig. 2g,h). Accelerated proliferation of MKPs fully compensated for the high MK demand during emergency thrombopoiesis and replenished the circulating platelet pool within 4 days while maintaining MK homeostasis (Fig. 1j). Together, our data show that thrombopoiesis and megakaryopoiesis are tightly coordinated to maintain MK homeostasis in BM tissue both in steady state and pathological platelet consumption, raising the question of the underlying mechanism^8^ (Fig. 1h).

## pDCs regulate megakaryopoiesis

**Extended Data Fig. 3:**
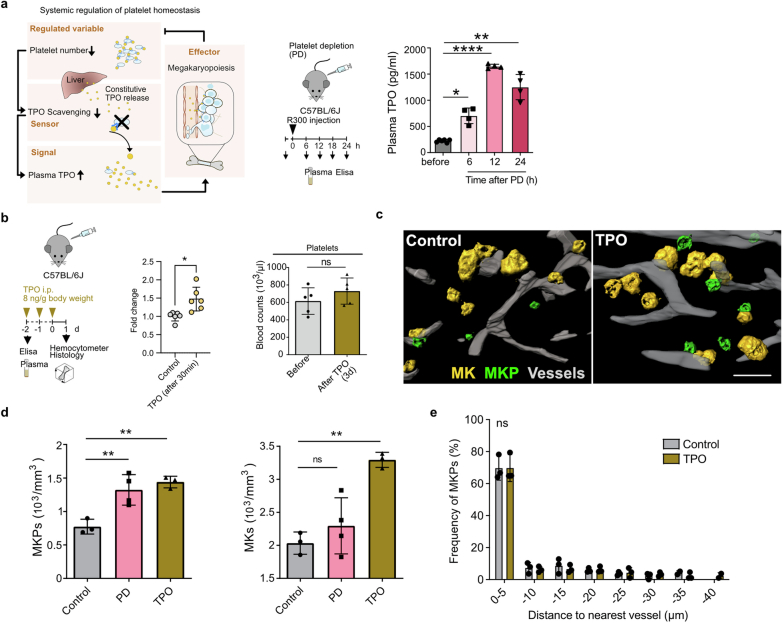
TPO triggers global megakaryopoiesis without preferential localization to the perivascular niche. **a**, Left: Scheme of platelet homeostasis regulated by TPO. TPO released from liver is scavenged by circulating platelets that express the TPO-receptor (Mpl). Thrombocytopenia leads to an increase of unbound plasma TPO which drives megakaryopoiesis in the BM. Right: Plasma TPO levels increase in response to PD, reaching the highest levels 12 h after platelet depletion (ELISA); n = 4 mice per group, one-way ANOVA/Dunnett; *: p = 0.018, **: p = 0.008, ****: p = 0.00000005; Mean ± SD. **b**, Left: Plasma TPO levels increase after i.p. injection (8 ng/g body weight on 3 consecutive days, i.p.) (ELISA after 30 min) (n = 6 mice); Mean ± SD; unpaired t-test/Welch’s correction; *: p = 0.014. Right: Thrombopoiesis is not affected by TPO treatment as indicated by unaffected platelet counts (hemocytometer); n = 5 mice, unpaired t-test Welch’s correction ns: p = 0.267; Mean ± SD. **c**, 3D-rendered micrographs of BM wholemount staining show increased numbers of MKPs (green: CD41 + / < 20 μm) and MKs (yellow: CD41^+^/ > 20 μm) after TPO treatment, vessels (grey: CD144); scale bar = 50 µm. **d**, TPO-treatment increased megakaryopoiesis (MKP and MK numbers) to an extent similar to platelet depletion (PD). Of note, TPO-treatment leads to an accumulation of mature MKs in the BM while MK numbers in the BM remained unaffected after PD due to increased MK consumption (BM whole-mount immunostainings); control n = 3 mice, PD n = 4 mice and TPO n = 3 mice, one-way ANOVA/Dunnett; MKPs, **(PD): p = 0.0064, **(TPO): p = 0.0032; MK, ns(PD) = 0.44, **(TPO): p = 0.0023; Mean ± SD. **e**, Increased megakaryopoiesis in response to TPO followed a different spatial pattern compared to PD. During PD, the local increase in megakaryopoiesis is restricted to the perisinusoidal compartment (see Fig. 1g). In contrast, TPO treatment increased megakaryopoiesis throughout the BM compartment. Consequently, the distribution of MKPs relative to the perivascular niche was unaffected by TPO treatment as analysed in BM whole-mount immunostaining. The distances were binned into 5 μm intervals; n = 4 mice per group; Unpaired t-test; ns: p = 0.99; Mean ± SD. Source data

Liver-derived thrombopoietin (TPO) is the most potent cytokine promoting megakaryopoiesis. Its plasma levels are tightly regulated through TPO sequestration by TPO receptors (cMPL) on circulating platelets^12^ (Extended Data Fig. 3a). Elevated plasma TPO levels drove global proliferation of BM MKPs, but did not trigger characteristic local perivascular megakaryopoiesis (Extended Data Fig. 3b–e) as observed after PD (Fig. 1i). Consistent with these data, both TPO- and cMPL-deficient mice have been shown to produce small numbers of morphologically and functionally normal MKs and platelets at steady state^13^, and were able to produce normal platelet counts in response to stress^14^. This suggests that additional TPO-independent signals are involved, potentially arising locally from the BM niche^15^.

**Extended Data Fig. 4:**
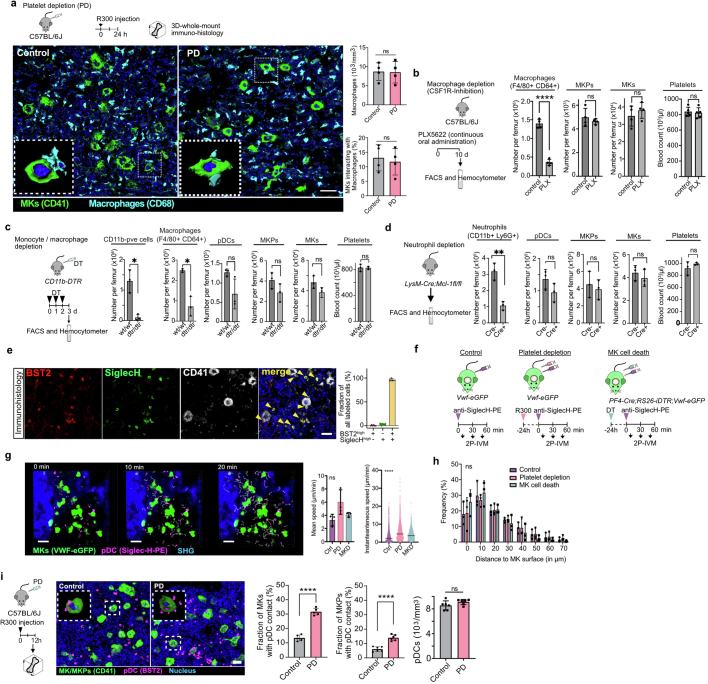
MK-immune cell interaction in the bone marrow. **a-d**: Macrophages, monocytes and neutrophils are dispensable for megakaryopoiesis **a**, Left: MK-Macrophage contacts were quantified by whole-mount BM immunohistology; MK: CD41^+^; Macrophages: CD68^+^. Scale bar = 10 µm. Right: PD did not increase MK-Macrophage contacts; n = 4 mice; unpaired t-test Welch’s correction ns: p = 0.9299 and ns: p = 0.6882; Mean ± SD. **b**, Macrophage depletion with Csf1R-Inhibitor (PLX5622) did not impair megakaryopoiesis (n = 4 mice). Mean ± SD; Welch’s t test; ****: p = 0.000018. **c**, Macrophage / monocyte depletion in *CD11b-DTR* mice did not impair megakaryopoiesis (n = 3 mice). Mean ± SD; Welch’s t test; *: p = 0.02. **d**, Neutrophil-depletion in *LysM-Cre; Mcl-1fl/fl* mice has no impact on megakaryopoiesis (n = 3 mice). Mean ± SD; Welch’s t test; **: p = 0.009. **e-i:** pDCs reside in close proximity to bone marrow MKs. **e**, Whole mount histology of BM. MKs: CD41 (grey); pDCs: BST2 (red) and SiglecH (green). The vast majority of bright SiglecH-positive cells also show bright BST2 signal, indicative of pDCs. **f**, Experimental setup of 2P-IVM and platelet depletion (PD) and MK cell death (MKD). **g**, 2P-IVM of calvaria bone. MKs: VWF-eGFP (green); pDCs: SiglecH-PE (magenta). Also see Fig. 2a. pDCs migrate with mean speeds of 3-6 μm/min, without significant alteration by platelet depletion of MK cell death. Left: Mean of 3 experiments is plotted (each data-point represents one mouse). Right: Mean of all pooled frames (each data point represents speed at a single frame pooled from 3 experiments). Mean ± SD; one-way ANOVA/Tukey; ns: p = 0.07; ****: p = 0.0000000000000001. Scale bar: 100 μm. **h**, Histogram showing the distribution of pDCs relative to their distance from MKs (n = 3 mice per group); Mean ± SD; multiple unpaired t-tests; ns: no statistical significance. **i**, Whole mount histology of control and PD. MKs (green, CD41); pDCs (magenta, BST2^+^); nuclei (blue, Hoechst). Bar plot: frequencies of pDC-MK- and pDC-MKP contacts; n≥5 mice; unpaired Welch’s t-test; ****(%MK with pDC-contacts): p = 0.000002, ****(%MKP with pDC-contacts): p = 00003; Mean ± SD. PD did not increase the number of bone marrow pDCs (n = 6 mice). Mean ± SD; unpaired t-test Welch’s correction; ns: no statistical significance. Scale bars = 50 µm. Source data

Whole-mount analysis of mouse BM showed that a considerable fraction of MKPs was located in close proximity to mature MKs^16^ (Fig. 1a,k). A large proportion of mature MKs showed signs of apoptosis, and the apoptotic MK fraction further increased when we induced emergency thrombopoiesis by depleting platelets (Fig. 1l). On the basis of these two observations, we hypothesized that vanishing MKs that release platelets and show signs of apoptosis^6^ may trigger their own replacement from local MKPs within the perivascular niche. Different phagocyte subsets are equipped to sense and clear apoptotic bodies and cell-free DNA. In particular, macrophages have an important role in homeostasis of various tissues, including erythropoietic islands of the BM^17^ and aged BM-resident macrophages were shown to expand platelet-biased haematopoietic stem cells (HSCs)^18^. We found that approximately 12% of mature MKs colocalized with CD68^+^ macrophages in the steady state (Extended Data Fig. 4a). These macrophage–MK contacts did not change during immune-mediated thrombocytopenia despite the increase in apoptotic MKs (Extended Data Fig. 4a). Furthermore, depletion of macrophages (through CSF1R inhibition (PLX5622)^19^ or by using *Cd11b-DTR* mice^20^) did not significantly alter MK, MKP and platelet counts (Extended Data Fig. 4b,c), indicating a minimal contribution to megakaryopoiesis. We obtained similar results after depletion of phagocytic neutrophils (Extended Data Fig. 4d).

**Fig. 2:**
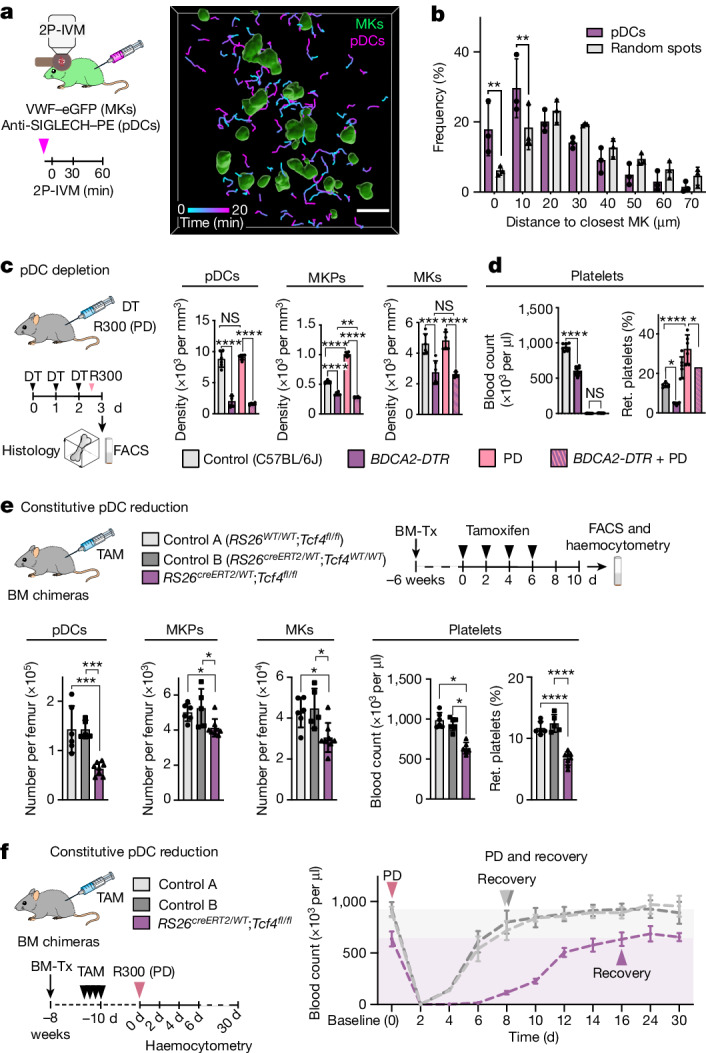
pDCs are BM niche cells that regulate megakaryopoiesis. **a**, 2P-IVM analysis of pDC migration in close proximity to the megakaryocytic lineage. MK/MKPs: VWF–eGFP^+^ (green); pDCs: anti-SIGLECH–PE (2 µg per 25 g intravenously (i.v.) 15 min before imaging) (magenta). **b**, The distribution of pDCs relative to their distance from MKs compared with calculated random spots. *n* = 3 mice. Data are mean ± s.d. Statistical analysis was performed using multiple unpaired *t*-tests with Holm–Šidák test; **P = 0.0068 (0 μm), **P = 0.0093 (10 μm). **c**,**d**, Impaired megakaryopoiesis at steady state and under stress after pDC depletion in *BDCA2-DTR* mice. **c**, Cell numbers were quantified using histology or FACS (see also Extended Data Fig. 5b). *n* = 6 mice. DT, diphtheria toxin. **d**, Platelet counts (haemocytometry) (*n* = 8 mice) and the fraction of reticulated (ret.) platelets (FACS; thiazole orange). *n* = 6 mice. Data are mean ± s.d. Statistical analysis was performed using one-way ANOVA with Tukey’s test; pDCs: ****P = 0.00000000002 (control and *BDCA-DTR*), ****P = 0.000000000003 (PD and *BDCA-DTR* + PD), NS, *P* = 0.89; MKPs: ****P = 0.0000000002 (control and *BDCA-DTR*), ****P = 0.00000000000002 (control and PD), ****P = 0.00000000000002 (PD and *BDCA-DTR* + PD), **P = 0.0068; MKs: ***P = 0.00012, ****P = 0.000014, NS, *P* = 0.98; platelets: ****P = 0.000000000000001 (control and *BDCA-DTR*), NS, *P* = 0.997; reticulated platelets: *P = 0.0102 (control and *BDCA-DTR*), *P = 0.0105 (PD and *BDCA-DTR* + PD) and ****P = 0.000005. **e**, Mice with constitutively reduced pDC numbers show altered megakaryopoiesis. *n* = 6 (control A (*RS26*^*WT/WT*^*;Tcf4*^*fl/fl*^ BM chimera)), *n* = 5 (control B (*RS26*^*creERT2/WT*^*;Tcf4*^*WT/WT*^ BM chimera)) and *n* = 8 (*Tcf4*^*−/−*^(*RS26*^*creERT2/WT*^*;Tcf4*^*fl/fl*^ BM chimera)) mice. Data are mean ± s.d. Statistical analysis was performed using one-way ANOVA with Holm–Šidák test; pDCs: ***P = 0.00024; MKPs: *P = 0.033 (*RS26*^*creERT2/WT*^*;Tcf4*^*fl/fl*^ and control A), *P = 0.025 (*RS26*^*creERT2/WT*^*;Tcf4*^*fl/fl*^ and control B); MKs: *P = 0.0124; platelets: *P = 0.0228; reticulated platelets: ****P = 0.00000068 (*RS26*^*creERT2/WT*^;*Tcf4*^*fl/fl*^ and control A), ****P = 0.00000045 (*RS26*^*creERT2/WT*^*;Tcf4*^*fl/fl*^ and control B). TAM, tamoxifen. **f**, Delayed recovery after PD in *Tcf4*^*−/−*^ BM chimeras. *n* = 6 mice. Data are mean ± s.d. Statistical analysis was performed using two-way ANOVA with Tukey’s test, showing a significant delay in recovery: day 0 versus day 8: *P* = 0.084 (control A), *P* = 0.22 (control B), *P* = 0.0000398 (*RS26*^*creERT2/WT*^;*Tcf4*^*fl/fl*^). Scale bar, 50 µm (**a**). Source data

pDCs are another subset of innate immune cells that are specialized in detecting apoptotic cells and nuclei acids^21,22^. Although pDCs are rare in peripheral tissues, they are abundant in the BM, where they originate^23^. pDCs migrate in the BM with mean speeds of around 4 µm min^−1^ and without any clear directionality (Fig. 2a, Extended Data Fig. 4e–g and Supplementary Video 3). Compared with simulations of random localizations, pDCs showed an increased probability of residing in close proximity (<10 µm) to MKs (Fig. 2b). This distance to MKs was maintained in situations with increased MK turnover (Extended Data Fig. 4h). Approximately 15% of mature MKs colocalized with BST2^+^ pDCs during steady state (Extended Data Fig. 4i) and these co-localizations increased by twofold in response to PD (Extended Data Fig. 4i). The total number of pDCs in the BM remained unaffected by PD, suggesting specific rather than stochastic recruitment to the megakaryocytic niche (Extended Data Fig. 4i).

**Extended Data Fig. 5:**
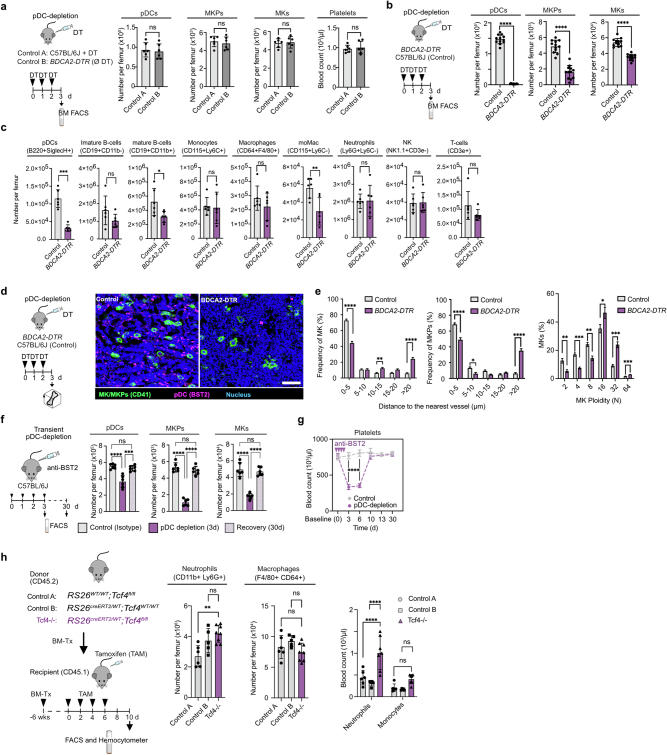
pDCs control megakaryopoiesis. **a-d**, Impaired megakaryopoiesis following pDC-depletion in *BDCA2-DTR* mice. **a**, DT treatment does not affect megakaryopoiesis. *C57BL/6* *J* mice treated with DT (Control A) were compared to untreated *BDCA2-DTR* mice (Control B). Cell numbers were quantified by FACS; n = 6 mice; Mean ± SD; unpaired t-test Welch’s correction; ns: no statistical significance. **b**, Cell numbers were quantified by FACS; n = 12 mice; Mean ± SD; unpaired t-test Welch’s correction, ****(MKs and MKPs): p = 0.000000001, ****(pDCs): p = 0.000000000001. **c**, Cell numbers were quantified by FACS; n = 6 mice; Mean ± SD; unpaired t-test Welch’s correction, ***: p = 0.0002, **: p = 0.0083, *: p = 0.048, ns: no statistical significance. **d**, Representative confocal micrograph of BM; MKs (green, CD41); pDCs (magenta, BST2^+^); nuclei (blue, Hoechst). Scale bars = 100 µm. **e**, pDC-depletion disrupts the megakaryocytic niche and alters the distribution of MKPs and MKs within the BM. BM whole-mounts were stained for MKs (CD41^+^CD42^+^) and MKP (CD41^+^CD42^−^) and positioning was quantified in relation to blood vessels (CD144^+^). Frequency of MKs and MKPs in close contact to blood vessels significantly decreased following pDC-depletion; n = 12 mice; multiple unpaired t-test/Holm-Sidak, ****(MK(0-5): p = 0.00000000001, **(MK(10-15): p = 0.003, ****(MK( > 20): p = 0.000000001, ****(MKP(0-5): p = 0.000005, *(MKP(5-10): p = 0.01, ****(MK( > 0): p = 0.00000000005; Mean ± SD. Notably, the percentage of high ploidy and therefore large MKs increased after pDC depletion, suggesting that misguided positioning rather reduced MK size underlie the increased distance to the vasculature; n = 3 mice; multiple unpaired t-tests/Holm-Sidak; **(2):p = 0.003, ***(4): p = 0.0003, **(8): p = 0.002, *(16): p = 0.017, ***(32): p = 0.0003, ***(64): p = 0.0007; Mean ± SD. **f**, Transient pDC depletion and recovery after anti-BST2 treatment (n = 6 mice); Mean ± SD; one-way ANOVA/Tukey; pDCs: ***: p = 0.0001, ****: p = 0.00003, MKPs: ****: p = 0.00000000008 and p = 0.000000003, MKs: ****: p = 0.0000003 and p = 0.0000008. **g**, Thrombocytopenia and recovery after transient pDC-depletion (n = 6); Mean ± SD; multiple unpaired t-tests/Holm-Sidak; ****: p = 0.000000004 and p = 0.000000001. **h**, Neutrophil, macrophage and monocyte counts in mice with constitutively reduced pDC-numbers (*RS26*^*Cre-ERT2/wt*^*;Tcf4*^*fl/fl*^ BM chimera) (n = 5-7). Mean ± SD; BM: one-way ANOVA/Holm-Sidak; **: p = 0.016; Blood: 2way ANOVA/Holm-Sidak, ****: p = 0.00001 and p = 0.000002. Source data

To investigate whether pDCs are essential to control megakaryopoiesis and MK homeostasis in vivo, we depleted pDCs by treating mice expressing the diphtheria toxin receptor under the *Clec4c* promoter (hereafter *BDCA2-DTR* mice) with diphtheria toxin^24^. After 3 days of treatment, about 80% of pDCs (BST2^+^SIGLECH^+^B220^+^) were cleared from the BM (Fig. 2c and Extended Data Fig. 5a–d). Analysis of the megakaryocytic lineage using fluorescence-activated cell sorting (FACS) and whole-mount immunostaining analysis revealed severely impaired megakaryopoiesis in response to pDC depletion with a substantial decrease in MKP (50%) and MK (25%) numbers (Fig. 2c and Extended Data Fig. 5b,c). The positioning of MKs and MKPs within the BM compartment was altered compared with control mice, indicating a crucial role of pDCs in maintaining the megakaryocytic niche (Extended Data Fig. 5e). Defective MK homeostasis in pDC-ablated mice was associated with reduced platelet production, as indicated by a twofold decrease in the reticulated (young) platelet fraction, and a 40% drop in total circulating platelet counts at steady state (Fig. 2d). Notably, the sharp increase in megakaryopoiesis induced by thrombocytopenia was completely blocked by pDC depletion, indicating their critical role in both steady-state and stress situations (Fig. 2c,d). Moreover, we obtained similar results after depletion of pDCs by antibodies (anti-BST2; clone 927)^25^ (Extended Data Fig. 5f,g) and in mice with constitutively reduced pDC numbers (*RS26*^*creERT2/WT*^*;Tcf4*^*fl/fl*^)^26^ (Fig. 2e and Extended Data Fig. 5h).

To characterize the precise kinetics of pDC-regulated megakaryopoiesis and platelet production, we transiently depleted pDCs and monitored the peripheral platelet count. Three days after the first injection of the pDC-depleting antibody, when the numbers of BM pDCs have efficiently dropped, platelet counts decreased by approximately 40% (Extended Data Fig. 5g). After the last injection of pDC-depleting antibodies (day 3), platelet counts remained diminished for another 3 days before returning to the baseline (Extended Data Fig. 5g), which was paralleled by normalization of pDC numbers, as well as MK and MKP numbers in the BM (Extended Data Fig. 5f). Thus, these data show that pharmacological alteration of pDC-driven megakaryopoiesis has immediate and reversible consequences on the circulating platelet pool. We assessed how mice with constitutively reduced pDC numbers cope with acute platelet demand. We depleted the entire circulating platelet pool in control and *RS26*^*creERT2/WT*^*;Tcf4*^*fl/fl*^ mice and continuously monitored its recovery (Fig. 2f). While control mice recovered to the baseline within 8 days, platelet recovery in *RS26*^*creERT2/WT*^*;Tcf4*^*fl/fl*^ mice was substantially delayed by more than a week (Fig. 2f), highlighting that pDCs are indispensable for on-demand platelet production.

Taken together, our data show that pDCs stimulate megakaryopoiesis to ensure MK and platelet homeostasis at steady state and during stress.

## pDCs sense MK-derived extracellular DNA

**Fig. 3:**
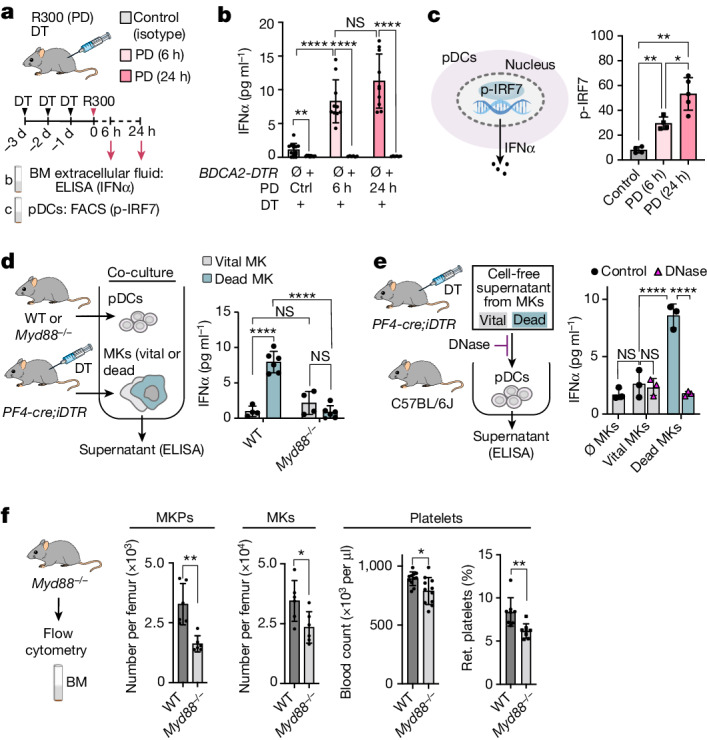
Innate immune sensing drives pDC activation in response to MK-derived extracellular DNA. **a**,**b**, IFNα levels in the BM are pDC dependent at steady state and under stress. **a**, The experimental schematic. **b**, IFNα levels in the BM are pDC dependent, as determined using ELISA. *BDCA2-DTR-neg* (C57BL/6J): *n* = 12 (control) and *n* = 10 (PD (6 h) and PD (24 h)) mice; *BDCA2-DTR-pos*: *n* = 6 (control, PD (6 h) and PD (24 h)) mice. Data are mean ± s.d. Statistical analysis was performed using unpaired *t*-tests with Welch’s correction; **P = 0.0029 (control (*BDCA-DTR-neg* versus *BDCA-DTR-pos*)), ****P = 0.00002 (PD 6 h (*BDCA-DTR-neg* versus *BDCA-DTR-pos*)), ****P = 0.00001 (PD 24 h (*BDCA2-DTR-neg* versus *BDCA2-DTR-pos*)), ****P = 0.00004 (*BDCA2-DTR-neg* (control versus PD 6 h)), NS, *P* = 0.0812 (*BDCA2-DTR-neg* (PD 6 h versus PD 24 h)). **c**, Elevated p-IRF7 in pDCs after PD as determined using FACS. *n* = 4 (control and PD (6 h)) and *n* = 6 (PD (24 h)) mice. Data are mean ± s.d. Statistical analysis was performed using Brown–Forsythe ANOVA with Dunnett’s test; *P = 0.036, **P = 0.004. **d**, Co-culture of BM-derived pDCs (WT and *Myd88*^*−/−*^) and MKs. MK cell death was induced by DT injection in *PF4-cre;RS26-iDTR* mice. *n* = 6; PBS-injected mice were used as controls (*n* = 4). After 18 h of co-culture, IFNα was measured in the supernatants using ELISA. Data are mean ± s.d. Statistical analysis was performed using two-way ANOVA with Tukey’s test; ****P = 0.00000001 (WT pDCs (vital MKs versus dead MKs)), NS, *P* = 0.406 (*Myd88*^*−/−*^ pDCs (vital MKs versus dead MKs)), *P* = 0.559 (vital MKs (WT pDCs versus *Myd88*^*−/−*^ pDCs)), ****P = 0.0000002 (dead MKs (WT pDCs versus *Myd88*^*−/−*^ pDCs)). **e**, MK-derived cell-free DNA activates pDCs. IFNα was measured using ELISA 30 min after incubation with MK supernatants. *n* = 3. Data are mean ± s.d. Statistical analysis was performed using one-way ANOVA with Tukey’s test; NS, *P* = 0.708 (no MKs versus vital MKs), *P* = 0.995 (vital MKs versus vital MKs + DNase), ****P = 0.00003 (vital MKs versus dead MKs), ****P = 0.00001 (dead MKs versus dead MKs + DNase). **f**, *Myd88*^*−/−*^ mice show impaired megakaryopoiesis. *n* = 6 (MKPs/MKs), *n* = 11 (platelets) and *n* = 8 (reticulated platelets) mice. Data are mean ± s.d. Statistical analysis was performed using unpaired *t*-tests with Welch’s correction; **P = 0.0038 (MKPs), *P = 0.033 (MKs), *P = 0.016 (platelets), **P = 0.0068 (reticulated platelets). Source data

**Extended Data Fig. 6:**
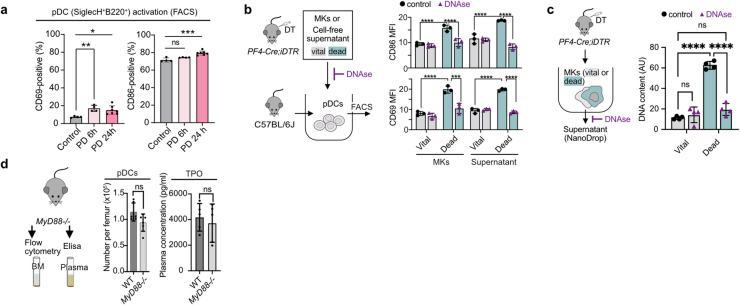
Innate immune sensing drives pDC activation in response to MK-derived extracellular DNA. **a**, FACS analysis of activation markers of BM pDCs following PD (pink: 6 h; red: 24 h). Ctrl/PD6h: n = 4 mice, PD24h: n = 7; one-way ANOVA/Dunnnett; CD69-postive: **: p = 0.0072 and *: p = 0.015, CD86-positive ***: p = 0.0001, ns=0.124; Mean ± SD. **b**, FACS analysis of activation markers of BM pDCs co-cultured with vital or apoptotic MKs or supernatants and in the presence or absence of DNAseI (n = 3 experiments). Mean ± SD; 2way ANOVA/Tukey; CD86: ****(vital MK vs. dead MK): p = 0.0000003, ****(dead MK vs. dead MK + DNAse): p = 0.000008, ****(vital supernatant vs. dead supernatant): p = 0.000001, ****(dead supernatant vs. dead supernatant + DNAse): p = 0.0000006; CD69: ****(vital MK vs. dead MK): p = 0.00009, ***(dead MK vs. dead MK + DNAse): p = 0.0003, ****(vital supernatant vs. dead supernatant): p = 0.00002, ****(dead supernatant vs. dead supernatant + DNAse): p = 0.0000001. **c**, MK supernatants contain cell-free DNA (n = 4 experiments). Mean ± SD; one-way ANOVA/Tukey; ****(vital vs. dead): p = 0.00000005, ****(dead vs. dead+DNAse): p = 0.0000003. **d**, MyD88−/− mice have normal pDC counts in BM (n = 6 mice; FACS) and normal plasma TPO levels (n = 5 mice; ELISA). Mean ± SD; unpaired t test; ns: no statistical significance. Source data

pDCs encountering apoptotic cells become activated and release IFNα into their microenvironment in an interferon regulatory factor 7 (IRF7)-dependent manner^23^. Depletion of pDCs significantly reduced IFNα levels in BM lavages, indicating that pDCs are a major source of IFNα in the BM at steady state (Fig. 3a,b). The numbers of apoptotic MKs increases in response to high platelet demand (Fig. 1l). This was accompanied by increased activation of pDCs and phosphorylation of IRF7 (p-IRF7) (Fig. 3c and Extended Data Fig. 6a) as well as a considerable increase in IFNα levels in the BM extracellular fluid, which was absent in pDC-depleted mice (Fig. 3b). Accordingly, pDCs that were co-cultured with apoptotic MKs in vitro released high amounts of IFNα, while IFNα was barely detectable in co-cultures with vital MKs (Fig. 3d). Incubation of pDCs with cell-free supernatant from apoptotic MKs was sufficient for robust activation, suggesting that physical cell–cell contact is not required for pDC activation (Fig. 3e and Extended Data Fig. 6b). Apoptotic MKs are a rich source of cell-free DNA^27^ (Extended Data Fig. 6c), which is a potent activator of pDCs through the TLR9–MYD88 pathway leading to IRF7 activation and type I interferon production^23^. The presence of DNase efficiently blocked activation of pDCs (Fig. 3e and Extended Data Fig. 6b), and MYD88-deficient pDCs showed impaired release of IFNα in response to supernatants of apoptotic MKs (Fig. 3d). Similar to pDC-depleted mice, MYD88-deficient animals exhibited reduced MKP, MK and platelet counts, while both pDC numbers and TPO levels were not significantly altered in these mice (Fig. 3f and Extended Data Fig. 6d). Collectively, these data indicate that pDCs sense and respond to MK-derived cell-free DNA through MYD88–IRF7 signalling and IFNα release, which is critical for homeostasis of the megakaryocytic lineage.

## IFNα drives pDC-dependent megakaryopoiesis

**Fig. 4:**
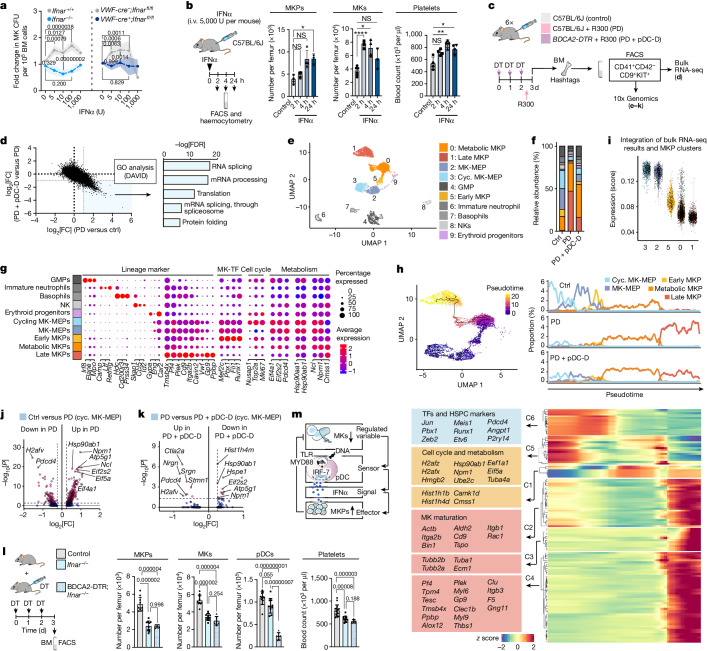
pDC-dependent IFNα drives megakaryopoiesis. **a**, MK colony-forming unit (CFU) assay after TPO (50 ng ml^−1^) and IFNα (as indicated) treatment. Conditional deletion in MKPs (*Vwf-cre;Ifnar*^*fl/fl*^; *n* = 6 mice) and global deletion (*Ifnar*^*−/−*^; *n* = 5 mice) confirmed a direct and IFNAR-dependent role of IFNα. Data are mean ± s.d. Statistical analysis was performed using two-way ANOVA with Šidák’s test; *P* values are shown. **b**, Increased megakaryopoiesis after IFNα treatment. MKs: *n* = 6 (control), *n* = 4 (2 h and 4 h), *n* = 3 (24 h); MKPs: *n* = 4 (control, 2 h and 4 h), *n* = 3 (24 h); and platelets: *n* = 4 (control, 2 h, 4 h and 24 h) mice. Data are mean ± s.d. Statistical analysis was performed using Brown–Forsythe ANOVA with Dunnett’s test; *P = 0.0198 (MKPs); *P = 0.029 (MKs), ****P = 0.00003 (MKs); *P = 0.031 (platelets), **P = 0.0088 (platelets). **c**, The experimental design of the RNA-seq experiments. **d**, Metabolic activation of MKPs in bulk RNA-seq (CD41^+^CD42^−^CD9^+^KIT^+^). The scatter plot shows deregulated genes (log_2_[FC]) in PD versus control and PD + pDC depletion (pDC-D) versus PD, plotted against each other. GO analysis revealed upregulated genes (false-discovery rate (FDR) < 0.05) associated with terms for transcription and translation (top five terms). **e**, UMAP plot of scRNA-seq data (sorted CD41^+^CD42^−^CD9^+^KIT^+^ progenitors). GMP, granulocyte–monocyte progenitor. **f**, The frequency of each cell type and condition. **g**, Annotation by canonical gene expression markers. **h**, Trajectory analysis of MKP clusters. Top left, pseudotemporal ordering (Monocle3) of MKPs superimposed onto UMAP clusters (colour coded on the basis of progression in pseudotime). Top right, the proportion of MKP subsets for each condition along pseudotime. Bottom, heat map of genes associated with pseudotime (*q* < 0.01) clustered by pseudotemporal expression pattern. Selected genes are shown for each cluster (1–6) (the full list is provided in Supplementary Table 1). **i**, Genes upregulated after PD and downregulated after PD + pDC depletion defined from bulk RNA-seq analysis were summarized into a gene score (average expression across the gene set) and visualized by MKP clusters (scRNA-seq). **j**,**k**, Differentially expressed genes (Wilcoxon test). The horizontal dashed line indicates *P* = 0.05. The vertical dashed line indicates log_2_[FC] = 0.25; red, *P* < 0.05 and log_2_[FC] > 0.25; blue, *P* ≧ 0.05 and log_2_[FC] > 0.25. **l**, Decreased megakaryopoiesis in *Ifnar*^*−/−*^ mice. pDC depletion in *BDCA2-DTR;Ifnar*^*−/−*^ mice had no additive effect. MKs, MKPs and pDCs: *n* = 10 (control and *Ifnar*^*−/−*^) and *n* = 6 (*BDCA2-DTR;Ifnar*^*−/−*^) mice; platelets: *n* = 16 (control), *n* = 11 (*Ifnar*^*−/−*^) and *n* = 6 (*BDCA2-DTR;Ifnar*^*−/−*^) mice. Data are mean ± s.d. Statistical analysis was performed using Brown–Forsythe ANOVA with Dunnett’s test; *P* values are shown. **m**, Graphical summary. Source data

**Extended Data Fig. 7:**
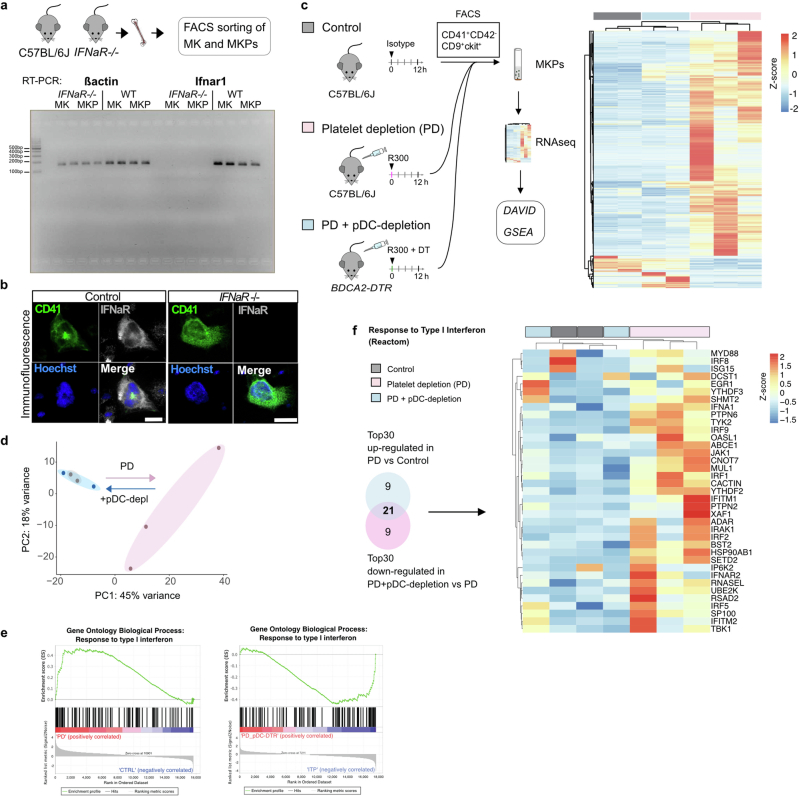
Bulk RNA-seq of MK-primed progenitors. **a**, Expression (mRNA) of *IFNaR* was analysed in sorted MKs and MKPs by RT-PCR. MKPs and MKs from *IFNaR*−/− mice served as negative control; *ß-actin* (housekeeping gene); n = 2. **b**, Representative immunofluorescence staining confirmed expression of IFNaR in isolated MKPs and MKs. *IFNaR−/−* (negative control) (n = 3). Scale bar=10 µm. **c**, Left: Schematic of experimental design. MKPs were sorted from BM of mice treated with isotype antibody (grey; n = 2), R300 (PD, purple; n = 3) or R300 plus DT (PD plus pDC depletion, green; n = 2). Right: Expression heatmap of MKP RNA-Seq data. Heatmap shows MKP genes de-regulated (log2FC < 1 or >1 with FDR < 0.05) in either PD versus control or PD vs. platelet depletion with additional pDC depletion. Heatmap was generated using non-hierarchical clustering on rows and columns using ClustVis R package. **d**, Principal component analysis (PCA) of MKP RNA-Seq data. MKPs exhibit a strong variance shift following PD (grey to magenta). This variance is abrogated when animals were additionally depleted for pDCs (magenta to blue). **e**, Gene set enrichment analysis was performed on RNA-seq data (Reactom). “Response to type I interferon” is shown. PD compared to control (left) and PD with additional pDC depletion (right). **f**, Left: intersection analysis (Venn diagram) confirms a high overlap of type I interferon response genes to be inversely regulated between conditions (21 genes of Top 30). Heatmap show unsupervised clustering of differentially regulated genes (unique genes of both Top30 lists). Source data

pDCs were required to maintain basal IFNα levels of the BM extracellular fluid at steady state and to increase levels during stress (Fig. 3a–c). Cells of the MK lineage expressed the IFNα receptor (IFNAR) (Extended Data Fig. 7a,b) and IFNα and TPO synergistically boosted megakaryopoiesis in an IFNAR-dependent manner in vitro (Fig. 4a). Moreover, systemic treatment of mice with IFNα induced rapid and immediate megakaryopoiesis in vivo—doubling MK numbers within 2 h and MKP numbers within 4 h—and an increase in circulating platelets by 1.5-fold (Fig. 4b). IFNα was previously reported to fuel megakaryopoiesis by upregulating cell cycle and translational activity of MK-primed stem and progenitor cells in the BM^28,29^. Accordingly, bulk RNA-sequencing (RNA-seq) analyses of MK-primed progenitors (CD41^+^CD42^−^CD9^+^KIT^+^) in thrombocytopenic mice revealed pDC-dependent enrichment of genes associated with cell division and translation, as well as moderate induction of interferon-response genes, consistent with previous reports^28^ (Fig. 4c,d and Extended Data Fig. 7c–f).

**Extended Data Fig. 8:**
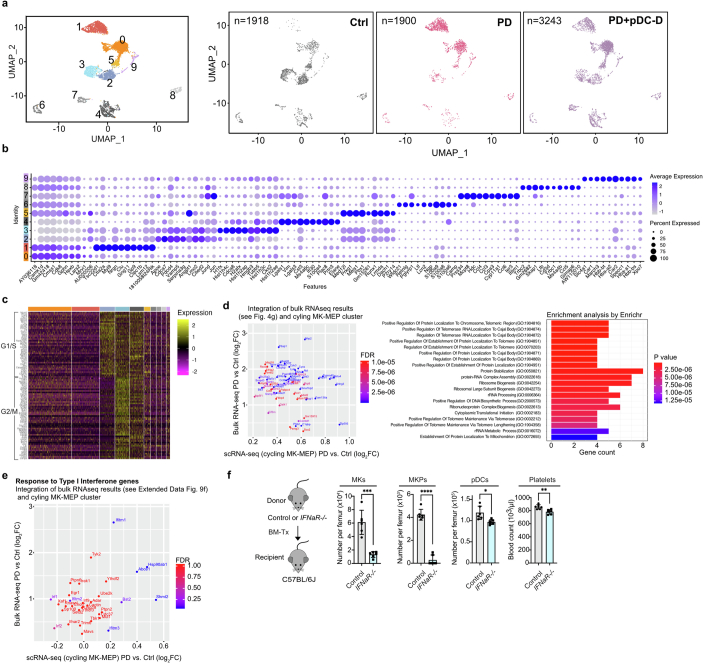
scRNAseq of MK-primed progenitors. **a**, UMAP plot of Control, platelet depletion (PD) and PD+pDC-depletion. Colours indicate the cluster assignment. **b**, Dot plot of cluster defining genes; Top 10 differentially expressed genes are shown. Identity and colours indicate the clusters shown in panel a. **c**, Heat map shows cell cycle genes^84^. The colour bar on the top x-axis indicates the clusters shown in panel a. **d**, Integration of bulk RNAseq data and scRNAseq data. Genes upregulated in response to PD both in bulk and cycling MK-MEPs are shown. Right: Enrichment analysis of identified process associated with increased metabolic activity. **e**, Integration of bulk RNAseq data and scRNAseq data. Response to type I interferon genes upregulated in response to PD either in bulk and cycling MK-MEPs are shown. **f**, Decreased MKP and MK numbers in BM chimeric *IFNaR−/−* mice. Irradiated WT mice received BM from *IFNaR−/−* or *IFNaR* +/+ *(Control)* donors, respectively. Mice were subjected to analysis 8 weeks after transplantation; Mean ± SD; unpaired t-test/Welch’s correction; ****: p = 0.00000000361, ***: p = 0.0010, **: p = 0.0035, *: p = 0.0170. Source data

To further examine the molecular signature of MK-primed progenitors responding to increased platelet demand and to characterize their genomic states, we sorted CD41^+^CD42^−^CD9^+^KIT^+^ BM cells from wild-type and thrombocytopenic mice and analysed their transcriptome at the single-cell level (Fig. 4c–f and Extended Data Fig. 8a). We identified nine transcriptionally distinct clusters of progenitors, five of which were MK primed and expressed MK marker genes (*Pf4*, *Itga2b*, *Vwf*) and transcription factors (*Fli1*, *Pbx1*, *Mef2c*, *Runx1*), albeit to varying degrees^30–32^ (Fig. 4g and Extended Data Fig. 8b and Supplementary table 1). We annotated MK-primed progenitors on the basis of their differentially expressed marker genes and enriched Gene Ontology (GO) into cycling and non-cycling MK primed megakaryocyte–erythrocyte progenitors (MK-MEPs)^33^ (*Eng*, *Car2*), early MKPs (*Pbx1*, *Mef2c*, *Fli1*) and late MKPs (*Gp9*, *Ppbp*) as well as metabolically active MKPs expressing high levels of genes involved in ribosome biogenesis (*Ncl*, *Npm1*), translation initiation (*Eif4a1*, *Eif2s2*) and protein chaperones (*Hsp90*) (Fig. 4g and Extended Data Fig. 8b,c). Pseudotime analysis revealed that MKP subsets aligned along a distinct developmental trajectory consistent with the expression of maturation stage-dependent marker genes of megakaryopoiesis (Fig. 4h). We next investigated whether increased platelet demand affected MKP developmental stages. Indeed, the number and proportion of metabolically active and late-stage MKPs increased significantly at the expense of MK-MEPs and early MKPs, indicating PD-induced differentiation of MK-primed progenitors^34^ (Fig. 4f,h). The shift toward more mature MKPs was attenuated in pDC-depleted mice (Fig. 4f,h), consistent with pDCs supporting efficient cell cycle induction and protein synthesis of MK-primed progenitor cells (Fig. 4d). Integration of bulk RNA-seq results and scRNA-seq clusters revealed that, among MK-primed progenitors, cycling MK-MEPs showed the highest expression of genes responsive to PD and regulated by pDCs (Fig. 4i and Extended Data Fig. 8c–e). Accordingly, differentially upregulated genes at the single-cell level were enriched in ribosome biogenesis (for example, *Npm1*, *Ncl*) and initiation of translation (such as *Eif2s2*, *Eif4a*) (Fig. 4j), suggesting increased metabolic activity of cycling MK-MEPs in response to PD, which was attenuated after pDC-depletion (Fig. 4k). Among the highest differentially downregulated genes in response to PD was *Pdcd4*, a translation inhibitor that was previously reported to have a role in cell growth^35^ and emergency megakaryopoiesis^29^ and to be regulated by IFNα signalling^36^. Indeed, depletion pDCs in thrombocytopenic mice resulted in significantly increased expression of *Pdcd4* in cycling MK-MEPs (Fig. 4j,k). Thus, our data suggest that pDCs drive megakaryopoiesis by initiating protein translation in early MK-primed progenitors^29^, consistent with their role as major carriers of IFNα.

pDCs are a major source of IFNα in unperturbed BM (Fig. 3b), suggesting a role for pDC-derived IFNα also in steady-state MK homeostasis. Similar to pDC-depletion, disruption of IFNα signalling in IFNAR-deficient mice reduced MKP numbers at steady state and altered MK and platelet homeostasis (Fig. 4l). *Ifnar*^*−/−*^ BM chimeras phenocopied global *Ifnar* deletion (Extended Data Fig. 8f). Ablation of pDCs in *Ifnar*^*−/−*^ mice (*BDCA2-DTR;Ifnar*^*−/−*^ mice) had no additive effect on either MKP, MK or platelet numbers (Fig. 4l), indicating that IFNα is the major mediator in pDC-regulated megakaryopoiesis.

Taken together, our data suggest that pDCs encountering apoptotic MKs release IFNα into the BM niche, which in turn fuels expansion and maturation of MK-primed progenitors through IFNAR signalling to maintain MK homeostasis at steady state and under stress (Fig. 4m).

## Increased pDC–MK contacts in patients with ITP

**Fig. 5:**
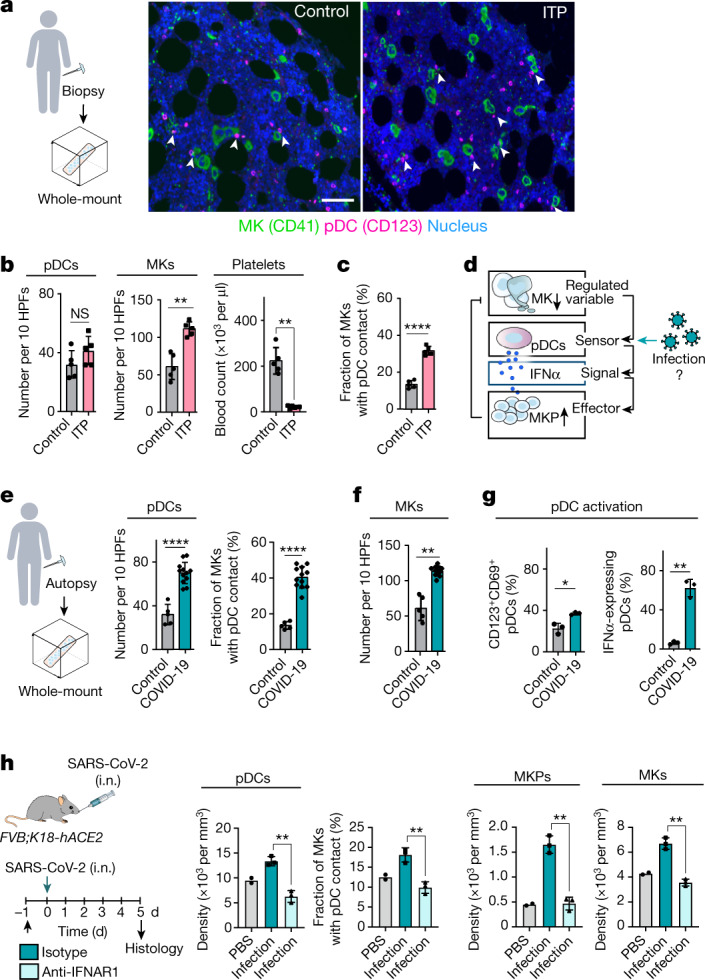
Infection alters pDC-regulated MK homeostasis in humans and mice. **a**, Immunohistology of human BM biopsies from healthy controls and patients with secondary ITP with non-Hodgkin lymphoma (without BM involvement). MKs (CD41^+^, >15 μm; green), pDCs (CD123^+^; magenta), nuclei (DAPI; blue). Scale bar, 50 µm. **b**,**c**, Quantification of the number of pDCs, MKs per high power field (HPF) size of 0.9 mm × 0.7 mm and platelets (**b**) and the fraction of MKs with pDC contact (**c**) from the experiment in **a**. *n* = 5 patients. Data are mean ± s.d. Statistical analysis was performed using unpaired *t*-tests with Welch’s correction; NS, *P* = 0.158 (pDCs), **P = 0.0011 (MKs), **P = 0.0014 (platelets), ****P = 0.000002 (MKs/pDCs). **d**, Infection may alter the role of pDCs as homeostatic sensors. **e**,**f**, Immunohistology of human BM biopsies from healthy control patients (the same patients as shown in **a** and **b**) and from autopsies of patients with COVID-19 (see also Extended Data Fig. 9d). Quantification of the number of pDCs and the fraction of MKs in contact with pDCs (**e**) and the number of MKs (**f**) is shown. *n* = 5 (control) and *n* = 12 (COVID-19) individuals. Data are mean ± s.d. Statistical analysis was performed using unpaired *t*-tests with Welch’s correction; ****P = 0.00007 (pDCs), ****P = 0.0000000007 (MKs/pDCs), **P = 0.0018 (MKs). **g**, Increased activation of pDCs in the BM of patients with COVID-19. Quantification of activation marker CD69 (left) and IFNα expression (right) (Immunohistology; see also Extended Data Fig. 9e). *n* = 3 patients. Data are mean ± s.d. Statistical analysis was performed using unpaired *t*-tests with Welch’s correction; *P = 0.0304, **P = 0.0069. **h**, BM from *FVB;K18-hACE2* mice infected with SARS CoV-2 (10^5^ median tissue culture infectious dose (TCID_50_) SARS-CoV-2 per mouse in 25 μl intranasally (i.n.)) were analysed in the presence (*n* = 3) or absence (*n* = 3) of IFNAR1 blocking antibody and compared to untreated control mice (PBS, *n* = 2) (immunohistology). Data are mean ± s.d. Statistical analysis was performed using unpaired *t*-tests with Welch’s correction; **P = 0.0015 (pDCs), **P = 0.0041 (percentage of MK–pDC-contacts), **P = 0.0011 (MKPs), **P = 0.0014 (MKs). Source data

To define whether pDC–MK contacts are present in humans, we analysed BM sections from healthy individuals (Fig. 5a,b). Consistent with our findings in mice, approximately 12% of MKs co-localized with pDCs under steady state (Fig. 5c). We then examined the BM of a cohort of patients with severe immune thrombocytopenic purpura (ITP) (secondary ITP with non-Hodgkin lymphoma without BM involvement; patient characteristics are provided in Supplementary Table 2). Similar to PD in mice, the number of circulating platelets was severely reduced in patients with ITP (Fig. 5b). pDC–MK contacts were threefold higher in ITP compared with in control patients (Fig. 5c) while the number of MKs doubled (Fig. 5b). This suggests that pDCs also act as sentinels of MK turnover in human BM.

## Infection alters MK homeostasis

**Extended Data Fig. 9:**
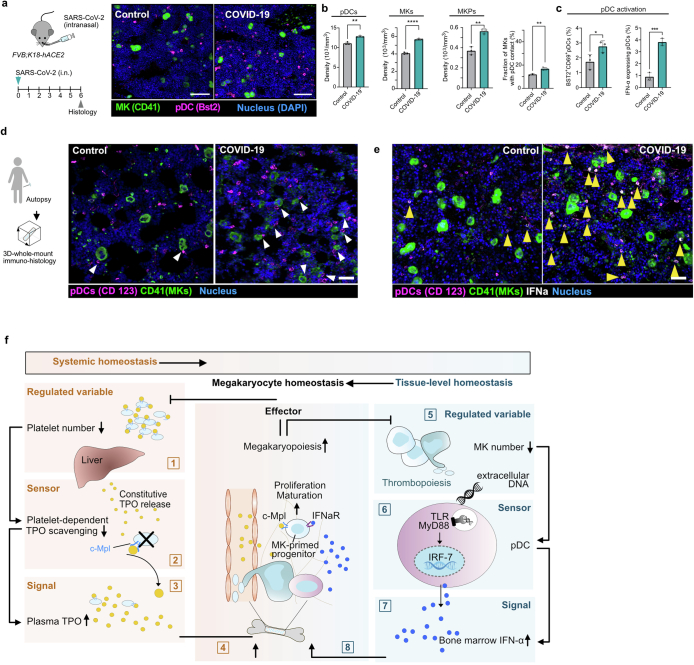
pDC-driven megakaryopoiesis is altered in infection. **a**, Augmented megakaryopoiesis in a humanized mouse model of SARS-CoV-2 infection. BM from *FVB;K18hACE2* mice infected with SARS CoV-2 (10^5^ TCID50 SARS-CoV-2/mouse in 25μl intranasally or untreated control mice were analysed by immunohistology (n = 3). MKs (green; CD41; >15 μm); MKPs (green; CD41; <15 μm); pDCs (magenta; BST2). Scale bar = 100 µm. **b**, n = 3 mice; Mean ± SD; unpaired t-test with Welch’s correction; MK/pDC contact**: p = 0.0081, pDC number: **: p = 0.0037, MK number: ****: p = 0.000049, MKP **: p = 0.0039. **c**, Frequency of CD69^+^ and IFN-α expressing pDCs in BM increases after SARS-CoV-2 infection (immunohistology). Mean ± SD; Unpaired t-test with Welch’s correction; *: p = 0.0481, ***: p = 0.0004. **d**, **e**, Representative confocal micrographs of human BM (see quantification in Fig. 5e–g). d, white arrows indicate pDCs in contact with MKs (control: n = 5, covid-19: n = 12). e, yellow arrows indicate pDCs expressing large amounts of IFN-α (n = 3). Scale bar = 50 µm. **f**, pDCs act as BM niche cells that control tissue homeostasis of MKs, complementing systemic regulation by TPO (Graphical summary). Left: TPO is the most studied regulator of platelet homeostasis. TPO is constitutively released from the liver and is scavenged by c-Mpl, the TPO receptor expressed on platelets (see 1). Consequently, a decrease in the number of circulating platelets (see 2) is inherently associated with an increase in plasma TPO levels (see 3), which activates HSCs and MK-primed progenitors in the BM via c-Mpl to drive megakaryopoiesis to meet platelet demand (see 4). Thus, the TPO-dependent homeostatic circuit regulating megakaryopoiesis involves circulating platelets as sensors operating at the systemic level. Right: Mature MKs release extracellular DNA (see 5), which is sensed by pDCs via TLRs (see 6) and leads to the release of IFN-α (see 7). IFN-α drives the proliferation and maturation of HSCs and MK-primed progenitors to replenish megakaryocytes in their BM niche (see 8), preventing MK exhaustion and ensuring continuous platelet production. The pDC-dependent homeostatic circuit thus involves innate immune sensing of apoptotic MKs and operates at the tissue level. Loss of pDC-dependent MK homeostasis at the tissue level cannot be compensated for by TPO-dependent MK homeostasis at the systemic level, indicating a non-redundant role of both pathways in regulating megakaryopoiesis. Source data

pDCs are specialized to sense viral infections and are the major source of IFNα in antiviral immunity^37,38^. We hypothesized that, in the emergency of acute infection, viral-triggered activation of pDC and the associated release of IFNα may exceed the homeostatic range required for megakaryopoiesis. This could explain hyperplasia of MKs associated with viral infections such as severe acute respiratory syndrome coronavirus 2 (SARS-CoV-2)^39^ (Fig. 5d). To address whether SARS-CoV-2 infection is associated with dysregulation of pDC-driven megakaryopoiesis, we analysed BM of humanized mice susceptible to SARS-CoV-2 (*FVB-K18-hACE2*). Six days after infection, mice showed elevated pDC numbers within the BM (Extended Data Fig. 9a,b). pDCs engaged in close contact with MKs, and both MKP and MK numbers increased compared with in the uninfected controls, suggesting pDC-driven hyperplasia of the MK lineage (Extended Data Fig. 9b). We next analysed human BM from a cohort of patients who died from COVID-19 (Fig. 5e–g; patient characteristics are provided in Supplementary Table 2). Similar to humanized mice, we found a greater than twofold increase in pDC numbers, and a threefold increase in MK–pDC contacts in patients with COVID-19 compared with the control individuals (Fig. 5e), which was associated with MK hyperplasia (Fig. 5f). pDCs respond to SARS-CoV-2 by producing type I interferons^40^. Accordingly, the fraction of activated, CD69^+^ and IFNα-expressing pDCs in the COVID-19 BM was significantly increased compared with in the BM of healthy controls, both in humans and mice (Fig. 5g and Extended Data Fig. 9c,e). To test whether IFNα signalling drives MK hyperplasia, we treated *FVB-K18-hACE2* mice with an IFNAR-blocking antibody before SARS-CoV-2 infection. In contrast to the infected control animals, antibody-treated mice showed reduced pDC–MK contacts in the BM and did not develop hyperplasia of the megakaryocytic lineage (Fig. 5h). These data suggest that the homeostatic circuit of megakaryopoiesis controlled by pDCs can be perturbed by severe systemic infections and that the resulting imbalanced release of IFNα from pDCs contributes to MK alterations in COVID-19.

## Discussion

Type 1 interferons are mainly recognized for their protective role in viral infections^41^. However, numerous physiological processes beyond antiviral defence have been identified to rely on IFNs, including immunomodulation, immunometabolism^42^, cell cycle regulation, cell survival and cell differentiation^42,43^. Within the BM compartment, constitutive IFNα levels are required for maintenance of the HSC niche, but long-term systemic elevation of IFNα levels may cause exhaustion of HSCs^28,44,45^. Moreover, long-term treatment with high-dose IFNα, as well as infections and diseases associated with persistently high type I IFN levels are associated with impaired platelet production and platelet function^29,46–50^. This indicates that IFNα levels must be precisely controlled to preserve homeostasis of the haematopoietic system.

Here we characterize a homeostatic circuit^8^ of BM tissue that maintains stable cellularity of the megakaryocytic lineage through pDC-dependent release of IFNα (Extended Data Fig. 9f). Our data establish patrolling pDCs as homeostatic sensors that monitor MK turnover by detecting MK-derived cell-free DNA. Innate sensing of self-DNA by pDCs occurs through the TLR9–MYD88–IRF7 pathway^23^ and results in the precisely controlled delivery of IFNα to MK progenitors to prevent MK loss by eliciting on-demand megakaryopoiesis. Thus, these data demonstrate a critical role of inflammatory signalling in the control of MK homeostasis at the tissue level, which complements the well-characterized systemic regulation of MK lineage homeostasis through TPO (Extended Data Fig. 9f). Although our data indicate that pDCs have a central role in both constitutive and stress-induced IFNα release within the BM, it is worth noting that current technical constraints hinder definitive confirmation of pDCs as the exclusive source of IFNα in vivo, primarily due to the absence of IFNα-deficient mouse models.

The control of MK numbers by pDCs may have functions beyond platelet homeostasis: the BM microenvironment is functionally compartmentalized by a heterogeneous population of niche cells that provide physical and soluble signals to spatiotemporally organize haematopoiesis^51^. Besides giving birth to platelets, MKs constitute niche cells of haematopoietic origin that regulate HSCs during homeostasis and stress^34,52–54^. Consequently, maintenance of the megakaryocytic HSC niche requires seamless replenishment of consumed, platelet-producing MKs. Here we establish that pDCs are crucial players in the niche orchestrating thrombopoiesis and megakaryopoiesis to maintain MK homeostasis and may therefore also contribute to the maintenance of the megakaryocytic HSC niche^34,52–54^.

Platelets not only prevent blood loss but also counteract infections through interaction with immune cells^2^. To compensate peripheral platelet consumption during acute infections and to maintain homeostasis of the circulating platelet pool, the systemic inflammatory response must initiate emergency megakaryopoiesis^29^. Our results argue for a role of pDCs as homeostatic sensors responsive to inflammatory stimuli, such as viruses. By monitoring the perivascular MK niche, pDCs are strategically positioned to instantaneously detect systemic inflammatory signatures, which may allow them to anticipate the risk of platelet exhaustion and promptly initiate emergency megakaryopoiesis. Thus, pDC-driven megakaryopoiesis has a role beyond steady-state homeostasis and is probably beneficial in any type of acute tissue injury associated with loss of vascular integrity and platelet consumption.

However, it may also be detrimental, when pDC-driven megakaryopoiesis is mismanaged, for example, during severe viral diseases. A case in point is infection with coronavirus SARS-CoV-2, which dysregulates the fine-tuned IFNα production of pDCs^55^. While analysis of peripheral blood of patients with SARS-CoV-2 infection revealed reduced pDC counts with muted IFNα production^55^, we identify an accumulation of activated, IFNα-releasing pDCs in the BM of patients with severe disease progression. pDCs engage in close contact with MKs, which is accompanied by marked megakaryocytic hyperplasia. Although the mechanisms linking MK hyperplasia to disease progression are still unclear, previous studies have shown a correlation with severe courses of COVID-19 in particular^39^. It is therefore tempting to speculate that pharmacological modulation of the pDC-mediated homeostatic circuit may be of benefit to these patients. In conclusion, we identified a role of pDCs in orchestrating MK and blood platelet homeostasis. Targeting pDC-driven megakaryopoiesis offers options to boost or suppress platelet production in different clinical scenarios.

## Methods

### Materials

A list of all reagents and resources with the source and identifier is provided in Supplementary Table 3.

### Mouse strains

C57BL/6J, C57BL/6J (CD45.1)*, PF4-cre* (*C57BL/6-Tg(Pf4-icre)Q3Rsko/J*)^56^, *Rosa26-iDTR*^*flox*^ (*C57BL/6*^*Gt*^*(ROSA)26*^*Sortm1*^*(HBEGF)*^*Awai/J*^)^57^, *Ifnar*^*−/−*^ (*B6.129S2-Ifnar1*^*tm1Agt/Mmjax*^)^58^, *Ifnar1*^*flox*^ (*B6(Cg)-Ifnar1*^*tm1.1Ees*^*/J*)^59^, *RS26-cre*^*ERT2*^ (*B6.129-Gt(ROSA)26Sor*^*tm1(cre/ERT2)Tyj*^*/J*)^60^*,* *Myd88*^*−/−*^(*B6.129P2(SJL)-Myd88*^*tm1.1Defr*^*/J*)^61^*, CD11b-DTR* (*B6.FVB-Tg(ITGAM-HBEGF/EGFP)34Lan/J*)^62^*, LysM-cre (B6.129P2-Lyz2*^*tm1(cre)Ifo*^*/J*)^63^*, Mcl-1fl/fl* (*B6;129-Mcl1*^*tm3Sjk*^*/J*)^64^ and *BDCA2-DTR* (*C57BL/6-Tg(CLEC4C-HBEGF)956*^*Cln/J*^)^24^ mice were purchased from The Jackson Laboratory. *Vwf-cre* mice were generated by W. Aird and were described previously^65^. *Vwf-eGFP* mice were generated by C. Nerlov and described previously^10^. *Tcf4*^*fl/fl*^ *(C57BL/6N-Tcf4*^*tm1c(EUCOMM)Wtsi*^*/WtsiH)*^66^ mice were obtained from Wellcome Sanger Institute and INFRAFRONTTIER/EMMA partner (Vienna) from which the mouse was received. *PF4-cre* mice were crossed with *Rosa26-iDTR* mice to induce MK cell death in vivo *(PF4-cre; RS26-iDTR)*^52^. *PF4-cre;RS26-iDTR* mice were crossed with *Vwf-eGFP* mice to visualize the megakaryocytic lineage after induction of MK cell death. *Vwf-cre* mice were crossed with *IFNαR1*^*fl/fl*^ mice to conditionally delete *Ifnar* in the megakaryocytic lineage. *BDCA2-DTR* and *Ifnar*^*−/−*^ were cross bred to achieve pDC depletion in *Ifnar*^*−/−*^ animals (*BDCA2-DTR;Ifnar*^*−/−*^). *RS26-cre*^*ERT2*^ mice were cross bred with *Tcf4*^*fl/fl*^ *(C57BL/6N-Tcf4*^*tm1c(EUCOMM)Wtsi*^*/WtsiH)* mice to constitutively reduce pDC numbers. *FVB-K18-hACE2* expressing humanized ACE2 were bred in the Iannacone laboratory^67^.

Both male and female mice were used in this study. Unless otherwise stated, mice of the control and experimental group were sex- matched and age-matched (6–12 weeks). Animals were bred and maintained in the animal facilities of the Walter-Brendel Zentrum (Wbex), the Zentrum für Neuropathologie und Prionforschung (ZNP) or the Biomedical center of the LMU Munich, Germany or IRCCS San Raffaele Scientific Institute, Italy or Institute of Science and Technology Austria, Austria. All mice live in standardized conditions in which temperature, humidity and hours of light and darkness are maintained at a constant level all year round. The housing of laboratory mice was in accordance with European and German animal welfare legislations (5.1-231 5682/LMU/BMC/CAM/), Wbex and ZNP. Room temperature and relative humidity ranged from 20 to 22 °C to 45 to 55%. The light cycle was adjusted to a 12 h–12 h light–dark period. Room air was exchanged 11 times per hour and filtered with HEPA-systems. All of the mice were housed in individually ventilated cages (Typ II long, Tecniplast) under specified-pathogen-free conditions. Hygiene monitoring was performed every 3 months based on the recommendations of the FELASA-14 working group. All of the animals had free access to water and food (irradiated, 10 mm pellet; 1314P, Altromin). The cages were equipped with nesting material (5 × 5 cm, Nestlet, Datesand), a red corner house (Tecniplast) and a rodent play tunnel (7.5 × 3.0 cm, Datesand). Soiled bedding (LASbedding, 3–6 mm, PG3, LASvendi) was removed every 7 days. All of the animal experiments were performed in compliance with all relevant ethical regulations for studies involving mice and were approved by the local legislation on protection of animals (Regierung von Oberbayern, Munich; ROB 55.2-1-54-2532-190-2015; ROB 55.2-2532; Vet 02-17-194).

### Mouse anaesthesia

If not stated otherwise, anaesthesia was performed by isoflurane induction, followed by intraperitoneal injection of medetomidine (0.5 mg per kg body weight), midazolam (5 mg per kg body weight) and fentanyl (0.05 mg per kg body weight). Toe pinching reflexes and breathing pattern were used to determine the adequate depth of anaesthesia. Core body temperature was maintained by heating pads, and narcosis was maintained by repetitive injections of 50% of the induction dose, if necessary.

### Human samples

LMU Munich: BM samples of five patients with clinically proven ITP, of five patients with non-Hodgkin lymphoma without BM involvement and 12 patients who died of COVID-19 were analysed. The samples of patients with ITP and lymphoma were archived material, and the COVID-19 specimens were taken during autopsy. Clinical details are provided in Supplementary Table 2. The study was approved by and conducted according to requirements of the ethics committees at the Ludwig Maximilians University of Munich (20-1039). There was no commercial support for this study. University Clinic Aachen: we included 6 consecutive clinical autopsies of patients who were positive for COVID-19 between 9 March 2020 and 5 May 2020 performed at the Institute of Pathology of the University Clinic Aachen. Each patient had a positive clinical SARS-CoV-2 PCR test from upper or lower respiratory tract before autopsy, confirmed by post-mortem PCR with reverse transcription (RT–PCR). Consent to autopsy was obtained by the legal representatives of the deceased patients. The study was approved by the local ethics committee (EK 304/20, EK 119/20 and EK 092/20). BM samples were obtained using an electric autopsy saw (Medezine 5000, Medezine) from the vertebral bodies. The autopsies were performed in two steps according to a modified standard protocol to further increase employee safety and sample acquisition (developed in the frame of the German Registry of COVID-19 autopsies, www.DeRegCOVID.ukaachen.de). The samples were decalcified in formic acid or EDTA before dehydration and embedding in paraffin. Formalin-fixed, paraffin-embedded BM blocks were cut on a microtome at 1–3 µm thickness and decalcified again in EDTA if necessary.

### Drug treatments

DT was purchased from Sigma-Aldrich (322326) and was intraperitoneally injected into *CD11b-DTR* mice as a single dose of 25 ng per g for 2 days and *BDCA2-DTR* and *BDCA2-DTR;Ifnar*^*−/−*^ mice with a dose of 8 ng per g per day for consecutive 3 days. A single dose was injected into *Pf4-cre;iDTRfl/fl* mice 24 h before the experiment. Platelet-depleting antibodies (R300, anti-GPIbα) and isotype control (C301) were purchased from Emfret and used according to the manufacturer’s protocol. pDC-depleting antibodies (ultra-LEAF purified anti-PDCA-1, 927, BioLegend) were injected intraperitoneally for up to 3 consecutive days at a concentration of 150 µg per mouse at day 1 and 100 µg per mouse on the following days. The isotype control (ultra-LEAF purified rat IgG2bk isotype control, RTK4530, BioLegend) was injected accordingly. Type I IFNα was applied by injecting universal IFNα (PBL, assay science) with 5000 U per mouse intraperitoneally in 200 µl PBS. For macrophage ablation, wild-type mice were feed with PLX 5622 chow (D19101002i, AIN-76A), or control chow (D10001i, AIN-76A) from Research Diets, for 7 consecutive days.

### Tamoxifen injection

Cre-recombinase in *RS26*^*creERT2/WT*^*;Tcf4*^*fl/fl*^ mice was induced by intraperitoneal injection of tamoxifen (Sigma-Aldrich, 10540-29-1) dissolved in corn oil (Sigma-Aldrich C8267) three times every other day (1 mg per day), and the mice were analysed 10 days after the first administration^26^.

### BM transplantation

Lin^−^Sca-1^+^KIT^+^ (LSK) cells were isolated and sorted from BM of *Ifnar*^*−/−*^, *RS26*^*creERT2/WT*^*;Tcf4*^*fl/fl*^ and control mice (Lin-Pacific Blue (Ter-119, CD3, CD8a, CD45R, CD11b, Ly-6G), Sca-1–PE–Cy7, KIT–APC, all purchased from BioLegend, 1:100). A total of 8 × 10^3^ LSK cells was intravenously injected into lethally irradiated C57BL/6J female mice (CD45.1) (two doses of 6.5 Gy with a time interval of 8 h). The BM of chimeras was analysed 8 weeks after the transplantation.

### Mouse model of SARS-CoV-2 infection

*B6.Cg-Tg(K18-ACE2)*^*2Prlmn/*^ *J* mice (on the C57BL/6 background) were purchased from The Jackson Laboratory and bred against FVB mice to obtain C57BL/6 × FVB F_1_ hybrids. Mice were housed under specific-pathogen-free conditions and heterozygous mice were used at 6–10 weeks of age. All of the experimental animal procedures were approved by the Institutional Animal Committee of the San Raffaele Scientific Institute and all infectious work was performed in designed BSL-3 workspaces. Mice were infected intranasal with 10^5^ TCID_50_ of SARS-CoV-2/human/ITA/Milan-UNIMI-1/2020 (GenBank: MT748758.1) in 25 μl. Then, 5 days after infection, the mice were perfused fixed with 4% PFA and the femurs were embedded in Tissue Tek (also see below). The frozen femurs were cut until the marrow was exposed. The femurs were rinsed with PBS and post-fixed with 4% PFA for 15 min at room temperature. The femurs were washed with PBS and incubated with 10% goat serum for 1–2 h at room temperature. BM was stained with anti-mouse CD41 (for MK/MKP), anti-mouse BST2 (for pDC) and DAPI for nucleus staining. In selected experiments, *K18-hACE2* mice were injected intraperitoneally with 2 mg per mouse of anti-IFNAR1 blocking antibody (BioXcell, BE0241, MAR1-5A3) 1 day before infection. All the COVID-19 mouse infection experiments were approved by the Authorization no 270/2022-PR (6EEAF.228).

### Immunohistology of human BM samples

BM biopsies of five patients with confirmed immune thrombocytopenia and platelet counts <30 × 10^9^ per l were compared with age-matched controls (normal BM biopsies performed for lymphoma staging). Tissue was fixed for 12 h in 4% formalin and embedded in paraffin. For immunohistochemistry, 1.5 µm sections were used. Multiplex immunofluorescence or confocal laser-scanning microscopy imaging were performed after antigen retrieval with epitope retrieval buffer (PerkinElmer). Slides were incubated sequentially for 1 h using the following antibodies: pDCs (anti-human CD123, ab257307, Abcam, 1:100); and MKs (anti-human CD41, ab134131, Abcam, 1:100, or MCA467G Bio-Rad, 1:100) and detection was performed using by TSA-Opal620 (PerkinElmer) and TSA-Opal650 (PerkinElmer). Multispectral imaging was performed using the PerkinElmer Vectra Polaris platform. Images were analysed using HALO (Indica labs) software. Furthermore, the samples were imaged on the LSM 880 confocal microscope using the Airyscan module (Carl Zeiss), Plan-Apo ×20/0.8 or ×63/1.46 objectives and analysed using Zen Blue (v.2.3; Carl Zeiss). The study was approved by and conducted according to requirements of the ethics committees at the Ludwig Maximilians University of Munich (20-1039) and the local ethics committee (EK 304/20, EK 119/20 and EK 092/20).

BM autopsies from patients with COVID-19 (embedded in paraffin) were deparaffinized with xilol twice for 5 min, ethanol (100%) twice for 2 min, ethanol (96%) once for 3 min, ethanol (70%) once for 2 min, and submitted for antigen retrieval with Tris-EDTA pH 9 for 20 min, washed once in 0.5% BSA-PBS-Tween-20 (0.1%) for 5 min. The samples were blocked in 10% donkey serum with 0.5% saponin for 1 h at room temperature. To monitor pDC activation and IFNα production, the following primary antibodies were used: mouse CD69 anti-human (MA5-15612, Thermo Fisher Scientific, 1:200), IFNα rabbit polyclonal (PA5-115430 Thermo Fisher Scientific, 1:50). Anti-CD123 goat polyclonal (ab257307, Abcam, 1:100) was used to label pDCs. Primary antibodies were incubated at 4 °C overnight and samples were subsequently washed three times with 0.5% BSA-PBS-Tween-20 (0.1%) for 5 min before adding secondary antibodies. Secondary antibodies (1:200) were as follows: donkey anti-rabbit-AF488 (A-21206), donkey anti-mouse-AF555 (A-31570) and donkey anti-goat-AF647 (A-21447), all from Thermo Fisher Scientific. DAPI (1:1,000) was used for nucleus staining (15 min at room temperature). The samples were washed three times with PBS 5 min before mounting with DAKO (S3023, DAKO) mounting medium.

### Immunohistology of mouse BM whole mounts

Mice were euthanized and bones (sternum, femur and tibiae) were collected and post-fixed in 4% PFA for 1 h at room temperature, and incubated in 15% sucrose for 2 h at 4 °C and in 30% sucrose at 4 °C overnight. Next, the bones were embedded in Tissue-Tek O.C.T. Compound and frozen and stored at −80 °C. Frozen bones were cut on the Histo Serve NX70 cryostat until the exposure of the BM. The sternum was cut as sagittal section. The femurs and tibiae were cut as coronal section or cross-section, according to purpose. Bones were carefully removed from O.C.T. and gently washed in 1× PBS. For whole-mount staining, the cut bones were fixed again in 4% PFA for 15 min at room temperature, washed in PBS and incubated in 10% normal goat serum (Thermo Fisher Scientific) for at least 45 min at room temperature (blocking/permeabilization). The bones were then incubated with primary antibodies at room temperature overnight and washed with PBS before adding secondary antibodies for 2 h at room temperature. Labelling of MKs/MKPs was as follows: primary antibodies: CD41–FITC^+^ (BioLegend, 133903, 1:100) and CD42-purified hamster anti-mouse (BioLegend, 148501, (1:100)); secondary antibodies: goat anti-hamster Alexa Fluor 647 (Abcam, ab173004, 1:100). Labelling of vessels was as follows: primary antibodies: anti-VE-cadherin (CD144) biotin purified (eBioacience, 13-1441-82, 1:100); secondary antibodies: streptavidin-PE (eBioscience, 12-4317-87, 1:200). Labelling of pDCs was as follows: primary antibodies: anti-SIGLECH-PE or FITC (BioLegend, 129606 or 129603, 1:100) or BST2 (CD317/PDCA-1, Thermo Fisher Scientific, PA5-120152, or eBioscience, 16-3172-81, 1:100) anti-mouse purified, anti-mouse rabbit polyclonal; secondary antibodies: goat anti-rat Alexa Fluor 647 (Abcam, ab150159) or goat anti-rabbit Alexa Fluor 594 (Thermo Fisher Scientific, A-11012) all at a dilution of 1:200. To label the nucleus, Hoechst 33342 or DAPI (Thermo Fisher Scientific, 1:1,000) was used. Lineage-biotin antibodies (Ter-119, CD3e, CD45R, CD11b, Ly-6G) and streptavidin-PE were used at a dilution of 1:200; all antibodies were purchased from eBioscience (San Diego). After staining, bone samples were imaged using the multiphoton LaVision Biotech TrimScope II system connected to an upright Olympus microscope, equipped with a Ti;Sa Chameleon Ultra II laser (Coherent) tunable in the range of 680 to 1,080 nm and a ×16 water-immersion objective (numerical aperture 0.8, Nikon). Single images were acquired at a depth of 50–80 μm, with a *z* interval of 2 μm. The signal was detected by photomultipliers (G6780-20, Hamamatsu Photonics, Hamamatsu). ImSpector Pro 275 (LaVision) was used as acquisition software. Alternatively, a LSM 880 laser-scanning confocal microscope equipped with an Aryscan module (Carl Zeiss), and the Zen Black acquisition software v.2.3 was used. The images were acquired using the Plan-Apo ×20/0.8 or ×63/1.46 objectives, z-step size of 2 µm, range in z-stack of 40 µm.

IFNα staining was as follows: primary antibodies: IFNα polyclonal antibody (PA5115430, Thermo Fisher Scientific, 1:100); secondary antibodies: goat-anti-rabbit 594 (Thermo Fisher Scientific, 1:200). Macrophage staining was performed as follows: primary antibodies: anti-CD68 monoclonal (Bio-Rad, MCA1957GA, 1:50); secondary goat-anti-rat Alexa 647 (Abcam, 1:200). IFNAR staining was performed as follows: primary antibody: IFNAR1 anti-mouse (BioLegend, 127302, 1:100); secondary antibodies: goat-anti mouse Alexa 555 (Thermo Fisher Scientific, 1:200). Bones were imaged using the LSM 880 confocal microscopy using the Airyscan module, objective Plan-Apo ×20 objective NA, 0.8 or with ×63/1.46 oil Plan-Apo. Images were taken with a *z* step size of 2 µm, range in *z* stack of 40 µm and analysed using Zen Blue v.2.3. 3D projections and rendering were performed using Imaris v.9.2 (Oxford Instruments/Imaris).

### Multi-photon intravital imaging of the calvarian BM

Anaesthetized mice were placed onto a metal stage with a warming pad to maintain the body temperature. The hair over the skull was carefully removed using an electric hair clipper. The skin on the skull was then cut in the midline to expose the frontal bone. For short-term imaging (<4 h), a custom-built metal ring was glued directly onto the centre of the skull, and the mouse’s head was immobilized by fixing the ring on a stereotactic metal stage. After imaging, the mice were euthanized by cervical dislocation. For long-term (chronic) imaging, a chronic window was implanted on the skull. In brief, a round cover glass (diameter: 6 mm) was centred on top of the frontal bone with sterile saline in between glass and the bone surface. The surrounding area of the glass was then filled with dental glue (Cyano veneer) and a custom plastic ring with inner diameter 8 mm was carefully centred on the frontal bone, with the glass exactly in the middle of the ring. The ring was further immobilized by applying the glue in the gap between the outer edge of the glass and the inner edge of the ring, as well as the gap between the outer edge of the ring and the tissue. Surgery was performed under sterile conditions. The mouse calvarium was imaged using a multiphoton LaVision Biotech TrimScope II system connected to an upright Olympus microscope, equipped with a Ti;Sa Chameleon Ultra II laser (Coherent) tunable in the range of 680 to 1,080 nm and additionally an optical parametric oscillator (OPO) compact to support the range of 1,000 to 1,600 nm and a ×16 water-immersion objective (NA 0.8, Nikon). Time-lapse videos of 3D stacks were recorded within 30 μm to 40 μm depth, with a *z* interval of 2 or 3 μm and a frame rate of 1 min. Chronic imaging was performed at frame rates of <6 h. Blood vessels and bone structure were taken as landmarks to retrieve the same imaging area of the BM. 3D *z* stacks were acquired with a *z* interval of 2 μm; 870 nm or 900 nm was used as an excitation wavelength. The signal was detected by Photomultipliers (G6780-20, Hamamatsu Photonics, Hamamatsu). ImSpector Pro 275 (LaVision) was used as acquisition software. Imaging was performed at 37 °C using a customized incubator. Blood vessels were visualized by intravenous injection of dextran tetramethylrhodamine 500,000 Da (TRITC-dextran, 100 μg in 100 μl solution, D7136, Thermo Fisher Scientific) or Dextran Cascade Blue 10,000 Da molecular mass (D1976, Thermo Fisher Scientific) before imaging. *Vwf-eGFP* mice were used to visualize the megakaryocytic lineage; pDCs were labelled with SIGLECH-PE antibody (BioLegend, 129606) injected intravenously 20 min before imaging (20 µl diluted with 100 µl NaCl).

### Image processing

Videos and images were analysed using Imaris v.9.2 (Oxford Instruments/Imaris) or ZEN Blue software v.2.3 (Carl Zeiss) or FIJI^68^. Image denoising using Noise2Void^69^ was performed in representative micrographs shown in Fig. 1c and Extended Data Fig. 2c. Mosaic images were stitched in Imaris. The numbers of MKs, MKPs and pDCs were quantified in the whole mosaic images and normalized to the total volume of the BM in the image. The cell distance to vessels and/or endosteal surface was measured manually in Imaris Slice mode or by using ZEN Blue (v.2.3). The mean diameter of an MKP or MK was calculated by the average of the longest and shortest axis of the cell. Cell volumes of 3D-rendered BM stacks were measured automatically in Imaris. Cell migration was analysed in 3D time-lapse videos by tracking the cell at every timepoint using Imaris. The cell speed was calculated by dividing the track length with the track duration. The distance of migrating pDCs to MK surfaces was measured and compared to computed random spots using Imaris v.10.9 (Oxford Instruments/Imaris).

### Isolation of mouse BM cells

Mice were anaesthetized and euthanized by cervical dislocation. Long bones (femurs, tibiae, humerus) were collected into ice-cold sterile PBS. Bones were flushed with PBS + 2% FCS using a 26-Gauge needle and the BM suspension was further filtered through a 70 μm or 100 μm cell strainer (Miltenyi Biotec) and pelleted at 4 °C and 300*g* for 5 min. The supernatant was discarded and cells were resuspended and incubated in red blood cell lysis buffer for 5 min. Lysis was terminated by adding 30 ml PBS + 2 mM Ethylenediaminetetraacetic acid (EDTA, Sigma-Aldrich), followed by centrifugation at 4 °C and 300*g* for 5 min. Cells were resuspended with PBS + 0.5% BSA (Carl Roth).

### Flow cytometry

BM isolated cells (as described above) were enriched by removing CD19^+^ and CD11b^+^ cells by negative selection using the EasySep selection kit II (StemCell Technologies) for the cell sorting experiments. Cells were incubated with mouse CD16/CD32 (BD Pharmingen (Fc block) before staining (1:100). The following antibodies were used to identify MKs: 1:100 anti-mouse CD41-FITC^+^ and anti-mouse CD42d-APC^+^ (BioLegend, 1:100); and MKPs: anti-mouse CD41-FITC^+^, Pacific Blue Lin^−^ (Ter-119^−^CD3e^−^CD45R^−^CD11b^−^Ly-6G^−^), anti-mouse CD105-PE/PercCy7^−^, CD150-Brillant violet 510^+^ and anti-CD9-PercCy5.5^+^ (BioLegend) (all 1:100). We identified pDCs using the following antibodies: anti-mouse SIGLECH-FITC^+^, CD11b-PE-Cy7^−^ and B220-APC^+^ from BioLegend (1:100). pDC activation: anti-mouse CD69-FITC (1:200), CD86-PE (1:400), CD11b-APC-Cy7 (1:200), CD317-APC (1:100), SiglecH-PercCy5.5 (1:100) antibodies all from BioLegend and Life/Dead fixable Aqua dead marker (405 nm excitation; Thermo Fisher Scientific, 10 μg ml^−1^); macrophages: anti-CD45.2^+^ (BioLegend, PE/Cyanine 7, 1:200), anti-CD45.1 (BioLegend, FITC 1:100), anti-F4/80^+^ (BioLegend, PerCP/Cyanine 5.5, 1:100), anti-CD64^+^ (BioLegend, APC, 1:100); anti-CD115^−^ (BioLegend, Brilliant Violet 421, 1:100); neutrophils: CD11b^+^ (BioLegend, APC/Cyanine 7, 1:200), Ly6G/G1^+^ (BioLegend, PE/Cyanine 7, 1:200), CD115^−^ (BioLegend, Brilliant Violet 421, 1:100); p-IRF7 expression by pDCs: after staining for pDC surface markers (see above), cells were fixed with PFA and methanol and stained with anti-mouse rabbit monoclonal phospho-IRF7 antibody (Ser437/438, Cell Signaling, 1:100) in Perm buffer III (BD) as previously described^42^ followed by secondary goat anti-rabbit-APC antibodies (Thermo Fisher Scientific, 1:200). Before loading the samples, 10 μg ml^−1^ Sytox Orange for the live/dead cell gating and counting beads (1,2,3count beads, Thermo Fisher Scientific), were added to the cell suspension, with exception of the p-IRF7 stain. Apoptosis was measured using Apotracker Green (BioLegend) according to the manufacturer’s instructions. For reticulated platelet staining, 2 µl of blood was fixed with PFA 1%. The blood samples were stained with anti-CD42d-APC (1:100) and thiazole orange (TO) (1 μg ml^−1^) (Sigma-Aldrich) for 25 min at room temperature in the dark and submitted to flow cytometry analyses^70^. MK ploidy was quantified after propidium iodide staining in MKs. Measurements were performed on the FACS Canto II cell analyzer equipped with FACSDiva software v.6.0 (BD Biosciences) or on the Cytoflex-S system with CytExpert acquisition software v.2.3 (Beckman Coulter). FACS data were analysed using FlowJo v.10.6.2 or v.10.9. The gating strategies for all FACS data are shown in Supplementary Data 1.

### Bulk RNA-seq analysis

For RNA-seq analysis, BM cells were isolated by flushing the long bones with FACS buffer (2 mM EDTA, 1% FCS, PBS) and treated with Pharm Lyse buffer (BD). Cells were enriched by magnetic removal of CD11b^+^ and CD19^+^ cells (EasySep, Stem Cell Technologies). The negative fraction was stained for B220-BV421, SIGLECH-PE, CD9-PerCP-Cy5.5, CD41-FITC, CD42-APC, KIT-APC-Cy7 (all from BioLegend, (1:100). A total of 2,000 cells was sorted using the BD FACS ARIA III Cell sorter (FACSDiva acquisition software v.7.0), into NEB-lysis buffer and processed for sequencing using the NEBNext Single Cell/Low Input RNA Library Kit according to the manufacturer’s protocol (at IMGM). Libraries were pooled in equimolar amounts and sequenced on the NovaSeq 6000 (Illumina) system in a single-end 75-nucleotide run, yielding between 15 and 25 million reads per sample. Reads were mapped against GRCm38.p4 using CLC Genomics Workbench (Qiagen) with the following parameters: mismatch cost 2; insertion/deletion cost, 3; length fraction, 0.8; similarity fraction 0.8; global alignment “no”; strand specific “both”; maximum number of hits per read 5. CLC Genomics Workbench was also used to generate gene expression matrices.

#### GSEA

To prepare the data for gene set enrichment analysis (GSEA), DESeq2 (v.1.30.0) analysis was performed using Galaxy with the default parameters^71,72^. Genes were filtered for an expression of transcripts per million (TPM) > 1 in any condition (42,868 genes) to remove non-expressed or very-low-abundance genes, and then sorted according to the log_2_-transformed fold change of the respective analysis. For further analysis, the tool GSEA (v.4.0.3) of UC San Diego and Broad Institute was used^73,74^, referring to their RNA-seq manual pages for analysis. The normalized counts of each replicate as the ranked list generated above were submitted to the GSEA tool with the following parameters: gene sets of their Molecular Signatures Database (MSigDB) in the categories ‘canonical pathways’ (C2) and ‘gene ontology’ (C5) were chosen to contain *Ifna1* gene (94 gene sets). Mouse gene symbols were mapped to the human gene symbol (Chip platform: Mouse_Gene_Symbol_Remapping_Human_Orthologs_MSigDB.v7.4.chip), permutation type was set to gene set and gene set size was set to contain between 15 and 2,000 genes.

#### GO analysis

Genes were filtered for log_2_-transformed fold change greater or lower than 1 and submitted to the Database for Annotation, Visualization and Integrated Discovery (DAVID) v.6.8 (ref. ^75^). Resulting GO terms were filtered for *q* < 0.05. The data visualization tool ClustVis (http://biit.cs.ut.ee/clustvis) was used to generate the heat map of genes expressed in MKPs in Extended Data Fig. 7 (ref. ^76^). Bulk RNA-seq data are accessible at the Gene Expression Omnibus (GEO; GSE185488).

### Sample preparation for scRNA-seq

BM cells were isolated as described above, by flushing the long bones with PBS + 2% FCS, without EDTA using a 26 gauge needle. The BM suspension was further filtered through a 70 μm and 40 μm cell strainer (Miltenyi Biotec) and pelleted at 4 °C and 300*g* for 5 min. The pellet was resuspended with 1 ml 1× red blood cell lysis buffer and incubated at room temperature for 5 min. After incubation, 15 ml of PBS + 2% FCS without EDTA was added. The cell suspension was centrifuged at 300*g* for 5 min, the supernatant was discarded and the pellet was resuspended in 1 ml PBS + 2% FCS, and a negative selection kit for CD11b^+^ and CD19^+^ (StemCell Technologies) was used according ot the manufacture’s instructions to remove the CD11b^+^ and CD19^+^ cells. The final pellet was incubated with the respective TotalSeqB anti-mouse Hashtag antibody (that is, BioLegend, TotalSeq-B0301 anti-mouse Hashtag 3; of this family, Hashtags 3, 4, 5 and 10 were used). After incubation for 30 min on ice and three subsequent washing steps, cells were resuspended in FACS buffer with 2% FBS, followed by centrifugation at 300*g* for 5 min at 4 °C. The supernatant was discarded and the cell pellet was stained for MKPs as described above. MKPs were sorted using BD FACSMelody Cell Sorter (BD FACS Chorus acquisition software v.1.1.20.0), for 10x scRNA-seq analysis.

### scRNA-seq

The Chromium Next GEM Single Cell 3′ reagent kit v3.1 (CG000206 Rev D) from 10x Genomics protocol was used for sequencing of FACS-sorted BM MKPs. To decrease batch-effect related artefacts, sample multiplexing using TotalSeqB anti-mouse Hashtag antibodies, which were included into the FACS antibody mix, was performed. Four samples were multiplexed into one library. In total, 1 × 10^5^ cells across runs were loaded for generating gel beads in emulsion (GEMs). According to the kit protocol, first, GEMs were generated, then reverse transcription was performed, and cDNA was cleaned up, amplified and size selected. After a quality control and quantification step, gene expression libraries and cell surface libraries were subsequently constructed. The libraries were sequenced using the Illumina NovaSeq system by IMGM laboratories, as described previously^77^.

### Analysis of scRNA-seq data

Sequencing reads were processed using the Cell Ranger software with the mm10 mouse reference genome index provided by 10x Genomics (https://cf.10xgenomics.com/supp/cell-exp/refdata-gex-mm10-2020-A.tar.gz). This resulted in a count matrix for 16,045 cells and 32,285 genes. The count data were analysed using Seurat^78^. Background contamination was removed using the soupX method, setting the contamination fraction parameter of 0.1 (ref. ^79^). Quality control included removal of cells with less than 250 or more than 6,000 features (expressed genes), removal of cells with total UMI counts below 400 and above 20,000, removal of cells with more than 5% of UMIs mapping to mitochondrial genes and removal of genes expressed in less than 3 cells. Furthermore, ribosomal genes were removed. Count data were size normalized to a total UMI count of 10,000 per cell and subsequently log transformed (plus one pseudocount). The top 2,000 highly variable genes were selected on the basis of VST (variance stabilizing transformation)-transformed expression values. Cell cycle scoring was performed and expression values were adjusted for the percentage of mitochondrial UMIs, the S and G2M cell cycle scores. Cells were assigned to samples by demultiplexing the Hashtag oligos, resulting in 1,918 cells for control, 3,243 cells for platelet depletion plus pDC depletion and 1,900 cells for platelet depletion. For differential gene expression analysis, expression levels per gene were centred and scaled across cells. Nearest neighbour graphs (*k* = 30) were built based on the first 30 principal components. On the basis of the graph, ten clusters were identified using the Leiden algorithm with a resolution of 0.25. Cluster-specific marker genes were identified using Wilcoxon tests, testing only for overexpression, requiring at least 25% of the cluster to express the marker and a log-transformed fold change of at least 0.25. Clusters were assigned to cell types based on the gene annotations of these marker genes. For each cluster, differential gene expression analysis between conditions was performed using the Wilcoxon rank-sum test (wilcox). The DE genes were then selected based on an average log_2_-transformed fold change cut-off of greater than 0.25 and an adjusted *P*-value cut-off of less than 0.05. Cell-type-specific gene expression of the gene sets defined from bulk RNA-seq analysis were summarized into gene scores (average expression across the gene set) and visualized by cell type cluster. Trajectory analysis was performed on the following cell types: metabolic MKPs, late MKP, MK-MEPs, cycling MK-MEPs and early MKPs using Monocle3^80,81^. This assigned each cell to an estimated pseudotime along a trajectory. The graph_test function was used to determine genes with pseudotime-associated gene expression patterns (FDR < 0.05 and Moran’s *I* > 0.25). Gene expression values of genes with pseudotime-associated gene expression were fitted using a spline function with 3 degrees of freedom and corresponding *z* scores were visualized as a heat map. scRNA-seq data are accessible at the GEO (GSE261996). Code is available at GitHub (https://github.com/heiniglab/gaertner_megakaryocytes).

### MK culture from mouse BM

BM cells (see above) were cultured in DMEM medium containing 10% fetal bovine serum, 1% penicillin–streptomycin and 70 ng μl^−1^ TPO (ImmunoTools) for 5 days at 37 °C and 5% CO_2_. On day five, a BSA step gradient was prepared by placing PBS containing 1.5% BSA on top of PBS with 3% BSA (PAA). Cells were loaded on top of the gradient, and MKs were settled to the bottom within 30 min at 1× gravity at room temperature. Mature MKs formed a pellet at the bottom of the tube.

### In vitro co-culture of pDCs with MKs

For pDC generation, BM cells were isolated (see above) from control and *Myd88*^*−/−*^ mice and cultured for 7 days in RPMI-1640 GlutaMAX-I (GIBCO) supplemented with 10% FCS (GIBCO), 1 mM sodium pyruvate (GIBCO), 1% penicillin–streptomycin (Thermo Fisher Scientific), 1% MEM non-essential amino acids (GIBCO), 0.05 mM β-mercaptoethanol MeEtOH (GIBCO) and recombinant 100 ng ml^−1^ FLT3L (BioLegend). Cells were collected by flushing Petri dishes with cold PBS. The purity of pDCs was 70–75% as determined by FACS. MK-iDTR mice were injected with DT to induce death of MKs. Control mice received PBS. After 6 h of DT injection, mice were euthanized and femurs were flushed with DMEM medium containing 10% fetal bovine serum, 1% penicillin–streptomycin and 70 ng ml^−1^ thrombopoietin (TPO, ImmunoTools). MKs were isolated using a BSA gradient as described above. pDCs and MK (1:1) were incubated together for 8 h at 37 °C and 5% CO_2_. After incubation, the supernatant was collected and analysed for IFNα level (ELISA, see below).

### ELISA

For serum TPO measurement, 1 ml anti-coagulated blood was collected intracardially and kept overnight at −20 °C. The next day, the blood was centrifuged at 2,000*g* for 20 min and the supernatant (serum) was collected for TPO measurement using the Quantikine Mouse Thrombopoietin ELISA Kit (R&D Systems) to measure the serum TPO levels. IFNα was measured by ELISA (Mouse IFN Alpha All Subtype ELISA Kit, High Sensitivity, PBL Assay Science). Blood was left at room temperature for 20 min and, after centrifugation, the serum was frozen at −20 °C until further analysis. To measure the IFN levels in the BM, one femur was flushed with 200 µl of PBS and cells were centrifuged at 300*g*. The supernatants were stored at −20 °C until analysis.

### pDC culture with MK supernatants and DNase treatment

*PF4-cre;iDTR*^*fl/fl*^ mice were treated with DT for 6 h. The long bones were collected and the BM was isolated by flushing the femurs, tibias and humerus with 200 µl of PBS + 2% FCS using a 26 gauge needle. The BM suspension was further filtered through a 100 μm cell strainer (Miltenyi Biotec) and pelleted at 4 °C and 300*g* for 5 min. The supernatant was discarded and cells were resuspended and incubated in red blood cell lysis buffer for 5 min. The MKs were isolated as described above and cell suspension was centrifuged for 5 min at 5,000*g* and 4 °C, followed by 1 min at 11,000*g* to obtain a tight pellet. The supernatant was collected and transferred to new tubes and centrifuged for 15 min at 2,500*g* at room temperature (Eppendorf 5415D with the F45-24-11 rotor; Eppendorf). To obtain the MP pellet, the supernatant was transferred into new tubes (homo-polymer, Axygen) and centrifuged for 40 min at 20,000*g* at room temperature (Mikro200R with the 2424-B rotor; Hettich)^82^. The resultant MK pellet was collected and the supernatant containing exosomes/extracellular vesicles was transferred into a new tube and treated or not with DNase (1 µl ml^−1^; Sigma-Aldrich) at 37 °C for 20 min. The treated or non-treated supernatant was added to the pDC cell culture and incubated of 60 min at 37 °C. The pDC supernatant was collected and the IFNα levels were measured using the ELISA kit according to the manufacturer’s instructions (Mouse IFN Alpha All Subtype ELISA Kit, High Sensitivity, PBL Assay Science). The pDCs were collected and stained for FACS analysis for pDC-activation markers (anti-CD69 (1:200) and anti-CD86 (1:400)).

### NanoDrop experiment

A NanoDrop spectrophotometer (Thermo Fisher Scientific, NANODROP 2000, Peqlab), was used to measure the concentration of DNA in a 2 µl drop of the MK apoptotic supernatant treated or non-treated with DNase I.

### MK CFU assay

CFU assays were performed using the MegaCult kit (StemCell Technologies) according to the manufacturer’s protocol. In brief, femurs and tibias of *Vwf-cre;Ifnar*^*−/−*^, *Vwf-cre;Ifnar*^*−/−*^, *Ifnar*^*−/−*^ and *Ifnar*^*+/+*^ mice were flushed with Iscove’s MDM with 2% FBS to isolate BM cells. Cells were washed in Iscove’s MDM (without FBS) before culture. Then, 2.2 × 10^6^ cells were resuspended in cold MegaCult-C medium containing collagen, TPO 50 ng ml^−1^ and IFNα type 1 universal (5 U, 10 U, 100 U, 500 U or 1,000 U; PBL-Biomedical Laboratories). The final cell suspension (1.5 ml) was loaded into six-well plates and cultivated for 7 days at 37 °C under 5% CO_2_. After incubation, well plates were imaged using a stereo microscope (Axio Zoom v16 with Objective Plan-NEOFLUAR Z ×1.0/0.25 FWD 56 mm) and Zen Blue software (v.2.6) was used for imaging acquisition (Carl Zeiss). MK-CFUs colonies were classified according to the manufacturer’s protocol (a minimum of 3 cells in close contact).

### EdU proliferation assay

The Click-it EdU Cell Proliferation Assay Kit (Thermo Fisher Scientific) was used to analyse the MKP proliferation. In vivo labelling of BM cells with 5-ethynyl-2′-deoxyuridine (EdU) was described previously^83^. In brief*, Vwf-eGFP* mice were intraperitoneally injected with 0.5 mg EdU in DMSO. After 4 h, mice were anaesthetized and euthanized by cervical dislocation and long bones (femurs and tibiae) were collected. BM cells were prepared as described above. The detection of EdU was performed according to the manufacturer’s protocol. In brief, cells were stained with surface marker antibodies (CD41, CD42) for 30 min at room temperature in the dark, followed by fixation for 15 min (4% PFA, provided in the kit) and permeabilization for 15 min (saponin-based permeabilization and wash reagent, provided in the kit). The samples were washed with 1% BSA between each step. The samples were then incubated for 30 min at room temperature in the dark in EdU reaction cocktail containing PBS, copper protectant, Pacific Blue picolyl azide and reaction buffer additive according to the manufacturer’s protocol. The samples were next washed and analysed by flow cytometry (LSRFortessa cell analyzer equipped with BD FACSDiva v.8.0.1, from BD Biosciences). VWF^+^CD41^+^CD42^−^ cells were gated and EdU^+^ cells were measured within this population using FlowJo (v.10.6.2).

### RT–PCR analysis of *Ifnar1*

MKs and MKPs from unfractionated mouse BM cell suspensions were directly sorted into RLT buffer (Qiagen) containing 143 mM β-mercaptoethanol (Sigma Aldrich) and total RNA was isolated using the RNeasy Micro Kit (Qiagen) including an on-spin column DNase I digest to remove remaining traces of genomic DNA. First-strand cDNA was synthesized from total RNA with the High Capacity cDNA Reverse Transcription kit (Applied Biosystems) using random primers in 20 µl reaction volumes. RT–PCR was performed using the SsoAdvanced Universal SYBR Green Supermix (Bio-Rad) and the primers for murine *Ifnar1* and *Actb* in the MyiQ Single-Colour Real-Time PCR System (Bio-Rad). Products of RT–PCR were separated by electrophoresis on a 2.5% agarose gel in 1× TBE buffer. Images were taken using a Gel iX Imager (Intas). Primers were as follows: Mm_Ifnar1 Fw, TCTCTGTCATGGTCCTTTATGC (Eurofins); Mm_Ifnar1 Rev, CTCAGCCGTCAGAAGTACAAG (Eurofins); and the Mm_Actb_1_SG primer assay (400 × 25 µl reactions; QT00095242, Qiagen).

### Statistics

GraphPad Prism (v.9.1.2) was used for all statistical analysis. All data were assumed to have Gaussian distribution, unless otherwise specified. Before performed the statistical analysis, the data were confirmed to have equal variance using *F*-tests, and Student’s unpaired *t*-tests were used for the comparison of two groups; otherwise, unpaired *t*-tests with Welch’s correction were used when variances were significantly different. For comparison of multiple groups, one-way or two-way ANOVA was used. Error bars indicate the s.d. All reported probabilities were two-sided. *P* < 0.05 was considered to be significant.

### Reporting summary

Further information on research design is available in the Nature Portfolio Reporting Summary linked to this article.

## Online content

Any methods, additional references, Nature Portfolio reporting summaries, source data, extended data, supplementary information, acknowledgements, peer review information; details of author contributions and competing interests; and statements of data and code availability are available at 10.1038/s41586-024-07671-y.

### Supplementary information

Supplementary Data 1 Gating strategies for FACS analysis.

Reporting Summary

Supplementary Table 1 List of differentially expressed genes of MKP clusters (related to Fig. 4g) and a full list of differentially expressed genes shown in pseudotime heat map (related to Fig. 4h).

Supplementary Table 2 Patient characteristics **(**related to Fig. 4a–g).

Supplementary Table 3 List of all reagents and resources with the source and identifier.

Supplementary Video 1 MKs and MKPs reside along BM sinusoids. Two-photon microscopy of sternal whole-mount (2D slices and 3D rendered stack are shown and animated). MKs (orange; CD42^+^CD41^+^); MKPs (green; CD42^−^CD41^+^); sinusoids (grey; CD144^+^); bone (blue; SHG). Related to Fig. 1a.

Supplementary Video 2 Thrombopoiesis and megakaryopoiesis are synchronized processes. Chronic 2P-IVM of cavarial bone marrow. MKs/MKPs: VWF–eGFP (green); blood vessels: TRITC-dextran (magenta); bone: second harmonic generation (blue). Animation of 3D rendered data shows mature MKs (colour coded in cyan) that disappear from the niche and small MKPs (colour-coded in yellow) that appear and grow in size. 3D stacks were recorded at the four indicated timepoints. Related to Fig. 1c and Extended Data Fig. 2c.

Supplementary Video 3 Migrating pDCs monitor megakaryocytes in the bone marrow. 2P-IVM videos of pDCs (magenta; anti-SiglecH-PE) migrating in close proximity to MKs (green; VWF–eGFP^+^) in the bone marrow. Maximum intensity projections of raw data and 3D rendered animations are shown. Related to Fig. 2a and Extended Data Fig. 4g.

### Source data

Source Data Fig. 1

Source Data Fig. 2

Source Data Fig. 3

Source Data Fig. 4

Source Data Fig. 5

Source Data Extended Data Fig. 1

Source Data Extended Data Fig. 2

Source Data Extended Data Fig. 3

Source Data Extended Data Fig. 4

Source Data Extended Data Fig. 5

Source Data Extended Data Fig. 6

Source Data Extended Data Fig. 7

Source Data Extended Data Fig. 8

Source Data Extended Data Fig. 9

## Data Availability

Imaging and flow cytometry raw data are available on request. scRNA-seq data are accessible at the GEO (GSE261996). Bulk RNA-seq data are accessible at the GEO (GSE185488). Source data are provided with this paper.

## Code availability

Code for scRNA-seq analysis is available at GitHub (https://github.com/heiniglab/gaertner_megakaryocytes).
