# Supplementary material for: Plasmacytoid dendritic cells control homeostasis of megakaryopoiesis
Source: Nature. 2024 Jul 10;631(8021):645–53. doi: 10.1038/s41586-024-07671-y (PMC11254756; doi:10.1038/s41586-024-07671-y)

nature portfolio

https://doi.org/10.1038/s41586-024-07671-y

Supplementary information

Plasmacytoid dendritic cells control homeostasis of megakaryopoiesis

In the format provided by the authors and unedited

Gating strategy of apoptotic MKs related to Fig. 1l

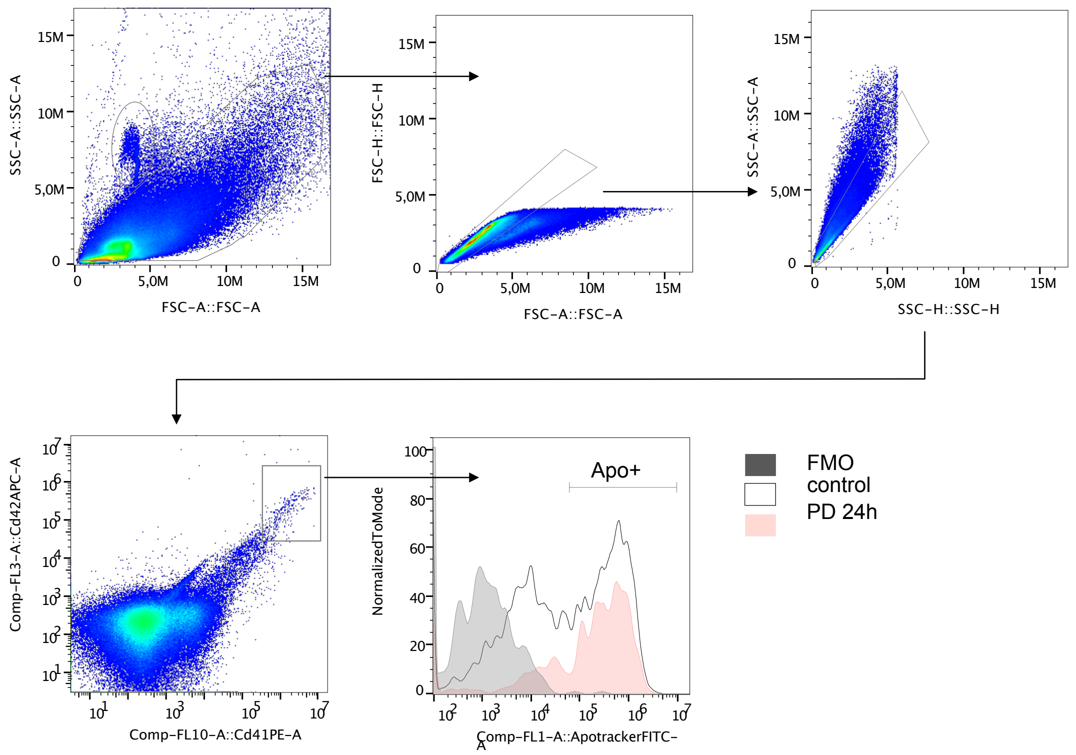

Gating strategy of reticulated platelets related to Fig. 2d, e, 3f

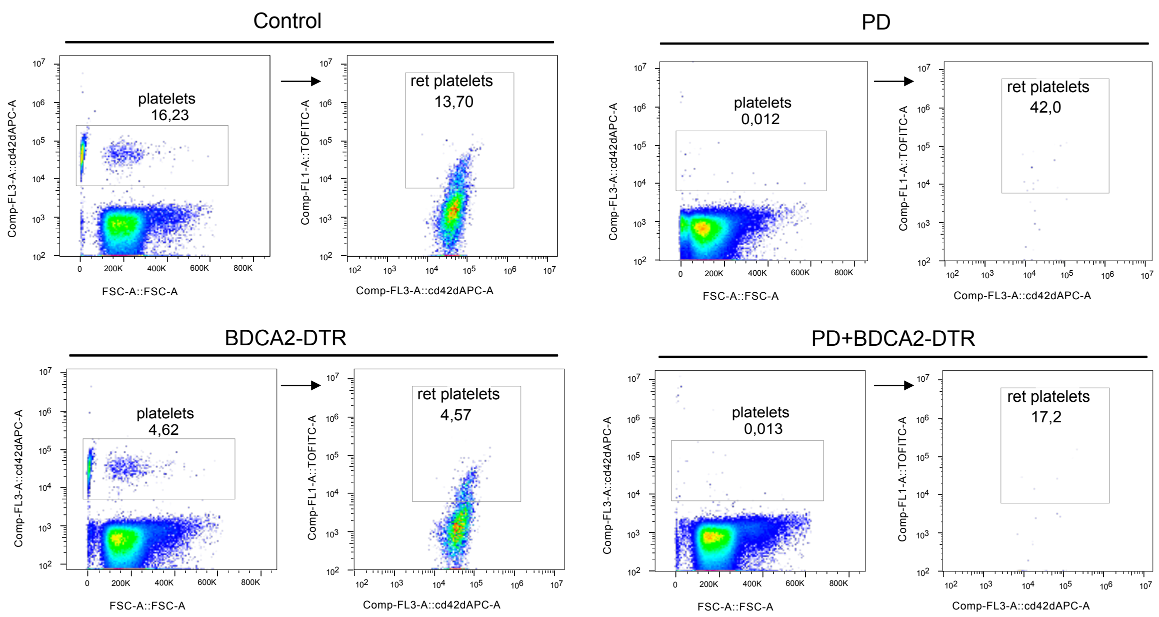

Gating strategy of MKPs, MKs and pDCs related to Figs. 2e, 3f, 4b,c,l and ED Figs. 4b-d, 5b,f, 8f

flow cytometry

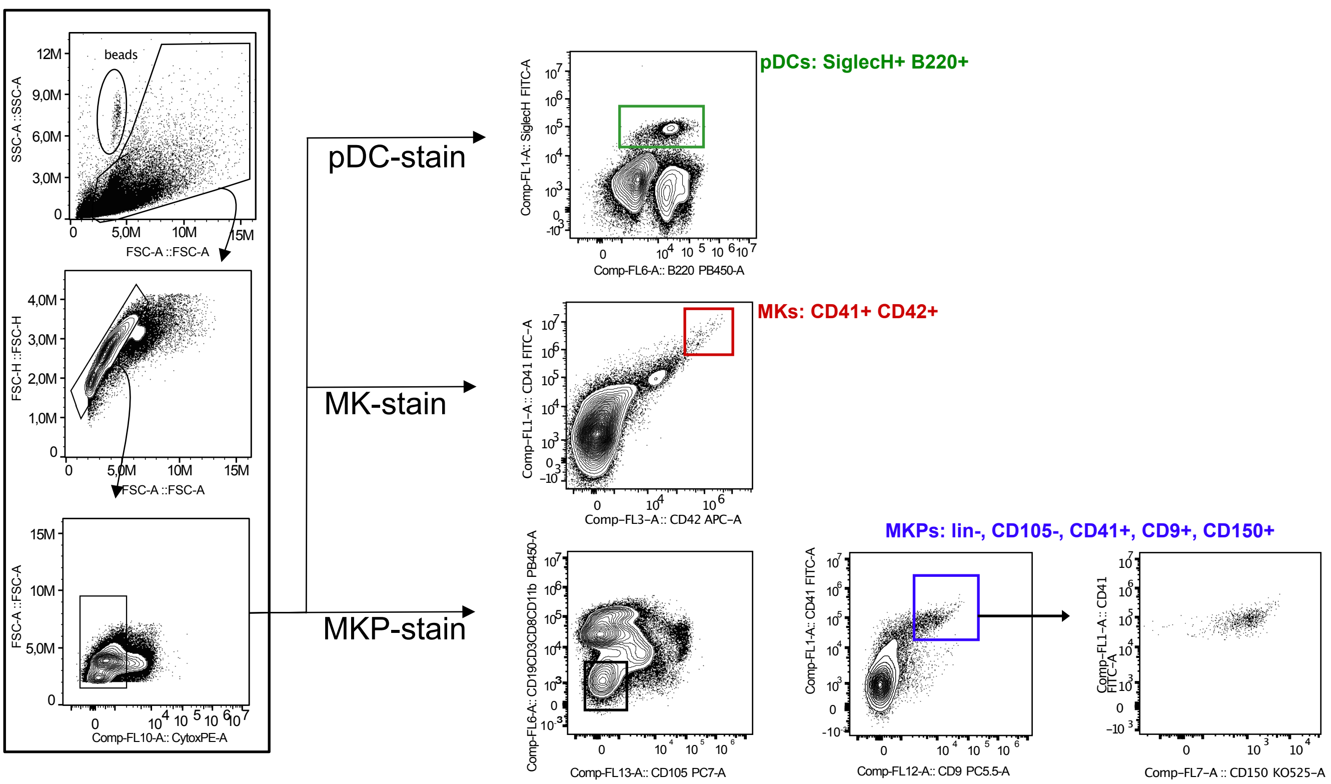

sorting gate

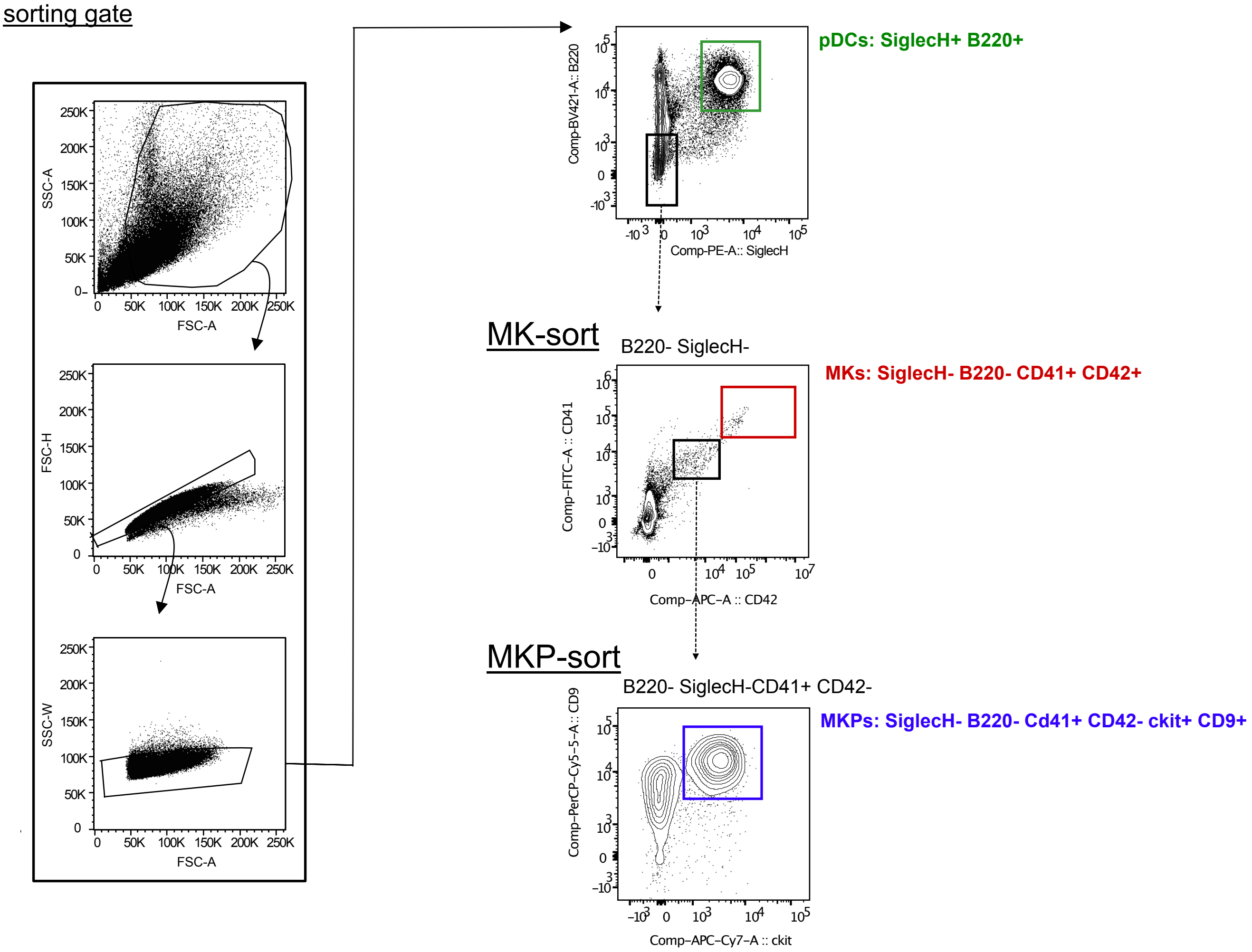

Gating strategy of p-IRF7 expressing pDCs related to Fig. 3c

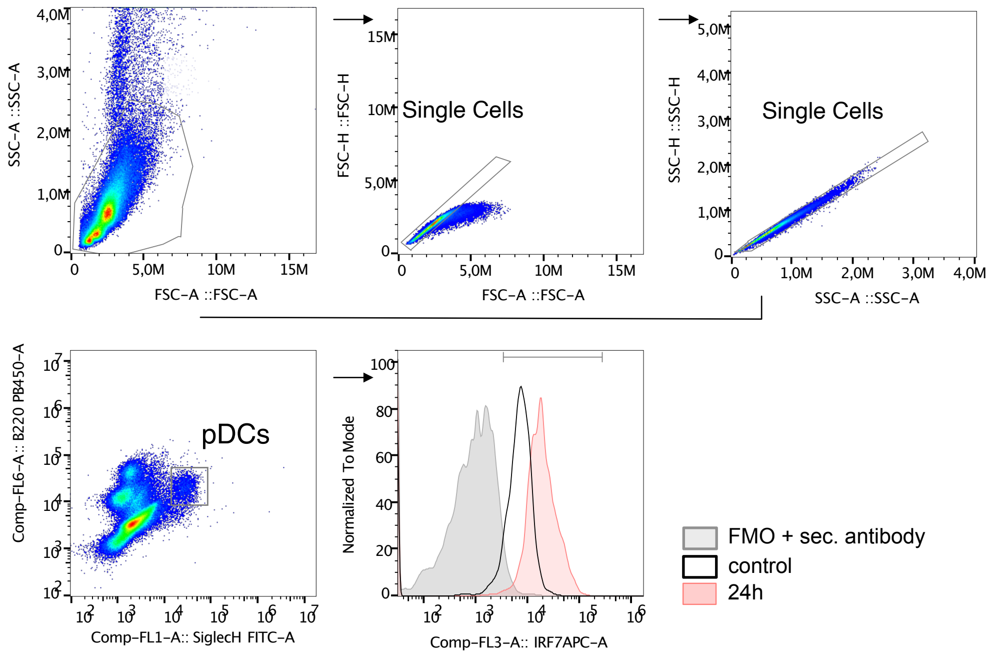

Gating strategy of EdU-positive MKPs related to ED Fig. 2g

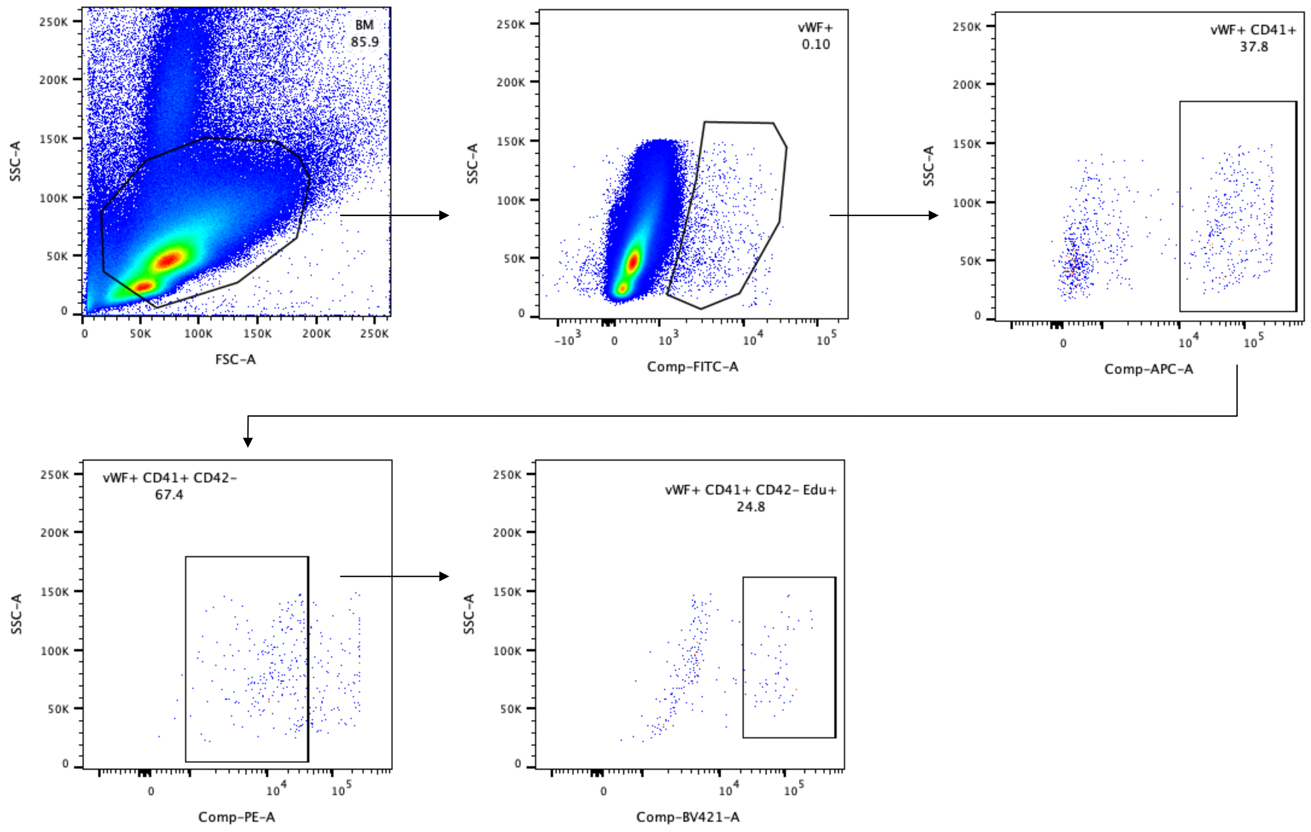

Gating strategy of macrophages related to ED Fig. 4b

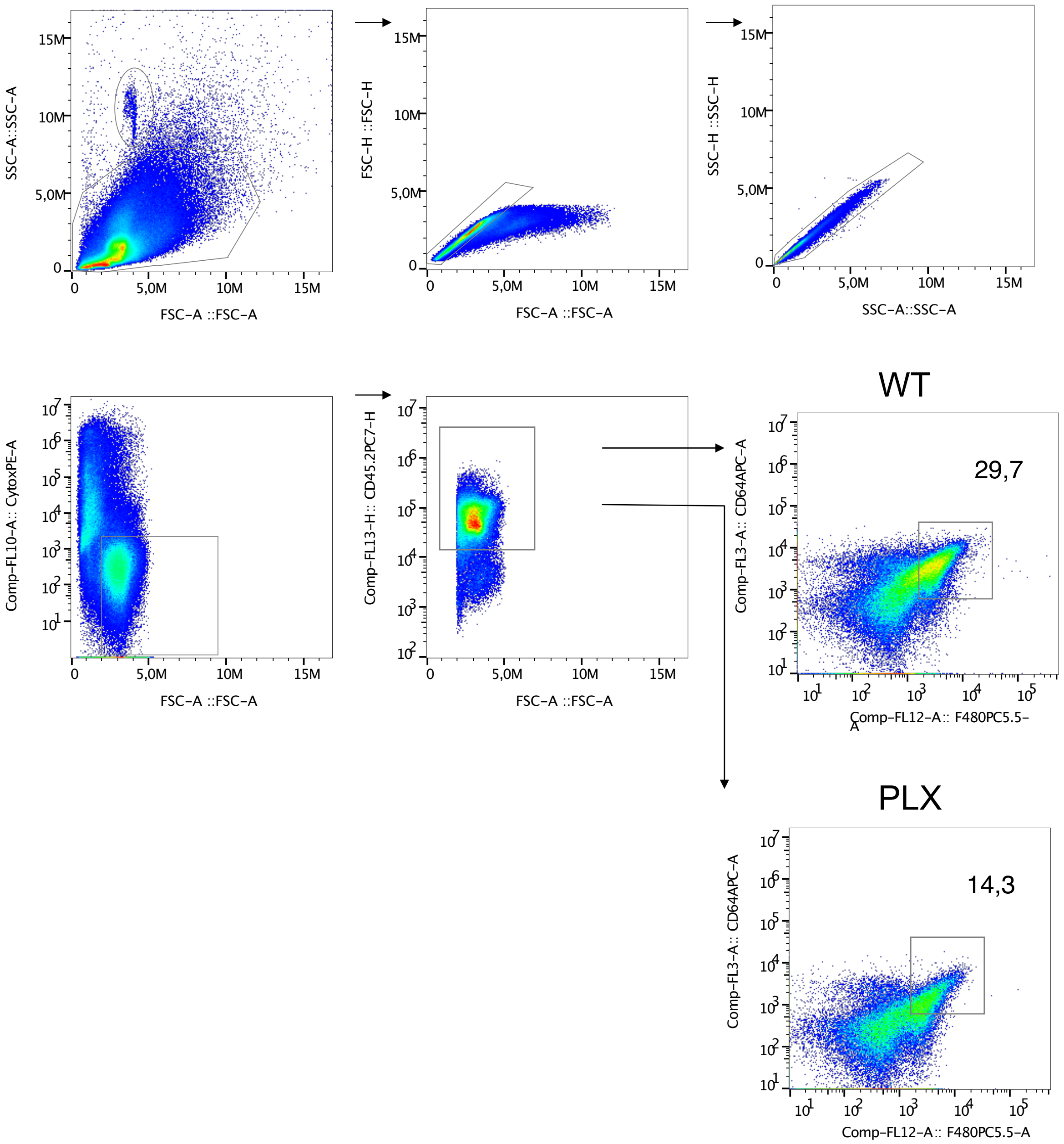

Gating strategy of macrophages related to ED Fig. 4c

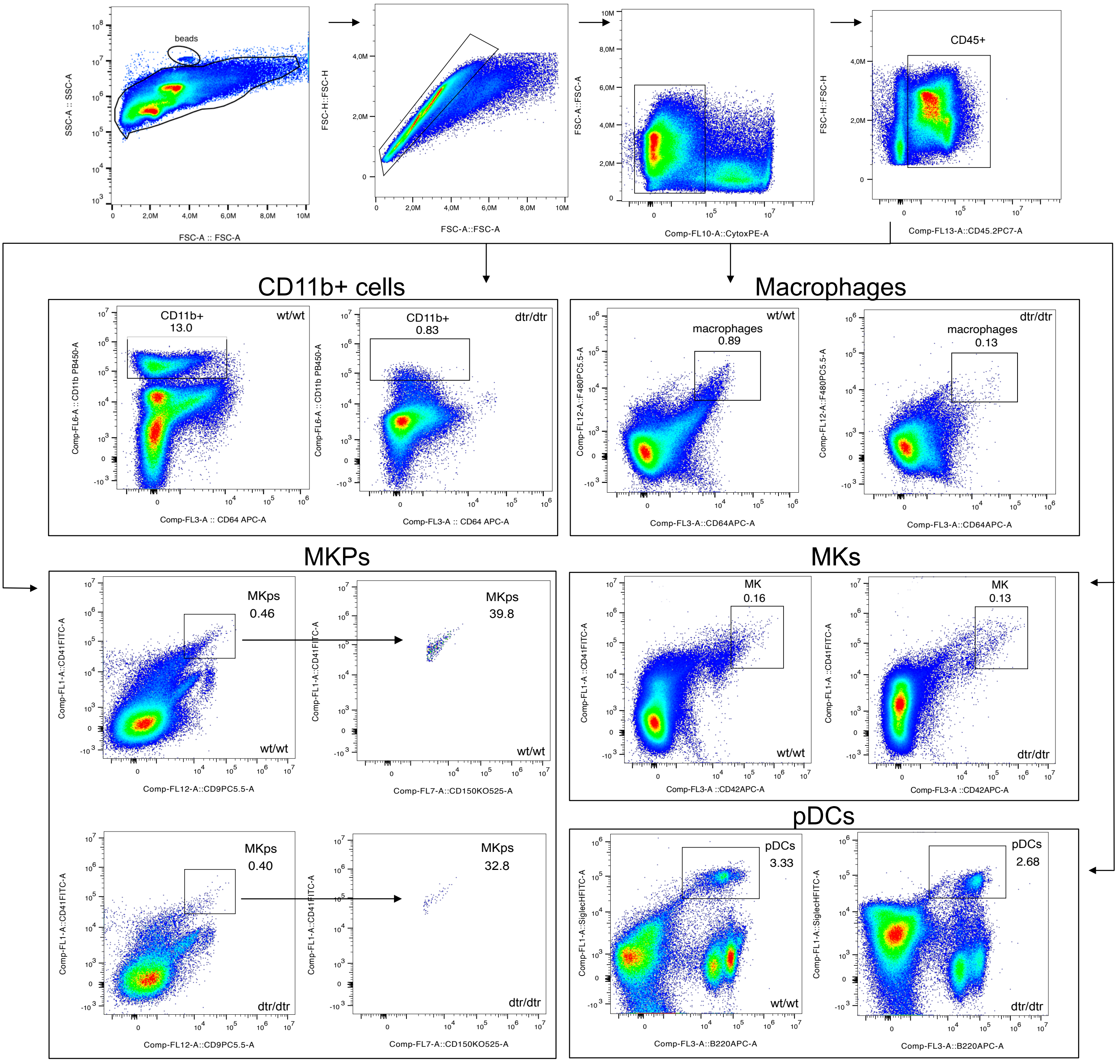

Gating strategy of neutrophils related to ED Fig. 4d

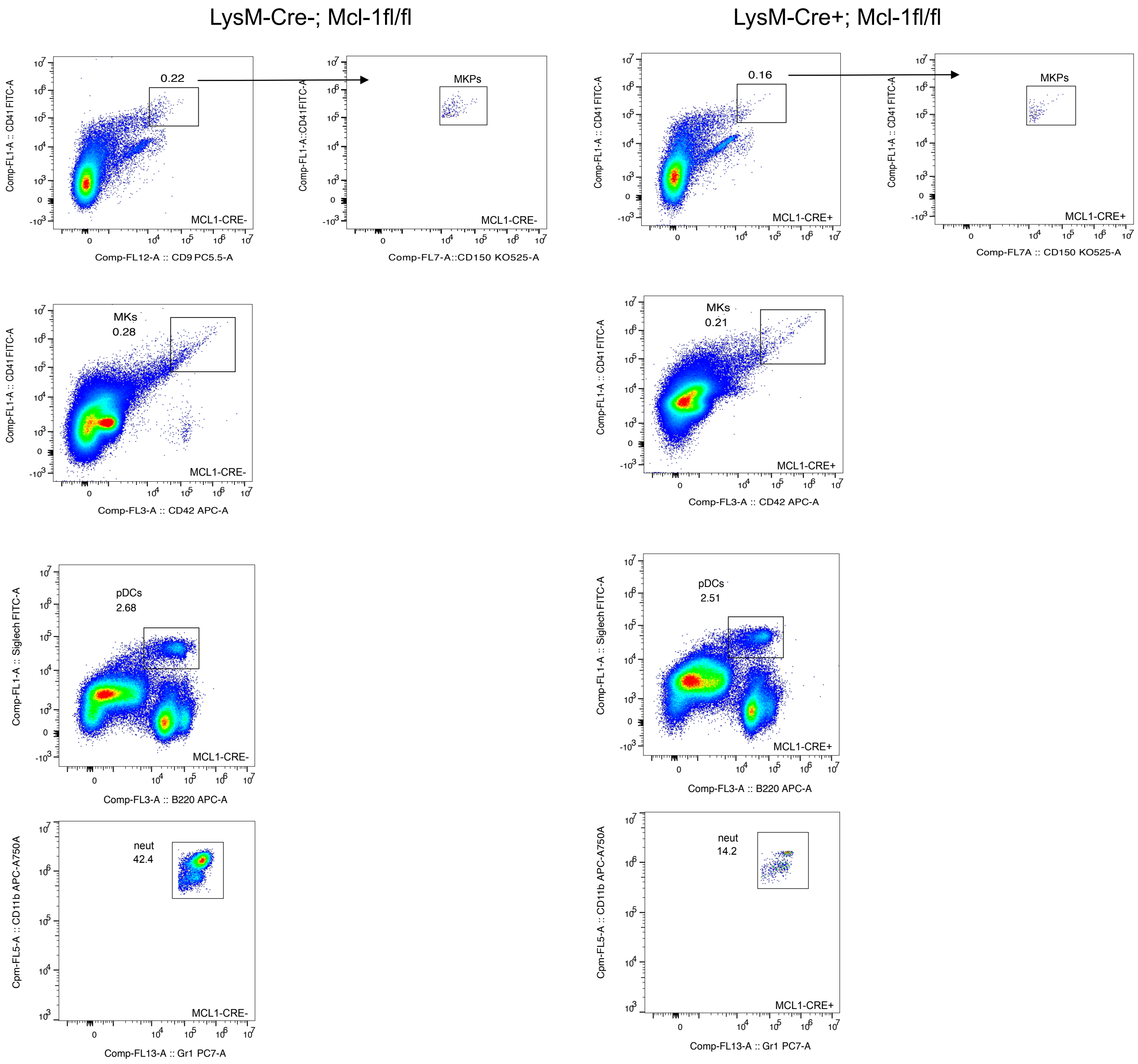

Gating strategy related to ED Fig. 5c

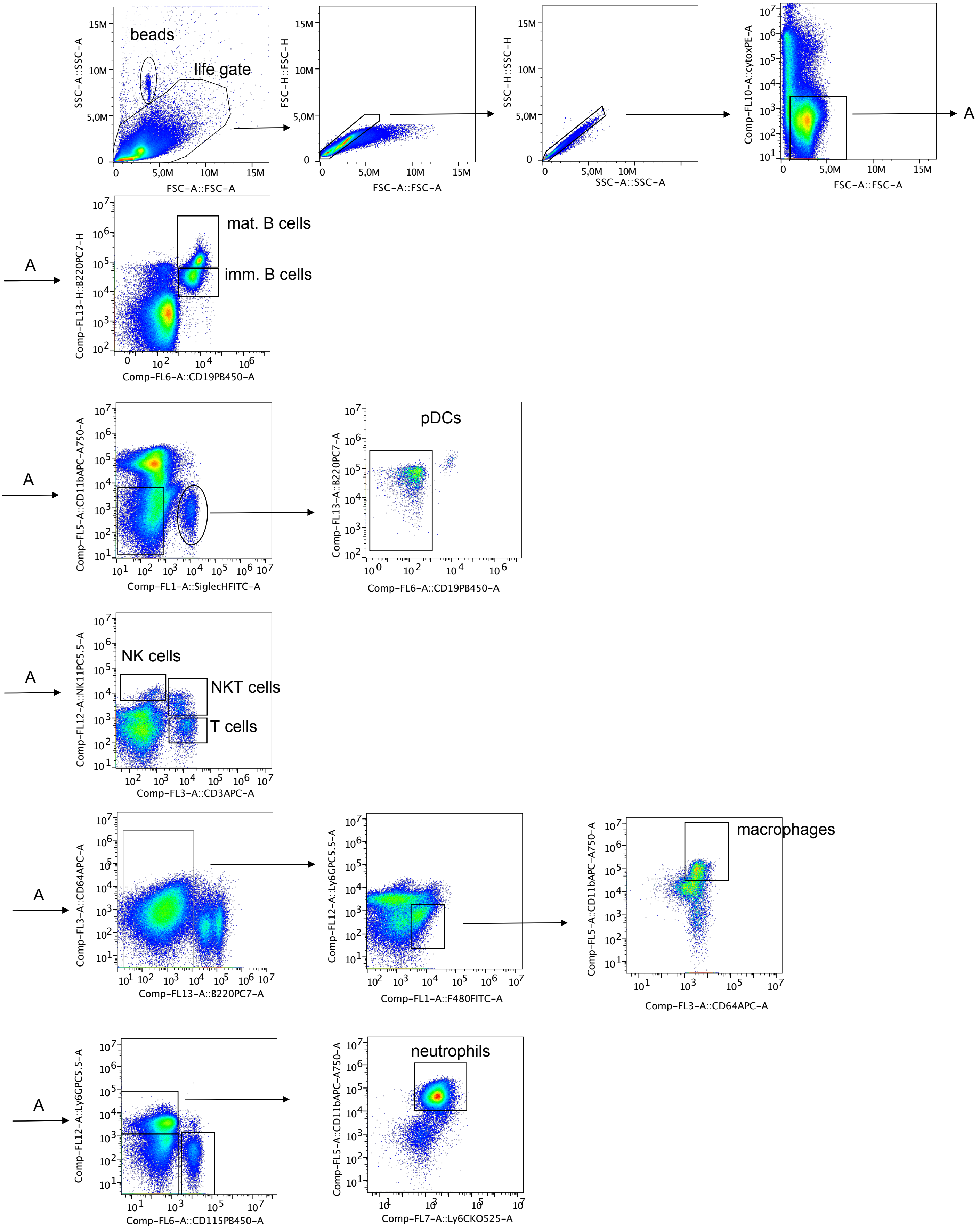

Gating strategy of MK ploidity related to ED Fig. 5e

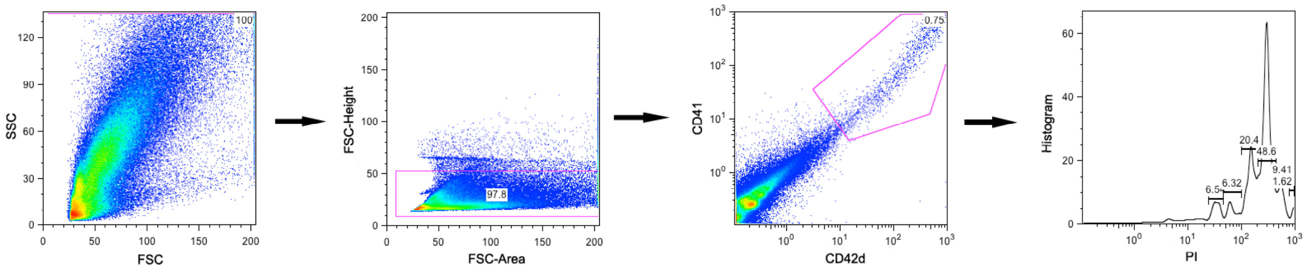

Gating strategy of macrophages and neutrophils related to ED Fig. 5h

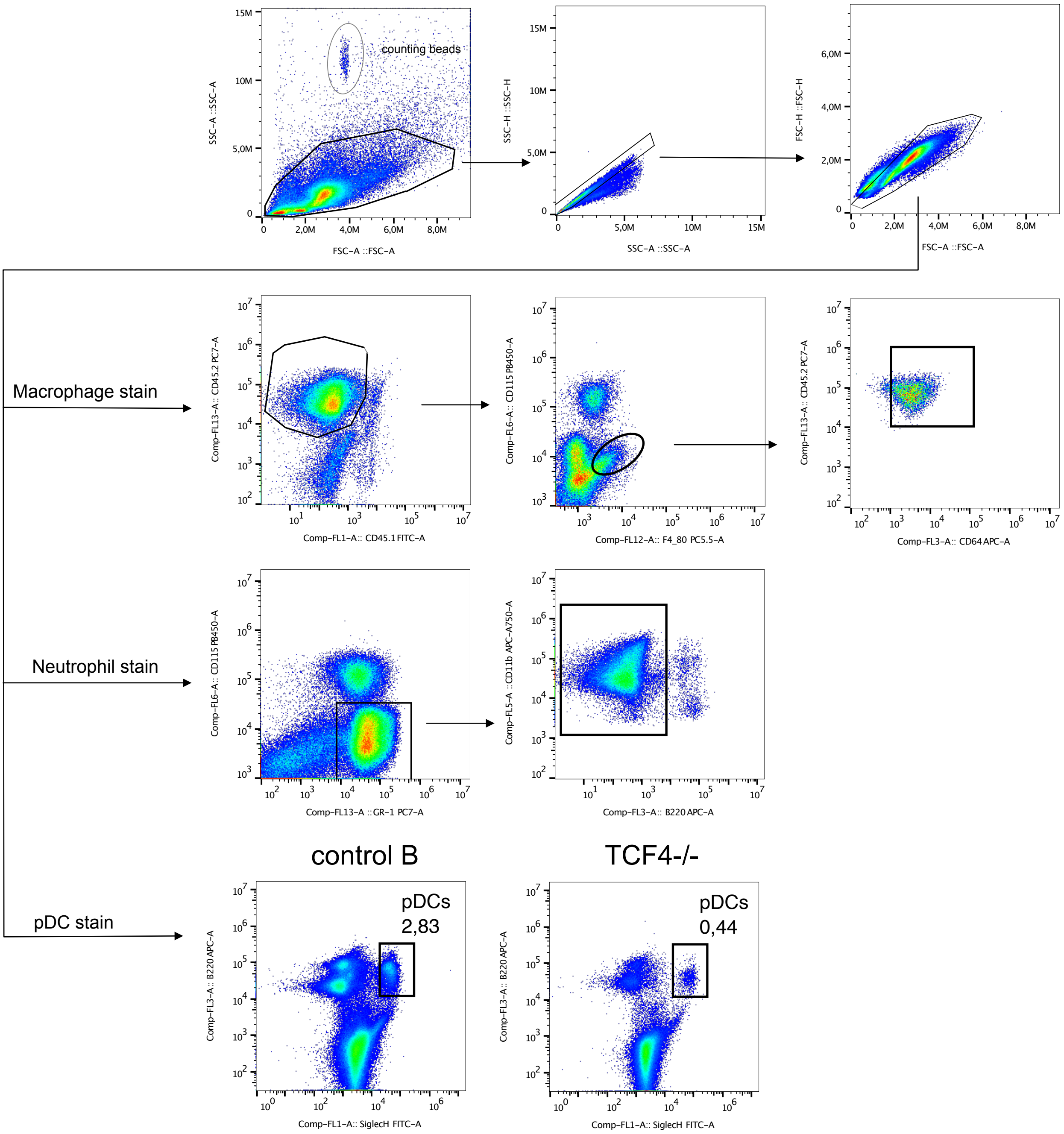

Gating strategy of pDC activation related to ED Fig. 6a

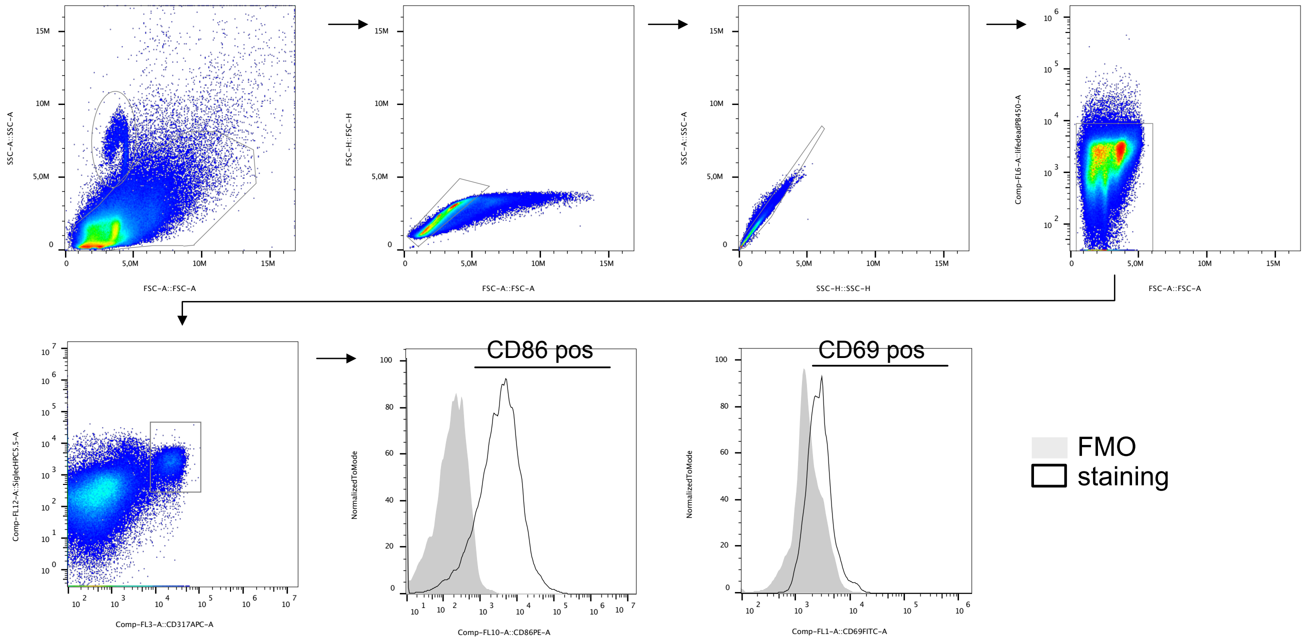

Gating strategy of pDC activation related to ED Fig. 6b

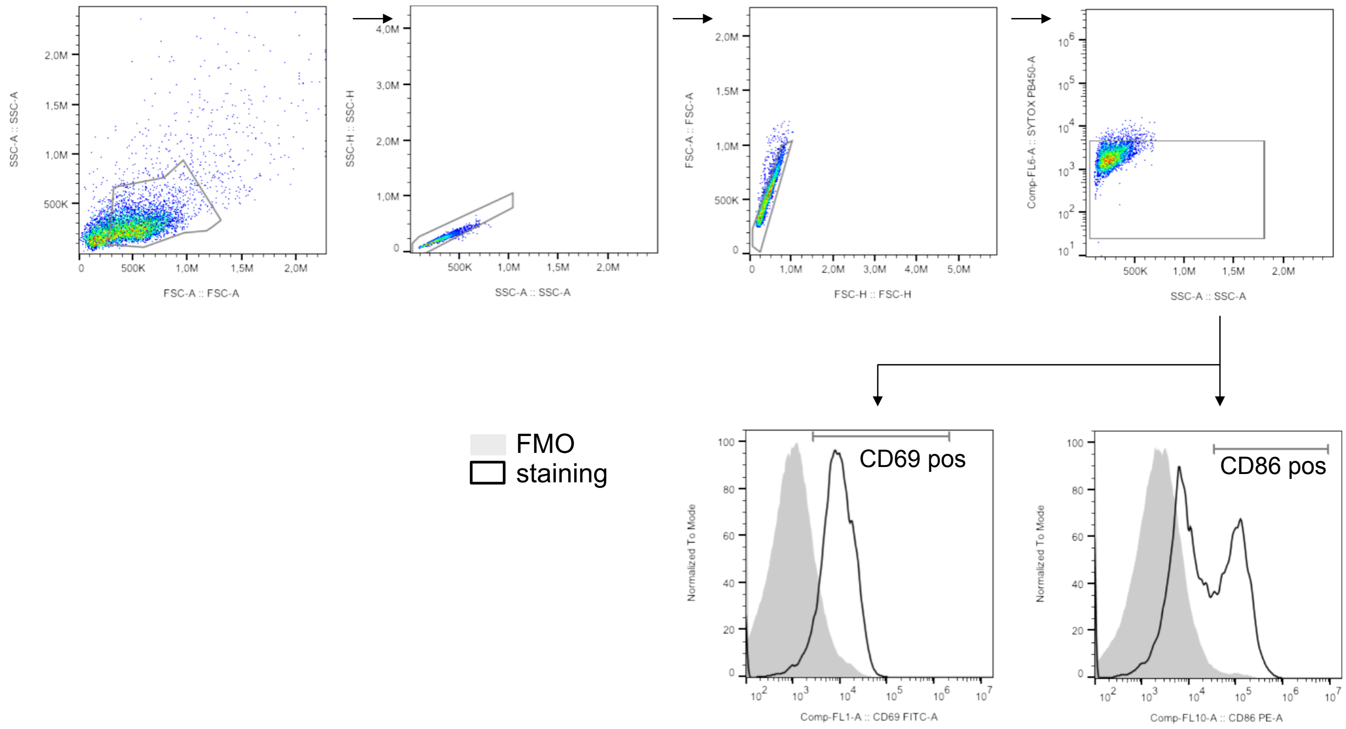

Supplement: Supplementary file 1 — Gating strategies for FACS analysis. [file 41586_2024_7671_MOESM1_ESM.pdf]
