# Supplementary material for: Plasmacytoid dendritic cells control homeostasis of megakaryopoiesis
Source: Nature. 2024 Jul 10;631(8021):645–53. doi: 10.1038/s41586-024-07671-y (PMC11254756; doi:10.1038/s41586-024-07671-y)
Supplement: Supplementary file 2 — Reporting Summary [file 41586_2024_7671_MOESM2_ESM.pdf]

nature portfolio

Corresponding author(s): Florian Gaertner

Last updated by author(s): May 14, 2024

# Reporting Summary

Nature Portfolio wishes to improve the reproducibility of the work that we publish. This form provides structure for consistency and transparency in reporting. For further information on Nature Portfolio policies, see our Editorial Policies and the Editorial Policy Checklist.

## Statistics

For all statistical analyses, confirm that the following items are present in the figure legend, table legend, main text, or Methods section.

| n/a | Confirmed | |
|---|---|---|
| ☐ | ☒ | The exact sample size (*n*) for each experimental group/condition, given as a discrete number and unit of measurement |
| ☐ | ☒ | A statement on whether measurements were taken from distinct samples or whether the same sample was measured repeatedly |
| ☐ | ☒ | The statistical test(s) used AND whether they are one- or two-sided<br>*Only common tests should be described solely by name; describe more complex techniques in the Methods section.* |
| ☒ | ☐ | A description of all covariates tested |
| ☐ | ☒ | A description of any assumptions or corrections, such as tests of normality and adjustment for multiple comparisons |
| ☐ | ☒ | A full description of the statistical parameters including central tendency (e.g. means) or other basic estimates (e.g. regression coefficient) AND variation (e.g. standard deviation) or associated estimates of uncertainty (e.g. confidence intervals) |
| ☐ | ☒ | For null hypothesis testing, the test statistic (e.g. *F*, *t*, *r*) with confidence intervals, effect sizes, degrees of freedom and *P* value noted<br>*Give P values as exact values whenever suitable.* |
| ☒ | ☐ | For Bayesian analysis, information on the choice of priors and Markov chain Monte Carlo settings |
| ☒ | ☐ | For hierarchical and complex designs, identification of the appropriate level for tests and full reporting of outcomes |
| ☒ | ☐ | Estimates of effect sizes (e.g. Cohen's *d*, Pearson's *r*), indicating how they were calculated |

*Our web collection on statistics for biologists contains articles on many of the points above.*

## Software and code

Policy information about availability of computer code

| | |
|---|---|
| Data collection | ZEN Black 2.3 SP1 (confocal microscope) and ZEN blue 2.6 softwares (Axiozoom microscope) (https://www.zeiss.com), CytExpert 2.4 software Beckman Coulter (Cytoflex-S), BD FACSDiva software version 6.0 (BD FACS Canto II) and 7.0 (BD FACS ARIA III Cell sorter), BD FACSChorus version 1.1.20.0 (BD FACS Melody Cell sorter), FACs DIVA software version 8.0.1 (LSR Fortessa FACS), ImSpector Pro 275 (Multiphoton microscope LaVision), Vectra Polaris version 1.0.5 (PerkinElmer microscope). Autopsies sample acquisition (developed in the frame of the German Registry of COVID-19 autopsies, www.DeRegCOVID.ukaachen.de). |
| Data analysis | Imaris software version 9.2.1 from OXFORD instruments (https://imaris.oxinst.com), Zen blue software Zeiss version 2.3 (https://www.zeiss.com/microscopy/en/products/software/zeiss-zen-lite.html), FlowJo TM versions 10.6.2 or 10.9.<br>scRNAseq: CellRanger version 6.0.2, Seurat version 4.0.4, Monocle3, Code is available under (https://github.com/heiniglab/gaertner_megakaryocytes), DESeq2 Version 1.30.0, GSEA version 4.0.3, DAVID version 6.8, GraphPad Prism software (9.1.2, San Diego, USA) , HALO software (Indica labs) version 3.2.1851, Noise2void (Fiji plugin), Fiji software (Nature Methods, 9(7), 676–682. doi:10.1038/nmeth.2019), ClustVis (http://biit.cs.ut.ee/clustvis), Leiden algorithm version 2.6. |

For manuscripts utilizing custom algorithms or software that are central to the research but not yet described in published literature, software must be made available to editors and reviewers. We strongly encourage code deposition in a community repository (e.g. GitHub). See the Nature Portfolio guidelines for submitting code & software for further information.

## Data

Policy information about availability of data

All manuscripts must include a data availability statement. This statement should provide the following information, where applicable:

- Accession codes, unique identifiers, or web links for publicly available datasets
- A description of any restrictions on data availability
- For clinical datasets or third party data, please ensure that the statement adheres to our policy

Data that supports the findings of this study are available within the article and its Supplementary Information. Any additional information and related data are available upon reasonable request. Bulk RNA-Seq data is accessible under a GEO database entry GSE185488. scRNA-Seq data is accessible under a GEO database entry (GSE261996)

## Research involving human participants, their data, or biological material

Policy information about studies with human participants or human data. See also policy information about sex, gender (identity/presentation), and sexual orientation and race, ethnicity and racism.

| | |
|---|---|
| Reporting on sex and gender | Both genders were equally represented in all our analyses. |
| Reporting on race, ethnicity, or other socially relevant groupings | not applied |
| Population characteristics | see supplementary table 2 |
| Recruitment | Both genders were equally represented in all our analyses. We analyzed autopsy specimens from 12 patients who died from COVID-19. All patients were diagnosed with COVID-19 ante mortem and PCR-tests for SARS-CoV-2 from postmortem nasopharyngeal swabs were positive. Patients died from respiratory failure caused by SARS-CoV-2 infection and were picked randomly to exclude any self-selection bias. Bone marrow biopsies of ITP and lymphoma patients were chosen for the best possible match with respect to age and sex. Clinical Details of the patients are given in supplementary table 2. |
| Ethics oversight | The study was approved by and conducted according to requirements of the ethics committees at the Ludwig Maximilians University of Munich (20-1039) and the local ethics committee (EK 304/20, EK 119/20, and EK 092/20). |

Note that full information on the approval of the study protocol must also be provided in the manuscript.

# Field-specific reporting

Please select the one below that is the best fit for your research. If you are not sure, read the appropriate sections before making your selection.

☒ Life sciences ☐ Behavioural & social sciences ☐ Ecological, evolutionary & environmental sciences

For a reference copy of the document with all sections, see nature.com/documents/nr-reporting-summary-flat.pdf

# Life sciences study design

All studies must disclose on these points even when the disclosure is negative.

| | |
|---|---|
| Sample size | Established experimental group sizes from previous work were used to approximate sample sizes (Massberg et al. Nature Medicine;16:887–896(2010); Gaertner et al. Cell;171(6):1368-1382(2017). |
| Data exclusions | No data was excluded from analysis. |
| Replication | Replication of all data was successful. All data were replicated at least 3 times (RNAseq experiments excluded), see figure legends for recapitulations of experiments. |
| Randomization | No randomization was performed; all cells or mice assigned to control or experimental group were analyzed. For the in vivo experiments mice were allocated by age and sex- matched between control and experimental group to have a consistent pared controls. |
| Blinding | The investigators were not blinded to allocation during the experiments and outcome assessment. Blinding was not possible for the in vitro and the in vivo studies as these experiments were performed by individual investigators who were aware of the experimental groups and treatment outcome. |

# Reporting for specific materials, systems and methods

We require information from authors about some types of materials, experimental systems and methods used in many studies. Here, indicate whether each material, system or method listed is relevant to your study. If you are not sure if a list item applies to your research, read the appropriate section before selecting a response.

## Materials & experimental systems

| n/a | Involved in the study | |
|---|---|---|
| ☐ | ☒ | Antibodies |
| ☒ | ☐ | Eukaryotic cell lines |
| ☒ | ☐ | Palaeontology and archaeology |
| ☐ | ☒ | Animals and other organisms |
| ☒ | ☐ | Clinical data |
| ☒ | ☐ | Dual use research of concern |
| ☒ | ☐ | Plants |

## Methods

| n/a | Involved in the study | |
|---|---|---|
| ☒ | ☐ | ChIP-seq |
| ☐ | ☒ | Flow cytometry |
| ☒ | ☐ | MRI-based neuroimaging |

# Antibodies

Antibodies used

The following commercially available primary antibodies were use in this study. Company, clone and # catalog number are provide wherever such information is publicly available. Secondary antibodies and dyes are provide in a table on the manuscript. Antibody dilutions are provide in the material & methods section in the manuscript text.

From BioRad Laboratories (Biotechnology Research Hercules, CA)
1. Anti-CD 68 Antibody rat anti-mouse, monoclonal clone (FA-11), BioRad # MCA1957GA
2. Anti-CD41 Antibody anti-human (clone PM6/248), Biorad # MCA467PE

From Abcam (Cambridge, MA, USA)
1. Anti-CD123 anti-human rabbit recombinant monoclonal clone (2947R), Abcam # ab257307
2. Anti-CD41 AntibodyAnti-CD41 Recombinant clone (EPR4330), Abcam # ab134131

From Biolegend (San Diego, CA, USA)
1. Anti-CD42d Antibody purified anti-mouse/rat, clone (1C2), BioLegend # 148501
2. Anti-CD42d Antibody anti-mouse/rat APC, clone (1C2), BioLegend # 148506
3. Anti-PDCA-1 (BST2) Antibody purified Ultra-LEAFTM anti-mouse, clone (927), BioLegend # 127030
4. Anti-CD 64 Antibody anti-mouse APC, clone (X54-5/7.1,) BioLegend # 139306
5. Anti-CD105 Antibody anti-mouse, PE/Cyanine7, clone (MJ7/18), BioLegend # 120410
6. Anti-CD115 (CSF-1R) Antibody anti-mouse, Brilliant Violet 421™, clone (AFS98), BioLegend # 135513
7. Anti-CD117 (c-Kit) Antibody anti-mouse, APC, clone (2B8) BioLegend # 105812
8. Anti-CD11b Antibody anti-mouse/human, APC/Cyanine7, clone (M1/70), BioLegend # 101226
9. Anti-CD11b Antibody anti-mouse/human, Pacific Blue™, clone (M1/70), BioLegend # 101224
10. Anti-CD11b Antibody anti-mouse/human, PE/Cyanine7, clone (M1/70), BioLegend # 101216
11. Anti-CD150 (SLAM) Antibody anti-mouse, Brilliant Violet 510™ clone (TC15-12F12.2), BioLegend # 115929
12. Anti-CD3 Antibody anti-mouse, Pacific Blue™, clone (17A2), BioLegend # 100214
13. Anti-CD317 (BST2, PDCA-1) Antibody anti-mouse, APC, clone (927), BioLegend # 127016
14. Anti-CD45.1 Antibody anti-mouse, FITC, clone (A20), BioLegend # 110706
15. Anti-CD45.2 Antibody anti-mouse, PE/Cyanine7, clone (104), BioLegend # 109830
16. Anti-CD69 Antibody anti-mouse, FITC, clone (H1.2F3), BioLegend # 104506
17. Anti-CD86 Antibody anti-mouse,PE, clone (GL-1), BioLegend # 105008
18. Anti-CD9 Antibody anti-mouse, PerCP/Cyanine5.5, clone (MZ3), BioLegend # 124818
19. Anti-F4/80 Antibody anti-mouse, clone (BM8), PerCP/Cyanine5.5, BioLegend # 123128
20. Anti-Ly-6A/E (Sca-1) Antibody anti-mouse, clone (E13-161.7), PE/Cyanine7, BioLegend # 122514
21. Anti-Ly-6G Antibody anti-mouse, Pacific Blue™, clone (1A8), BioLegend # 127612
22. Anti-Ly-6G/Ly-6C (Gr-1) Antibody anti-mouse PE/Cyanine7, clone (RB6-8C5), BioLegend # 108416
23. Anti-SiglecH Antibody anti-mouse PerCP/Cyanine5.5, clone (551), BioLegend # 129614
24. Anti-SiglecH Antibody anti-mouse monoclonal PE, (clone 551), BioLegend # 129606
25. Anti-SiglecH Antibody anti-mouse monoclonal FITC, clone (551), BioLegend # 129603
26. Anti-TER-119 Antibody anti-mouseclone Pacific Blue™, clone (TER-119), BioLegend # 116232
27. Rat IgG2b Purified Ultra-LEAF™ Purified Rat IgG2b, κ Isotype Ctrl, (clone RTK4530), BioLegend # 400671
28. Anti-CD41 Antibody anti-mouse FITC, clone (MWReg30), Biolegend # 133903
29. Anti- IFNAR-1 Antibody anti-mouse, clone (MAR1-5A3), Biolegend #127302
30. Anti- CD45R/B220 Antibody anti-mouse/human APC, clone (RA3-6B2), Biolegend # 103212
31. Anti- CD45R/B220 Antibody anti-mouse/human Pacific blue, clone (RA3-6B2), Biolegend # 103227
32. Anti-CD117 (c-Kit) Antibody anti-mouse, APC/Cyanine7clone (2B8) BioLegend # 105826
33. Anti- CD8 Antibody anti-mouse/human Pacific blue, clone (53-6.7), Biolegend # 100725
34. Anti-F4/80 Antibody anti-mouse FITC, clone (BM8), Biolegend # 123108
35. Anti- Ly-6G Antibody anti-mouse PerCP/Cyanine5.5, clone (1A8), Biolegend # 127616
36. Anti- CD45R/B220 Antibody anti-mouse/human PE/Cyanine7, clone (RA3-6B2), Biolegend # 103222
37. Anti- Ly-6C Antibody anti-mouse Brilliant Violet 510™, clone (HK1.4), Biolegend # 128033
38. Anti- CD3 Antibody anti-mouse APC, clone (17A2), Biolegend # 100236
39. Anti- CD19 Antibody anti-mouse Brilliant Violet 421™, clone (6D5), Biolegend # 115537

From Thermo Fisher Scientific/eBioscience (Waltham, MA, USA)
1. Anti-CD11b Antibody monoclonal (M1/70), Biotin clone (M1/70), eBioscience # 13-0112-82

2. Anti-CD45R (B220) Antibody anti-human/mouse monoclonal (RA3-6B2), Biotin, ThermoFisher # 13-0452-82
3. Anti-CD69 Antibody CD69 anti-human monoclonal clone (8B6), ThermoFisher # MA5 15612
4. Anti-IFN alpha Antibody anti-Human, Mouse, Rat Polyclonal clone (P01562), ThermoFisher # PA5-115430
5. Anti-Ly-6G Antibody anti-mouse Monoclonal Biotin, clone (1A8-Ly6g), eBioscience # 13-9668-82
6. Anti-CD317 (BST2, PDCA-1) Antibody anti-mouse monoclonal, clone (eBio927), Functional Grade eBioscience # 16-3172-81
7. Anti-BST2 (CD 317), anti-mouse/human polyclonal antibodies, ThermoFisher #PA5-120152
8. Anti-TER-119 Antibody anti-mouse monoclonal biotin, clone (TER-119), eBioscience # 13-5921-82,
9. Anti-VE-cadherin Antibody anti-mouse monoclonal biotin, (clone eBioBV13), eBioscience # 13-1441-82
10. Anti-CD3 Antibody anti-human/mouse monoclonal biotin, clone (OKT3), eBioscience # 13-0037-82
11. Anti-CD41 Antibody anti-mouse clone FITC, (eBioMWReg30 (MWReg30)), eBioscience # 11-0411-82

From Cell Signaling Technology (Danvers, MA, USA)
1. Anti-phospho-IRF7 Antibody (Ser437/438) Rabbit, clone (D6M2I), Cell Signaling Technology #24129

From BD BioscienceTM (Franklin Lakes, New Jersey, USA)
1-Anti-CD16/CD32 (Mouse BD Fc Block™), Purified Rat Anti-Mouse clone (2.4G2), BD Bioscience# 553142

From BD PharmingenTM (San Diego, California, USA)
1. Anti- NK-1.1 Antibody anti-mouse PerCP-Cy™5.5, clone (PK 136), BD Pharmingen™ # 561111

From Bio X Cell (Lebanon, NH, USA)
1. Anti-IFNAR-1 Antibody anti-mouse clone (MAR1-5A3), BioXcell # BE0241

From PBL, Assay Science (Piscataway, New Jersey, United States)
1. IFN Alpha Human Hybrid Protein (Universal Type I IFN), PBL, assay science # 11200-1

From EMFRET Analytics (Eibelstadt, Bayern, Germany)
1. Platelet depletion antibody, emfret # R300
2. Platelet depletion antibody isotype control, emfret # C301

From Akoya Biosciences
1. Opal™ 620 Reagent Pack Akoya Biosciences #FP1495001KT
2. Opal™ 650 Reagent Pack Akoya Biosciences #FP1496001KT

Validation

Validation statements for all antibodies listed above can be found through the following links to the manufacture's website.

From BioRad Laboratories (Biotechnology Research Hercules, CA)
1. https://www.bio-rad-antibodies.com/monoclonal/mouse-cd68-antibody-fa-11-mca1957.html
2. https://www.bio-rad-antibodies.com/monoclonal/human-cd41-antibody-pm6-248-mca467.html?f=purified

From Abcam (Cambridge, MA, USA)
1. https://www.abcam.com/en-de/products/primary-antibodies/il3ra-cd123-antibody-il3ra-2947r-ab257307#
2. https://www.abcam.com/en-de/products/primary-antibodies/cd41-antibody-epr4330-ab134131

From Biolegend (San Diego, CA, USA)
1. https://www.biolegend.com/en-us/products/purified-anti-mouse-rat-cd42d-antibody-10199
2. https://www.biolegend.com/de-de/products/apc-anti-mouse-rat-cd42d-antibody-10736
3. https://www.biolegend.com/de-de/products/ultra-leaf-purified-anti-mouse-cd317-bst2-pdca-1-antibody-18420?GroupID=BLG8963
4. https://www.biolegend.com/en-ie/clone-search/apc-anti-mouse-cd64-fcgammari-antibody-7874?GroupID=BLG8810
5. https://www.biolegend.com/nl-nl/products/pe-cyanine7-anti-mouse-cd105-antibody-4573
6. https://www.biolegend.com/nl-nl/products/brilliant-violet-421-anti-mouse-cd115-csf-1r-antibody-8971
7. https://www.biolegend.com/fr-ch/products/apc-anti-mouse-cd117-c-kit-antibody-72?GroupID=BLG1945
8. https://www.biolegend.com/en-ie/products/apc-cyanine7-anti-mouse-human-cd11b-antibody-3930
9. https://www.biolegend.com/en-ie/products/pacific-blue-anti-mouse-human-cd11b-antibody-3863?GroupID=BLG10552
10. https://www.biolegend.com/nl-be/products/pe-cyanine7-anti-mouse-human-cd11b-antibody-1921?GroupID=BLG10427
11. https://www.biolegend.com/de-de/products/brilliant-violet-510-anti-mouse-cd150-slam-antibody-8596
12. https://www.biolegend.com/en-ie/products/pacific-blue-anti-mouse-cd3-antibody-3317?GroupID=BLG6730
13. https://www.biolegend.com/fr-ch/products/apc-anti-mouse-cd317-bst2-pdca-1-antibody-6316
14. https://www.biolegend.com/de-de/products/fitc-anti-mouse-cd45-1-antibody-198
15. https://www.biolegend.com/de-de/products/pe-cyanine7-anti-mouse-cd45-2-antibody-4918
16. https://www.biolegend.com/en-ie/products/fitc-anti-mouse-cd69-antibody-264?GroupID=BLG10536
17. https://www.biolegend.com/en-ie/products/pe-anti-mouse-cd86-antibody-256
18. https://www.biolegend.com/de-de/products/percp-cyanine5-5-anti-mouse-cd9-antibody-17108?GroupID=BLG6313
19. https://www.biolegend.com/nl-nl/products/percp-cyanine5-5-anti-mouse-f480-antibody-4303
20. https://www.biolegend.com/nl-be/products/pe-cyanine7-anti-mouse-ly-6a-e-sca-1-antibody-3898
21. https://www.biolegend.com/en-ie/products/pacific-blue-anti-mouse-ly-6g-antibody 6082?GroupID=BLG5803
22. https://www.biolegend.com/en-gb/products/pe-cyanine7-anti-mouse-ly-6g-ly-6c-gr-1-antibody-1931?GroupID=BLG4876
23. https://www.biolegend.com/en-us/products/percp-cyanine5-5-anti-mouse-siglec-h-antibody-6927
24. https://www.biolegend.com/en-ie/products/pe-anti-mouse-siglec-h-antibody-5178
25. https://www.biolegend.com/de-de/products/fitc-anti-mouse-siglec-h-antibody-5177
26. https://www.biolegend.com/en-gb/products/pacific-blue-anti-mouse-ter-119-erythroid-cells-antibody-6137?GroupID=ImportedGROUP1
27. https://www.biolegend.com/nl-be/products/ultra-leaf-purified-rat-igg2b-kappa-isotype-ctrl-7727

28. https://www.biolegend.com/de-de/products/fitc-anti-mouse-cd41-antibody-5896?GroupID=BLG10424
29. https://www.biolegend.com/de-de/products/purified-anti-mouse-ifnar-1-antibody-4765
30. https://www.biolegend.com/fr-ch/products/apc-anti-mouse-human-cd45r-b220-antibody-442
31. https://www.biolegend.com/nl-nl/products/pacific-blue-anti-mouse-human-cd45r-b220-antibody-2857
32. https://www.biolegend.com/fr-ch/products/apc-cyanine7-anti-mouse-cd117-c-kit-antibody-5905
33. https://www.biolegend.com/de-de/products/pacific-blue-anti-mouse-cd8a-antibody-2856
34. https://www.biolegend.com/fr-ch/clone-search/fitc-anti-mouse-f4-80-antibody-4067?GroupID=BLG5319
35. https://www.biolegend.com/en-gb/products/percp-cyanine5-5-anti-mouse-ly-6g-antibody-6116?GroupID=BLG7234
36. https://www.biolegend.com/en-us/products/pe-cyanine7-anti-mouse-human-cd45r-b220-antibody-1930
37. https://www.biolegend.com/en-us/products/brilliant-violet-510-anti-mouse-ly-6c-antibody-8726?GroupID=BLG5853
38. https://www.biolegend.com/de-at/products/apc-anti-mouse-cd3-antibody-8055
39. https://www.biolegend.com/en-us/products/brilliant-violet-421-anti-mouse-cd19-antibody-7160?GroupID=BLG10556

From Thermo Fisher Scientific/eBioscience (Waltham, MA, USA)
1. https://www.thermofisher.com/antibody/product/CD11b-Antibody-clone-M1-70-Monoclonal/13-0112-82
2. https://www.thermofisher.com/antibody/product/CD45R-B220-Antibody-clone-RA3-6B2-Monoclonal/13-0452-82
3. https://www.thermofisher.com/antibody/product/CD69-Antibody-clone-8B6-Monoclonal/MA5-15612
4. https://www.thermofisher.com/antibody/product/IFN-alpha-Antibody-Polyclonal/PA5-115430
5. https://www.thermofisher.com/antibody/product/Ly-6G-Antibody-clone-1A8-Ly6g-Monoclonal/13-9668-82
6. https://www.thermofisher.com/antibody/product/CD317-BST2-PDCA-1-Antibody-clone-eBio927-Monoclonal/16-3172-81
7. https://www.thermofisher.com/antibody/product/BST-2-Antibody-Polyclonal/PA5-120152
8. https://www.thermofisher.com/antibody/product/TER-119-Antibody-clone-TER-119-Monoclonal/13-5921-82
9. https://www.thermofisher.com/antibody/product/CD144-VE-cadherin-Antibody-clone-eBioBV13-BV13-Monoclonal/13-1441-82
10. https://www.thermofisher.com/antibody/product/CD3-Antibody-clone-OKT3-Monoclonal/13-0037-82
11. https://www.thermofisher.com/antibody/product/CD41a-Antibody-clone-eBioMWReg30-MWReg30-Monoclonal/11-0411-82

From Cell Signaling Technology (Danvers, MA, USA)
1. https://www.cellsignal.com/products/primary-antibodies/phospho-irf-7-ser437-438-d6m2i-rabbit-mab-mouse-specific/24129

From BD BioscienceTM (Franklin Lakes, New Jersey, USA)
1. https://www.bdbiosciences.com/en-de/products/reagents/flow-cytometry-reagents/research-reagents/single-color-antibodies-ruo/purified-rat-anti-mouse-cd16-cd32-mouse-bd-fc-block.553141

BD PharmingenTM (San Diego, California, USA)
1. https://www.bdbiosciences.com/en-us/products/reagents/flow-cytometry-reagents/research-reagents/single-color-antibodies-ruo/percp-cy-5-5-mouse-anti-mouse-nk-1-1.561111

Bio X Cell (Lebanon, NH, USA)
1. https://bioxcell.com/invivomab-anti-mouse-ifnar-1-be0241

PBL, Assay Science (Piscataway, New Jersey, United States)
1. https://www.pblassaysci.com/proteins/human-ifn-alpha-hybrid-protein-universal-type-i-ifn-11200

EMFRET Analytics (Eibelstadt, Bayern, Germany)
1. https://www.emfret.com/fileadmin/user_upload/Datasheets/R300.pdf
2. https://fnkprddata.blob.core.windows.net/domestic/data/datasheet/EMF/C301.pdf

Akoya Biosciences
1. https://my.akoyabio.com/ccrz__ProductDetails?sku=FP1495001KT&cclcl=en_US
2. https://my.akoyabio.com/ccrz__ProductDetails?sku=FP1496001KT&cclcl=en_US

# Animals and other research organisms

Policy information about studies involving animals; ARRIVE guidelines recommended for reporting animal research, and Sex and Gender in Research

| | |
|---|---|
| Laboratory animals | 6-12 weeks-old female or male mice were used from the following mouse strains: C57BL/6J, C57BL/6 CD45.1, PF4-Cre (C57BL/6-Tg(Pf4-icre)Q3Rsko/J), Rosa26-iDTRflox (C57BL/6Gt(ROSA)26Sortm1(HBEGF)Awai/J), IFNαR-/- (B6.129S2-Ifnar1tm1Agt/Mmjax), IFNαR1flox (B6(Cg)-Ifnar1tm1.1Ees/J), BDCA2-DTR (C57BL/6-Tg(CLEC4C-HBEGF)956Cln/J), vWF-Cre, vWF-Cre-GFP, RS26-CreERT2 (B6.129-Gt(ROSA)26Sortm1(cre/ERT2)Tyj/J), Tcf4fl/fl (C57BL/6N-Tcf4tm1c(EUCOMM)Wtsi/WtsiH), LysM-Cre (B6.129P2-Lyz2tm1(cre)Ifo/J), Mcl-1 fl/fl (B6;129-Mcl1tm3Sjk/J), CD11b-DTR (B6.FVB-Tg(ITGAM-HBEGF/EGFP)34Lan/J), Myd88-/- (B6.129P2(SJL)-Myd88tm1.1Defr/J), FVB-K18hACE2 |
| Wild animals | No wild animals were included in this study. |
| Reporting on sex | male and female mice were considered in this study without any discrimination |
| Field-collected samples | No field-collected samples were included in this study. |
| Ethics oversight | All animal experiments were performed in compliance with all relevant ethical regulations for studies involving mice and were |

| | |
|---|---|
| Ethics oversight | approved by the local legislation on protection of animals (Regierung von Oberbayern, Munich). All the covid-19 mice infection were approved by the Authorization n° 270/2022-PR (prot. 6EEAF.228). |

Note that full information on the approval of the study protocol must also be provided in the manuscript.

# Plants

| | |
|---|---|
| Seed stocks | *Report on the source of all seed stocks or other plant material used. If applicable, state the seed stock centre and catalogue number. If plant specimens were collected from the field, describe the collection location, date and sampling procedures.* |
| Novel plant genotypes | *Describe the methods by which all novel plant genotypes were produced. This includes those generated by transgenic approaches, gene editing, chemical/radiation-based mutagenesis and hybridization. For transgenic lines, describe the transformation method, the number of independent lines analyzed and the generation upon which experiments were performed. For gene-edited lines, describe the editor used, the endogenous sequence targeted for editing, the targeting guide RNA sequence (if applicable) and how the editor was applied.* |
| Authentication | *Describe any authentication procedures for each seed stock used or novel genotype generated. Describe any experiments used to assess the effect of a mutation and, where applicable, how potential secondary effects (e.g. second site T-DNA insertions, mosiacism, off-target gene editing) were examined.* |

# Flow Cytometry

## Plots

Confirm that:

☒ The axis labels state the marker and fluorochrome used (e.g. CD4-FITC).

☒ The axis scales are clearly visible. Include numbers along axes only for bottom left plot of group (a 'group' is an analysis of identical markers).

☒ All plots are contour plots with outliers or pseudocolor plots.

☒ A numerical value for number of cells or percentage (with statistics) is provided.

## Methodology

| | |
|---|---|
| Sample preparation | Long bones (femurs, tibiae, humerus) were harvested into ice-cold sterile PBS. Bones were flushed with PBS + 2% fetal calf serum (FCS) using a 26-Gauge needle and the bone marrow suspension was further filtered through a 100µm cell strainer (Miltenyl Bioec GmbH, Bergisch Gladbach, Germany) and pelleted at 4degree celsius, 300xg for 5min. The supernatant was discarded and cells were resuspended and incubated in red blood cell lysis buffer for 5min. Lysis was terminated by adding 30ml PBS + 2mM Ethylenediaminetetraacetic acid (EDTA, Sigma-Aldrich, Saint Louis, MO, USA), followed by centrifugation 4 degree celsius, 300xg for 5 min. Cells were resuspended with PBS + 0.5% bovine serum albumin (BSA, Sigma-Aldrich, Saint Louis, MO, USA). |
| Instrument | Data was acquired on a Cytoflex-S (Beckman Coulter, Germany), BD FACS Canto II (BD Biosciences, Germany), and BD FACS ARIA III Cell sorter (BD Biosciences, Germany), BD FACS Melody Cell sorter (BD Biosciences, Germany), LSR Fortessa FACS (BD Biosciences, Germany) |
| Software | Data was acquired with CytExpert 2.4 software Beckman Coulter (Cytoflex-S), BD FACSDiva software version 6.0 (BD FACS Canto II) and 7.0 (BD FACS ARIA III Cell sorter), BD FACSChorus version 1.1.20.0 (BD FACS Melody Cell sorter), FACs DIVA software version 8.0.1 (LSR Fortessa FACS). The analysis was performed with FlowJo T 10.6.2 or 10.9 software. |
| Cell population abundance | Cell abundance is approximately 2-3% of all CD45+ cells in Bone marrow for pDCs and 0.05%-1% for MKs and Mkps, the sorted cells were sorted in "PCR custom mode" with 1000 cells sorted in NEBlysis buffer. Purity was measured and guaranteed with additionally sorted cells. |
| Gating strategy | Gating strategies are available as supplementary data 1 |

☒ Tick this box to confirm that a figure exemplifying the gating strategy is provided in the Supplementary Information.
