# Supplementary material for: Plasmacytoid dendritic cells control homeostasis of megakaryopoiesis
Source: Nature. 2024 Jul 10;631(8021):645–53. doi: 10.1038/s41586-024-07671-y (PMC11254756; doi:10.1038/s41586-024-07671-y)
Supplement: Supplementary file 5 — List of all reagents and resources with the source and identifier. [file 41586_2024_7671_MOESM5_ESM.pdf]

REAGENT or RESOURCE	SOURCE	IDENTIFIER
**Antibodies and dyes**		
Anti-CD 64 Antibody anti-mouse, APC clone (X54-5/7.1)	BioLegend	# 139306
Anti-CD 68 Antibody rat anti-mouse monoclonal, clone (FA-11)	BioRad	# MCA1957GA
Anti-CD105 Antibody anti-mouse, PE/Cyanine7, clone (MJ7/18)	BioLegend	# 120410
Anti-CD115 (CSF-1R) Antibody anti-mouse, Brilliant Violet 421™, clone (AFS98)	BioLegend	# 135513
Anti-CD117 (c-Kit) Antibody anti-mouse, APC, clone (2B8)	BioLegend	# 105812
Anti-CD117 (c-Kit) Antibody anti-mouse, APC/Cyanine7, clone (2B8)	BioLegend	# 105826
Anti-CD11b Antibody anti-mouse/human, APC/Cyanine7, clone (M1/70)	BioLegend	# 101226
Anti-CD11b Antibody anti-mouse/human, Pacific Blue™, clone (M1/70)	BioLegend	# 101224
Anti-CD11b Antibody anti-mouse/human, PE/Cyanine7, clone (M1/70)	BioLegend	# 101216
Anti-CD11b Antibody monoclonal (M1/70), Biotin, clone (M1/70)	eBioscience™	# 13-0112-82
Anti-CD150 (SLAM) Antibody anti-mouse, Brilliant Violet 510ᵀ clone (TC15-12F12.2)	BioLegend	# 115929
Anti-CD16/CD32 (Mouse BD Fc Block™), Purified Rat Anti-mouse clone (2.4G2)	BD Bioscience™	# 553142
Anti-CD8 Antibody anti-mouse/human Pacific blue, clone (53-6.7)	Biolegend	# 100725
Anti-CD3 Antibody anti-mouse, Pacific Blue™ clone (17A2)	BioLegend	# 100214
Anti-CD3 Antibody anti-mouse APC, clone (17A2)	Biolegend	# 100236
Anti-CD3 Antibody monoclonal (OKT3), Biotin clone (OKT3)	eBioscience™	# 13-0037-82
Anti-CD317 (BST2, PDCA-1) Antibody anti-mouse monoclonal Functional Grade, clone (eBio927)	eBioscience™	# 16-3172-81
Anti-CD317 (BST2), Antibody anti-mouse/human, rabbit polyclonal	ThermoFisher	#PA5-120152
Anti-CD317 (BST2, PDCA-1) Antibody anti-mouse, APC, clone (927)	BioLegend	# 127016
Anti-PDCA-1 (BST2) purified Ultra-LEAF™, clone (927)	BioLegend	# 127030
Anti-CD123 Antibody anti-human CD123, clone (2947R)	Abcam	# ab257307
Anti-CD41 Antibody anti-human (clone PM6/248)	BioRad	# MCA467GA
Anti-CD41 Antibody anti-mouse FITC, clone (eBioMWReg30 (MWReg30))	eBioscience™	# 11-0411-82
Anti-CD41 Antibody anti-mouse FITC, clone (MWReg30)	Biolegend	# 133903
Anti-CD41 Antibody ant-human/mouse recombinant, clone (EPR4330)	Abcam	# ab134131
Anti-CD42d Antibody anti-mouse/rat APC, (clone 1C2)	BioLegend	# 148506
Anti-CD42d Antibody purified anti-mouse/rat, (clone 1C2)	BioLegend	# 148501
Anti-CD45.1 Antibody anti-mouse FITC, clone (A20)	BioLegend	# 110706
Anti-CD45.2 Antibody anti-mouse PE/Cyanine7, clone (104)	BioLegend	# 109830
Anti-CD45R (B220) Antibody monoclonal, Biotin, clone (RA3-6B2)	ThermoFisher	# 13-0452-82
Anti-CD45R (B220) Antibody anti-mouse APC, clone (RA3-6B2)	BioLegend	# 103212
Anti-CD45R (B220) Antibody anti-mouse, Pacific Blue, clone (RA3-6B2)	BioLegend	# 103227

Anti-CD45R/B220 Antibody anti-mouse/human PE/Cyanine7, clone (RA3-6B2)	Biolegend	# 103222
Anti-CD69 Antibody anti-human monoclonal, clone (8B6)	ThermoFisher	# MA5 15612
Anti-CD69 Antibody anti-mouse FITC, clone (H1.2F3)	BioLegend	# 104506
Anti-CD86 Antibody anti-mouse PE, clone (GL-1)	BioLegend	# 105008
Anti-CD9 Antibody anti-mouse PerCP/Cyanine5.5, clone (MZ3)	BioLegend	# 124818
Anti-CD19 Antibody anti-mouse Brilliant Violet 421™, clone (6D5)	Biolegend	# 115537
Anti-F4/80 Antibody anti-mouse PerCP/Cyanine5.5, clone (BM8)	BioLegend	# 123128
Anti-F4/80 Antibody anti-mouse FITC, clone (BM8)	Biolegend	# 123108
Anti-IFN alpha Antibody polyclonal, clone (P01562)	ThermoFisher	# PA5-115430
Anti-IFNAR-1 Antibody anti-mouse, clone (MAR1-5A3)	BioXcell	# BE0241
Anti-IFNAR-1 Antibody anti-mouse, clone (MAR1-5A3)	Biolegend	#127302
Anti-phospho-IRF7 Antibody (Ser437/438) rabbit, clone (D6M2I)	Cell Signaling Technology	# 24129
Anti-Ly-6A/E (Sca-1) Antibody anti-mouse PE/Cyanine7, clone (E13-161.7)	BioLegend	# 122514
Anti-Ly-6G Antibody anti-mouse Monoclonal Biotin, clone (1A8-Ly6g)	eBioscience	# 13-9668-82
Anti-Ly-6G Antibody anti-mouse Pacific Blue™, clone (1A8)	BioLegend	# 127612
Anti-Ly-6G Antibody anti-mouse PerCP/Cyanine5.5, clone (1A8)	Biolegend	# 127616
Anti-Ly-6G/Ly-6C (Gr-1) Antibody anti-mouse PE/Cyanine7, clone (RB6-8C5)	BioLegend	# 108416
Anti-Ly-6C Antibody anti-mouse Brilliant Violet 510™, clone (HK1.4)	Biolegend	# 128033
Anti-NK-1.1 Antibody anti-mouse PerCP-Cy™5.5, clone (PK 136)	BD Pharmingen™	# 561111
Anti-SiglecH Antibody anti-mouse PerCP/Cyanine5.5, clone (551)	BioLegend	# 129614
Anti-SiglecH Antibody anti-mouse PE, clone (551)	BioLegend	# 129606
Anti-SiglecH Antibody anti-mouse monoclonal FITC, clone (551)	BioLegend	# 129603
Anti-TER-119 Antibody anti-mousePacific Blue™, clone (TER-119)	BioLegend	# 116232
Anti-TER-119 Antibody monoclonal Biotin, clone (TER-119)	eBioscience™	# 13-5921-82
Anti-VE-cadherin Antibody anti-mouse monoclonal Biotin, clone (eBioBV13)	eBioscience™	# 13-1441-82
IFN Alpha Human Hybrid Protein (Universal Type I IFN)	PBL, assay science	# 11200-1
Platelet depletion antibody	emfret	# R300
Platelet depletion antibody isotype control	emfret	# C301
Rat IgG2b Purified Ultra-LEAF™ Purified Rat IgG2b, κ Isotype Ctrl (clone RTK4530)	BioLegend	# 400671
Apotracker™ Green	BioLegend	# 427402
DAPI (4',6-Diamidino-2-Phenylindole, Dihydrochloride)	ThermoFisher	# D1306
Hoechst 33342, Trihydrochloride, Trihydrate	ThermoFisher	# H3570
Dextran, Cascade Blue™, 10,000 MW, Anionic, Lysine Fixable	ThermoFisher	# D1976
Dextran, Tetramethylrhodamine, 10,000 MW, Lysine Fixable (fluoro-Ruby)	ThermoFisher	# D1817
Donkey anti-Goat IgG (H+L) Cross-Adsorbed Secondary Antibody, Alexa Fluor 647	ThermoFisher	# A-21447

Donkey anti-Mouse IgG (H+L) Highly Cross-Adsorbed Secondary Antibody, Alexa Fluor 555	ThermoFisher	# A-31570
Donkey anti-Rabbit IgG (H+L) Highly Cross-Adsorbed Secondary Antibody, Alexa Fluor 488	ThermoFisher	# A-21206
Goat Anti-Armenian hamster IgG H&L Secondary Antibody, Alexa Fluor 647	Abcam	# ab173004
Goat anti-Mouse IgG (H+L) Highly Cross-Adsorbed Secondary Antibody, Alexa Fluor 555	ThermoFisher	# A-21424
Goat anti-Rabbit IgG (H+L) Cross-Adsorbed Secondary Antibody, Alexa Fluor 594	ThermoFisher	# A-11012
Goat anti-Rabbit IgG (H+L) Cross-Adsorbed Secondary Antibody, APC	ThermoFisher	# A-10931
Goat Anti-Rat IgG H&L, Secondary Antibody, Alexa Fluor 647	Abcam	# ab150159
Streptavidin PE conjugate	eBioscience™	# 12-4317-87
SYTOX™ Orange Dead Cell Stain, for flow cytometry	ThermoFisher	# S34861
LIVE/DEAD™ Fixable Aqua Dead Cell Stain Kit, for 405 nm excitation	ThermoFisher	# L34957
Opal™ 620 Reagent Pack	Akoya Bioscience	# FP1495001KT
Opal™ 650 Reagent Pack	Akoya Bioscience	# FP1496001KT
TotalSeq-B anti-mouse Hashtag antibodies no. 1-9 (anti-mouse)	BioLegend	# B0301-B0309
**Reagents, drugs and recombinant proteins**		
123count eBeads™ Counting Beads	ThermoFisher	# 01-1234-42
2-Mercaptoethanol (50 mM)	ThermoFisher	# 31350010
BSA Albumin Fraction V R98 %, powdered	Carl Roth	# 8076.1
Chromium Next GEM Single Cell 3' reagent kit v3.1	10X Genomics	# CG000206 Rev D
Click-iT™ EdU Alexa Fluor™ 647 Flow Cytometry Assay Kit	ThermoFisher	# C10419
Collagen Solution	StemCell	# 04902
Corn Oil	Sigma-Aldrich	# C8267
Cyano Venner dental fast glue	Hager Werken	# 152261
Dako Fluorescence Mounting Medium	DAKO	# S3023
Diphtheria Toxin	Sigma-Aldrich	# 322326
DNase I 100 U (1U/µl)	Invitrogen	# 18068-015
Ethylenediaminetetraacetic acid (EDTA)	Sigma-Aldrich	# 60-00-4
EasySep™ Mouse CD11b Positive Selection Kit II	STEMCELL	# 18970
EasySep™ Mouse CD19 Positive Selection Kit II	STEMCELL	# 18954
ELISA Kit Mouse Thrombopoietin Quantikine	R&D systems	# MTP00
ELISA Kit, Mouse IFN Alpha All Subtype, High Sensitivity (Serum, Plasma, TCM)	PBL, assay science	# 42115-1
Formaldehyde 4%	Microcos GmbH	# 50-00-0
Gibco™ RPMI 1640 Medium, GlutaMAX™ Supplement	ThermoFisher	# 11554516
High-Capacity cDNA Reverse Transcription Kit	Applied Biosystems	# 4368814
Lysing Buffer	BD Bioscience	# 555899
MegaCult™-C Complete Kit Without Cytokines	StemCell	# 04900
Gibco™ MEM Non-Essential Amino Acids Solution (100X)	ThermoFisher	# 11140035
Mouse recombinant thrombopoietin	Immunotools	# 12343615
Normal Goat Serum (10%)	ThermoFisher	# 50197Z

PBS (10X)	Gibco	# 70011-036
PBS (1X)	Gibco	# 14190-094
Penicillin-Streptomycin-Glutamine (100X)	ThermoFisher	# 10378016
Control (Rodend Diet)	Research DIETS	#D10001i<br>AIN-76A
PLX 5622 (1.200 ppm) Rodend Diet	Research DIETS	#D19101002i<br>AIN-76
Propidium Iodide Solution	BioLegend	# 421301
Recombinant Mouse FLT3L (carrier-free)	BioLegend	# 550706
RNeasy Micro Kit	QIAGEN	# 74004
Sodium Pyruvate (100 mM)	ThermoFisher	# 11360070
SsoAdvanced Universal SYBR Green Supermix	Biorad	# 172-5271
Sucrose	Sigma-Aldrich	# S1888
Tamoxifen	Sigma-Aldrich	# 10540-29-1
Thiazol-Orange	Sigma-Aldrich	# 390062
Tissue Tek	Sakura Finetek	
**Experimental models: Organisms/strains**		
C57BL/6J	Jackson Laboratory	
C57BL/6J (CD45.1)	Jackson Laboratory	
PF4-Cre (C57BL/6-Tg(Pf4-icre)Q3Rsko/J)	Jackson Laboratory	(ref 56)
Rosa26-iDTR^{flox} *(C57BL/6*^{Gt}*(ROSA)26*^{Sortm1}*(HBEGF)*$^{Awai/J}$*)*	Jackson Laboratory	(ref 57)
IFNαR-/- (*B6.129S2-Ifnar1*$^{tm1Agt/Mmjax}$)	Jackson Laboratory	(ref 58)
IFNαR1^{flox} *(B6(Cg)-Ifnar1*$^{tm1.1Ees}$*/J)*	Jackson Laboratory	(ref 59)
BDCA2-DTR (*C57BL/6-Tg(CLEC4C-HBEGF)956*$^{Cln/J}$*)*	Jackson Laboratory	(ref 24)
vWF-Cre	Aird lab	(ref 65)
vWF-Cre-GFP	Nerlov lab	(ref 10)
RS26-Cre^{ERT2} *(B6.129-Gt(ROSA)26Sor*$^{tm1(cre/ERT2)Tyj}$*/J)*	Jackson Laboratory	(ref 60)
Tcf4fl/fl (C57BL/6N-Tcf4$^{tm1c(EUCOMM)Wtsi}$*/WtsiH)*	Wellcome Sanger Institute	(ref 66)
LysM-Cre (B6.129P2-Lyz2$^{tm1(cre)Ifo}$*/J)*	Jackson Laboratory	(ref 63)
Mcl-1 fl/fl (B6;129-Mcl1^{tm3Sjk}*/J)*	Jackson Laboratory	(ref 64)
CD11b-DTR (B6.FVB-Tg(ITGAM-HBEGF/EGFP)34Lan/J)	Jackson Laboratory	(ref 62)
Myd88-/- (B6.129P2(SJL)-Myd88$^{tm1.1Defr}$*/J)*	Jackson Laboratory	(ref 61)
FVB-K18hACE2	Iannacone lab	(ref 67)
**Software and Algorithmus**		
Imaris Software	Oxford/Instruments/Imaris	Version 9.2.1
ZEN Software Black and Blue	Carl Zeiss	Versions 2.3 and 2.6

ImSpector Pro 275	LaVision BioTec GmbH	Version 245
Vectra Polaris platform	PerkinElmer	Version 1.0.5
HALO software	Indica labs	Version 3.2.1851
CytExpert software	Beckman Coulter	Version 2.4
FACSDiva software	BD Biosciences	Versions 6.0 and 7.0
FACSChorus software	BD Biosciences	Version 1.1.20.0
FlowJo	BD Biosciences	Versions 10.9 and 10.6.2
GraphPad Prism software	San Diego, USA	Version 9.1.2
Noise2void	Krull et. al (ref 69)	Version 0.3.2
HALO	Indica Lab	Version 3.2.1851
DESeq2	Love et al. (ref 71)	Version 1.30.0
GSEA	https://www.gsea-msigdb.org/gsea/index.jsp	Version 4.0.3
DAVID	Huang et al. (ref 75)	Version 6.8
ClustVis	Metsalu and Vilo (ref 76)	Online version http://biit.cs.ut.ee/clustvis
CellRanger	10X Genomics	Version 6.0.2
Seurat	Stuart et al. (ref 78)	Version 4.0.4
Leiden algorithm	https://neo4j.com	Version 2.6
Monocle3	Cao et al. (ref 80)<br>Trapnell et al. (ref 81)<br>https://cole-trapnell-lab.github.io/monocle3/	Version 1.0.0
